# Fluorescent Nanoparticles for Super-Resolution Imaging

**DOI:** 10.1021/acs.chemrev.2c00050

**Published:** 2022-06-27

**Authors:** Wei Li, Gabriele S. Kaminski Schierle, Bingfu Lei, Yingliang Liu, Clemens F. Kaminski

**Affiliations:** † Key Laboratory for Biobased Materials and Energy of Ministry of Education, College of Materials and Energy, South China Agricultural University, Guangzhou 510642, People’s Republic of China; ‡ Department of Chemical Engineering and Biotechnology, University of Cambridge, Cambridge CB3 0AS, United Kingdom

## Abstract

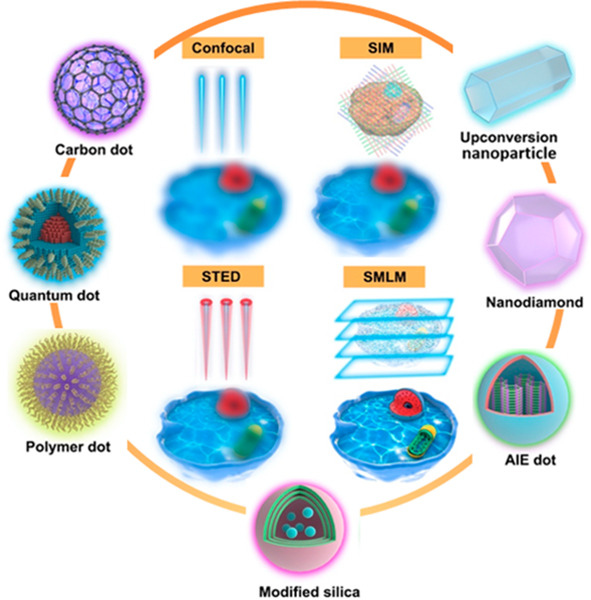

Super-resolution imaging techniques that overcome the diffraction limit of light have gained wide popularity for visualizing cellular structures with nanometric resolution. Following the pace of hardware developments, the availability of new fluorescent probes with superior properties is becoming ever more important. In this context, fluorescent nanoparticles (NPs) have attracted increasing attention as bright and photostable probes that address many shortcomings of traditional fluorescent probes. The use of NPs for super-resolution imaging is a recent development and this provides the focus for the current review. We give an overview of different super-resolution methods and discuss their demands on the properties of fluorescent NPs. We then review in detail the features, strengths, and weaknesses of each NP class to support these applications and provide examples from their utilization in various biological systems. Moreover, we provide an outlook on the future of the field and opportunities in material science for the development of probes for multiplexed subcellular imaging with nanometric resolution.

## 1 Introduction

Fluorescence microscopy has become the standard tool for the study of biological specimens on a small scale, providing both sensitivity and specificity. A drawback is that diffraction limits the lateral resolution of fluorescence microscopes to λ/2*NA*,^1^ where λ is the wavelength of light, and *NA* is the numerical aperture of the objective lens. For typical conditions, this equates to around 250 nm for visible light, providing insufficient detail for the visualization of many subcellular structures. This limit has been broken by the advent of super-resolution methodologies, which have revolutionized the field of biological imaging.^2−4^ With super-resolution microscopy (SRM) techniques, subcellular structures become observable that could previously only be seen by electron microscopy (EM). However, in contrast to EM, SRM can provide dynamic and molecule-specific information from within living cells. It has revealed complex biological functions, such as protein–protein interactions, motion of biomolecules, organelle dynamics, information on cell metabolism and so on.^5−9^ Common to SRM methods is the use of a photophysical phenomenon to switch between physically discernible fluorescence states. This recognition earned Eric Betzig, William Moerner, and Stefan Hell, the Nobel Prize in Chemistry in 2014. The award was specifically for the development of single-molecule localization microscopy (SMLM) and stimulated emission depletion microscopy (STED) as methods to implement these concepts and for opening the field of optical imaging to the nanoscale domain.^5,8,10−12^

**Figure 1:**
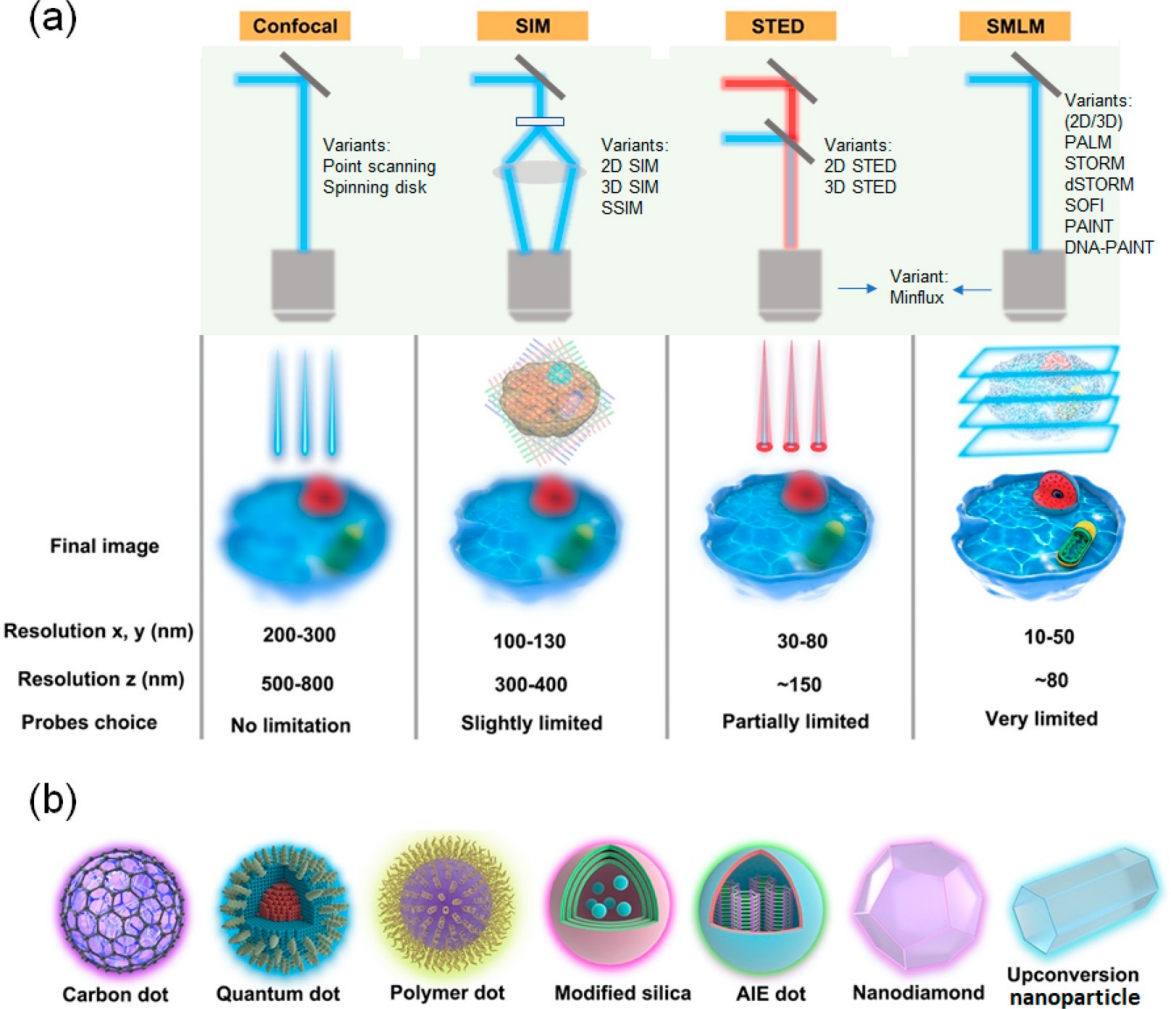
Schematic illustration of NPs used in fluorescence microscopy and comparison of various imaging modalities. (a) The green panel illustrates simplified light-paths to implement confocal imaging, and different super-resolution techniques and their variants. Bottom row: In conventional confocal laser scanning microscopy (CLSM) the image information is gathered sequentially by rastering a focused excitation laser beam across a sample plane (first column). In super-resolution microscopy (SRM), the fluorophores are distinguished by switching between discernible fluorescent states, e.g., on- and off- states. 2nd column: in structured illumination microscopy (SIM), this is achieved through illumination with striped patterns. The spatial modulation of the excitation patterns generates frequency beats with spatial frequencies in the sample. The resulting widefield fluorescence image exhibits so-called Moiré fringes that encode high resolution detail in the low frequency beat patterns. Raw images are collected for different orientations of the illumination pattern. Through mathematical reconstruction, a 2-fold enhancement in spatial resolution can be obtained over wide-field microscopy. 3rd column: in stimulated emission depletion microscopy (STED), fluorophores are returned to an off state by a doughnut shaped beam surrounding the excitation beam. As a result, only fluorophores near the center of the excitation beam emit signal, creating an excitation point spread function (PSF) that is narrower than in the absence of the depletion beam and thus enhanced resolution. Last column: in single-molecule localization microscopy (SMLM), super-resolution is achieved through the sequential imaging of individual fluorophores and inferring the position of emitters through estimation of the centroids of the emission PSFs from individual fluorophores. Photocontrollable fluorophores are required that can be cycled between fluorescent on- and off- states during illumination. Note that the resolution stated for the individual techniques are indicative only and may vary with experimental setups and fluorophore properties. SSIM, saturated structured-illumination microscopy; PALM, photoactivated localization microscopy; *d*STORM/STORM, direct stochastic optical reconstruction microscopy/stochastic optical reconstruction microscopy; SOFI, super-resolution optical fluctuation microscopy; PAINT, points accumulation for imaging in nanoscale topography; MINFLUX, minimal emission fluxes. (b) Schematic makeup of various fluorescent NPs, including carbon dot, quantum dot, polymer dot, modified silica nanoparticle, aggregation-induced emission (AIE) dot, nanodiamond, and upconversion nanoparticle.

SRM techniques are commonly categorized into three groups. One group makes use of a nonlinear fluorescence response to enhance resolution, such as STED^13−17^ and ground state depletion microscopy (GSD).^18−20^ In another, one relies on the photoswitching or photoblinking characteristics of fluorescent molecules and trades temporal resolution with spatial resolution to localize single molecules with enhanced precision. These methods are referred to as single-molecule localization microscopies (SMLMs)^21,22^ and include (fluorescence) photoactivated localization microscopy (FPALM/PALM),^23,24^ and (direct) stochastic optical reconstruction microscopy (*d*STORM/STORM).^25,26^ A related method is based on super-resolution optical fluctuation microscopy (SOFI)^27−29^ and this also depends on the cycling of molecules through physically distinguishable states.^30^ The third group refers to structured illumination microscopy (SIM).^31^ Here, one generates a modulated excitation pattern in the sample and achieves super-resolution by encoding high frequency spatial detail in the sample in low frequency beat patterns that can be computationally processed to reveal subwavelength scale sample detail.^32,33^ Mixtures and combinations of these methods are also possible. For example, saturated structured-illumination microscopy (SSIM) combines patterning of the excitation light and a nonlinear fluorescence response.^34,35^ Minimal emission fluxes (MINFLUX)^36^ is a new technique proposed by the Hell laboratory, combining aspects of SMLM and STED. These techniques have provided new insights into subcellular systems with unprecedented spatial and temporal resolution, leading to breakthroughs in the life- and natural sciences.^23,37,38^ The principles of these super-resolution methods are illustrated in Figure 1a.

Advances in super-resolution imaging techniques have gone hand in hand with the development of fluorescent probes in the biosciences. Their purpose is to act as labels by specific attachment to the biomolecules of interest and permitting their imaging at improved resolution. The performance of SRM techniques, for example relating to image resolution, contrast, and signal-to-noise ratio, depends critically on the properties of the fluorescent probes used. Furthermore, the specific nature of individual SRM techniques places constraints on their photophysical characteristics. Dyes and fluorescent proteins are commonly used in the biosciences but have limitations in their photophysical properties and environmental factors can limit their brightness and proneness to photobleaching.^39,40^ Hence, improvements in the photobrightness, the flexibility of labeling modalities, the photostability of probes, and control over the length of off-states (nonemitting states) for SMLM are all highly desirable for progress in the field. Fluorescent nanoparticles (NPs) offer promise in this endeavor. They can feature favorable optical properties compared to traditional labels and their small sizes (between 1 and 100 nm) lead to strong electron confinement, which enhance quantum effects that can be exploited in rational probe design.^41^ Synthetic NPs can be designed to feature high brightness across the full visible spectrum and their outstanding photostability makes them superior substitutes for existing probes. Common to all NPs used in biological microscopy is an intrinsically fluorescent core with a surface that is modified and functionalized to enable target specific and biocompatible labeling. There is a large parameter space to explore in the rational design of NPs for SRM methodologies. These include absorption and emission cross sections and spectra, photoswitching and blinking properties, target specificity, etc.^42^ While the development and use of organic dyes and fusion proteins for SRM has matured,^43−50^ there are huge opportunities still for novel NPs in SRM. Progress requires the merging of expertise from materials engineering, physics, and chemistry. Only a few review articles have so far focused on fluorescent NPs for SRM imaging,^51−55^ but these were either specific to individual types of NPs or limited to specific application areas. A comprehensive review of the current state of the art and different approaches in the field is thus timely.

**Figure 2:**
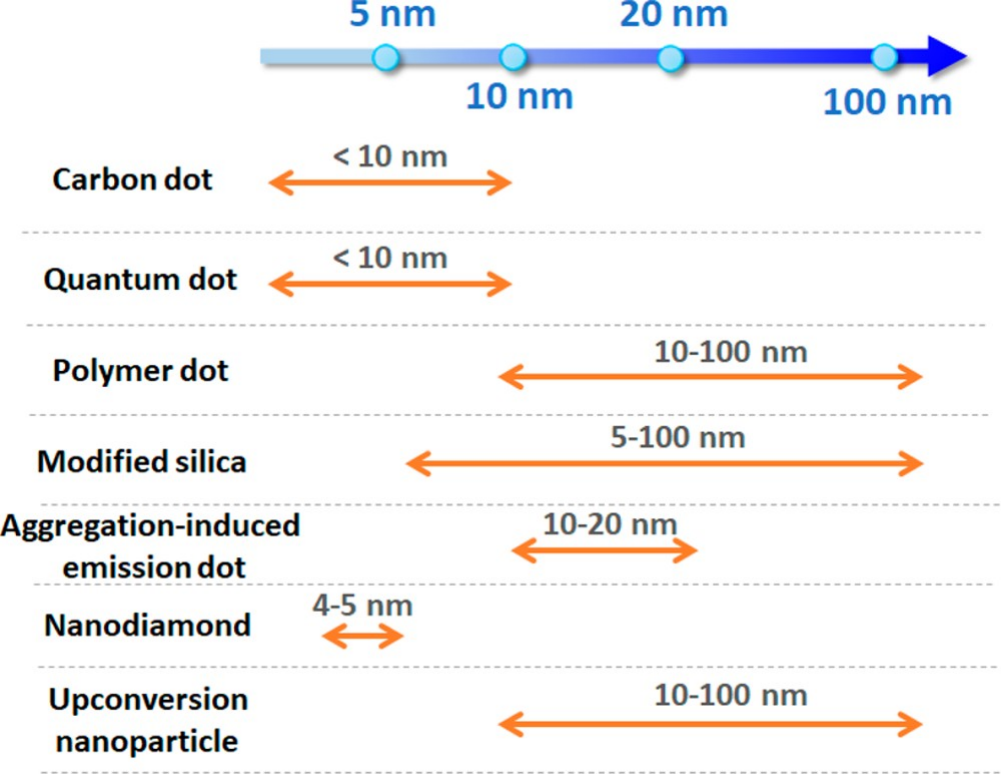
Comparison of typical sizes of different fluorescent nanoparticles (NPs) for use in super-resolution microscopy.

In this review, we summarize promising developments in NP research for subwavelength resolution microscopy. We discuss the material science behind NPs with a specific focus on their properties and use for optimized super-resolution imaging in the biological sciences. We cover carbon dots (CDs), quantum dots (QDs), polymer dots (PDs), modified silica NPs, aggregation-induced emission (AIE) dots, nanodiamonds (NDs), and upconversion nanoparticles (UCNPs) (Figure 1b and Table 1). We describe their spectroscopic properties important for intracellular imaging at the nanoscale, including particle sizes (Figure 2), fluorescence mechanisms, brightness, photostability, and photoswitching kinetics. We discuss promise and opportunities, but also problems and limitations. We conclude with strategies for the surface modification of NPs to achieve desired functional characteristics. We also review bioconjugation strategies for the attachment of NPs to biomolecules, membranes, and subcellular organelles. Finally, we provide an outlook on potential directions for the field and the potential for future improvement of NPs for their use in the study of molecular mechanisms in health and disease.

**Table 1:** Comparison of Different NPs for Super-Resolution Imaging

Nanoparticle Type	Advantages	Disadvantages
Carbon dot	Water-soluble and biocompatible	Lack red and infrared emission
	Easy surface functionalization	Broad excitation and emission bandwidths
	Photostable	
Quantum dot	Tunable particle size and surface modification	Potential toxicity of heavy metals
	Tunable PL emission	Broad absorption band and risk of multiphoton excitation
	Narrow emission band	Short off-state times
	High PL quantum yield	
	Photostable	
Polymer dot	Versatile function and structure	Large particle size
	Easy functionalization	Potential toxicity of degradation products
	Bright and photostable	
	Continuous fluorescence or photoblinking	
Modified silica	Biocompatible	Relies on doped fluorophores
	Easy surface functionalization	Potential toxicity of degradation products
	Enhanced fluorescence effects	
	Efficient carrier for biomolecular cargo	
	Photostable	
Aggregation-induced emission dot	Tunable particle size	Limited choice
	Tunable surface functionality	Poor water solubility
	Photostable	Low PL quantum yield in NIR-II region
Nanodiamond	Biocompatible	Relative large particle size
	Bright and stable	Application in bioimaging is rarely developed
	Long-wavelength emission with high PL quantum yield	Limited emission wavelength
Upconversion nanoparticle	Sharp emission band	Poor water solubility
	PL emission penetrates deep tissue	Low PL quantum yield
	Avoid background autofluorescence	Excitation/emission bands are nearly invariable
	Photostable	Potential photothermal effect
		Potential toxicity of metals
Carbon nanotube	Emission in NIR-I and NIR-II windows	Poor water solubility
	Adjustable absorption range	
	Easy surface functionalization	
	Good as cargo carriers	
Metal-based nanoparticle	Surface plasmon resonance effect	Potential for low colloidal stability
	Suitable for different imaging modalities	Potential toxicity of metals
	Easy surface functionalization	
	Size dependent properties	

## 2 Super-Resolution Imaging Methods

Various physical phenomena are exploited to achieve optical super-resolution, i.e. the resolution of spatial detail below the diffraction limit given by λ/*NA*, where λ is the emission wavelength, and *NA* is the numerical aperture of the signal collecting objective. Each method places specific demands on fluorescent probe design. In the following we give a brief introduction on the principle of different SRM methods (Figure 1) to provide a context for the required photophysical properties of NPs.

### 2.1 Structured Illumination Microscopy

Structured illumination microscopy, SIM, employs a patterned illumination to reconstruct information from beat patterns between sample and illumination spatial frequencies. Interference patterns can be produced to modulate spatial frequencies in 2 dimensions across the sample plane (2D SIM) and in 3 dimensions (3D SIM) (Figure 1a). The technique can achieve a 2-fold linear resolution increase in all spatial dimensions where the excitation intensity is modulated and yields a much improved image contrast compared to widefield imaging.^12,56^ It is the fastest SRM method available but results in a smaller theoretical resolution improvement compared to alternative techniques;^57^ however, it features favorable photon-efficiencies compared to STED and SMLM and requires relatively low excitation intensities. It is thus the most widely used SRM technique for the imaging of dynamic processes in living cells.^58,59^ The low light doses required for SIM keep phototoxicity at tolerable levels in many practical situations. A further advantage is that conventional fluorophores can be used for SIM imaging.^60^ In the case of saturated structured illumination microscopy, SSIM, a better than 2-fold resolution increase can be achieved. The reason for this is that the sample responds in a nonlinear fashion to the excitation modulation, thereby generating higher spatial frequencies (harmonics) in the fluorescence response, that carry information on subwavelength sample detail. The resulting resolution increase comes at the cost of higher excitation powers and longer signal integration times, and photobleaching and phototoxicity become concerns for biological imaging applications. Samples for SIM imaging are prepared in the same way as for conventional fluorescence imaging, but good results require a high fluorophore brightness (defined as the product of the molar extinction coefficient and the fluorescence quantum yield) to permit faithful reconstruction of object information at high recording speeds.^59,61−64^ High image contrast and a good modulation depth of the illumination pattern are essential for the avoidance of artifacts in SIM reconstructions, which are exacerbated by low signal-to-noise ratios.^65,66^ Bright and photostable fluorophores are essential for optimal deployment of the technique. For biological imaging, SIM has offered dynamic information on the function of subcellular organelles in the size range from 100 to 200 nm, including mitochondria, endoplasmic reticulum (ER), lysosomes, centrosomes, nuclei, and so on. The technique has also been used to study of the formation and function of large macromolecular structures, for example protein aggregates and the DNA replication machinery, both of which have been investigated by SIM in live cells.^9,67−73^

### 2.2 Stimulated Emission Depletion Microscopy

In stimulated emission depletion microscopy, STED, a laser beam is focused onto the sample in a confocal microscope to excite the sample fluorophores.^13^ In addition, a doughnut shaped STED beam (also called depletion beam) is arranged to deplete the excited fluorophores in the wings of the excitation beam profile. Its purpose is to deactivate fluorophores in the periphery of the excitation PSF. The result is an effective excitation PSF that is reduced in a spatial extent over which fluorophores produce a signal, thus minimizing blurring and enhancing the resolution. 3D STED is also possible. It provides increased resolution along the optical axis of the microscope in addition to a lateral resolution improvement. The principle is the same as for standard STED, but through use of specialized optics, the depletion light is arranged as a 3-dimensional shell, leading to a strongly confined excitation spot in its center.^74^ A high intensity in the STED beam is critical for efficient depletion and resolution improvement. The resolution of the STED image is proportional to the square root of the power in the depletion beam and good performance requires high depletion intensities. To reduce photodamage, variants of cw and pulsed STED have been developed in efforts to balance phototoxicity and resolution for practical imaging.^75,76^ STED routinely offers a resolution of 30 to 80 nm without a requirement for any image postprocessing. Key to successful STED imaging is minimal cross talk between the depletion process and fluorophore excitation. To avoid the (unwanted) excitation of fluorophores by the STED beam, its wavelength should be red-shifted into a region that is completely outside of the excitation band of the fluorophores used. Good STED fluorophores should thus display a large Stokes shift in their fluorescence spectrum. Furthermore, depletion should be performed in a spectral window where phototoxicity is minimal to the sample and, crucially, where technology for high power lasers is available. This limits the number of efficient STED dyes and imposes criteria for the design of efficient NPs. STED NPs should thus feature large Stokes shifts and need to be highly photostable to resist photobleaching caused by the powerful STED beam, especially for long-term dynamic imaging in live cells or for the acquisition of 3-dimensional image stacks in fixed samples. Two-color imaging can be performed with STED, but the constraints discussed on dyes and lasers make multicolor imaging more challenging than with other SRM methods. The point scanning nature of the method limits acquisition speed as in confocal microscopy, but rapid imaging over small imaging windows is possible,^77^ and dynamic structures such filaments, moving vesicles and other organelles have all been resolved by STED with high contrast and resolution.^77−79^

Several variations on the basic STED concept exist. MINFLUX combines aspects of STED microscopy with single-molecule localization (see next section) and has achieved the highest theoretical resolution of any optical super-resolution method so far. MINFLUX is, however, very challenging to implement experimentally and limited for applications in live biological samples. A technique that is theoretically related to SIM but requires an experimental arrangement similar to STED is called fluorescence emission difference (FED) microscopy.^80^ Here two low power laser beams are used for sequential excitation. Signals are recorded for a Gaussian excitation beam and then a doughnut shaped excitation beam. The resulting images are subtracted from one another yielding a resolution improvement of a factor of 2 over standard confocal microscopy. This is of course much less than what is possible with STED but does not require a high power depletion laser.

### 2.3 Single-Molecule Localization Microscopy

Finally, in single-molecule localization microscopy, SMLM, individual fluorophores are detected and localized from multiple sequentially recorded images of sparse subsets of the sample fluorophore distribution. This is achieved by stochastic switching between two physically distinguishable states (usually a fluorescent on- and an off-state) in photoactivatable or -switchable dyes. Key to a successful deployment of the technique is that the on- to-off ratio of the molecules can be controlled such that in any one image there is a negligible likelihood of two proximate fluorophores to emit simultaneously. This avoids overlap of their emission PSFs. In practice, very small on-to-off ratios are required, and in turn 1000s of images have to be recorded to localize sufficient numbers of molecules to recover high-resolution sample information. SMLM is less demanding to set up compared to SIM and STED and can be implemented with conventional wide-field fluorescence microscopes. Complexities arise, however, from the requirement to optimize sample preparation protocols for a given experiment and the postprocessing of the raw image data.^81^ During imaging, the activation or switching of fluorophores needs to be repeated for many times. NPs should thus meet compatible photophysical requirements, for example, to be reversibly or irreversibly photoswitchable, photoactivatable, or to feature strong photoblinking, permitting the temporal cycling between fluorescent dark and bright states. Desirable characteristics include a high photon output and low on-to-off duty cycles. The labeling density,^82^ switching properties,^83^ linker length of the fluorescent label, and microscope drift all affect the achievable resolution.^84^ In one SMLM variant, called stochastic optical reconstruction microscopy, STORM, the majority of fluorophores are switched into an off-state, leaving only a sparse subset of fluorophores in the on-state. Thus, the off-time of the fluorophore should be much longer than the on-time.^85^ Because of the long acquisition time required to collect a sufficient number of raw images for image reconstruction, the use of STORM for live-cell imaging is not usually possible. A conceptually related method, stochastic optical fluctuation microscopy, SOFI,^27^ relies on the statistical analysis of the signal fluctuations in sequentially acquired fluorescence images to differentiate fluctuations that arise from the blinking of fluorophores from random noise. In contrast to STORM, SOFI permits a higher density of on-state fluorophores, i.e. more than one fluorophore is permitted to be active within an area defined by the detection PSF. Fluorophores whose signals overlap spatially can be distinguished through a temporal correlation analysis of their blinking patterns.^86^ NPs suitable for SOFI thus ought to feature rapid signal fluctuations under constant illumination and feature a high brightness to permit imaging at speed. Other SMLM variants exist. In photoactivated localization microscopy, PALM, photoactivatable fluorophores, usually variants of fluorescent fusion proteins, are used to control the duty cycle of the photon emission, but conceptually there is no difference to STORM imaging (which is usually performed using samples immunolabeled with organic dyes). Another method, points accumulation for imaging in nanoscale topography, PAINT, controls the on- and off-states through physical or chemical control of the residence time of active fluorophores at the site of interest, e.g., through transient binding. In the widely used variant called DNA-PAINT (DNA points accumulation for imaging in nanoscale topography) this is achieved by transient oligonucleotide hybridization. SMLM techniques are capable of localizing isolated macromolecular structures with a lateral resolution of 10 to 50 nm, offering “best in class” performance in this category.^87−90^ However, for volumetric and dynamic imaging, SMLM methods are inferior to the other SRM methods. Figure 1a summarizes the different SRM methods available and their characteristics. Furthermore, Table 1 lists classes of NP materials whose properties may be suitably exploited and optimized for the respective imaging modalities. In the following sections, we present these NPs in detail and review their properties critically in the context of super-resolution imaging.

## 3 Fluorescent Nanoparticles Used in Super-Resolution Microscopy Imaging

### 3.1 Carbon Dots

**Figure 3:**
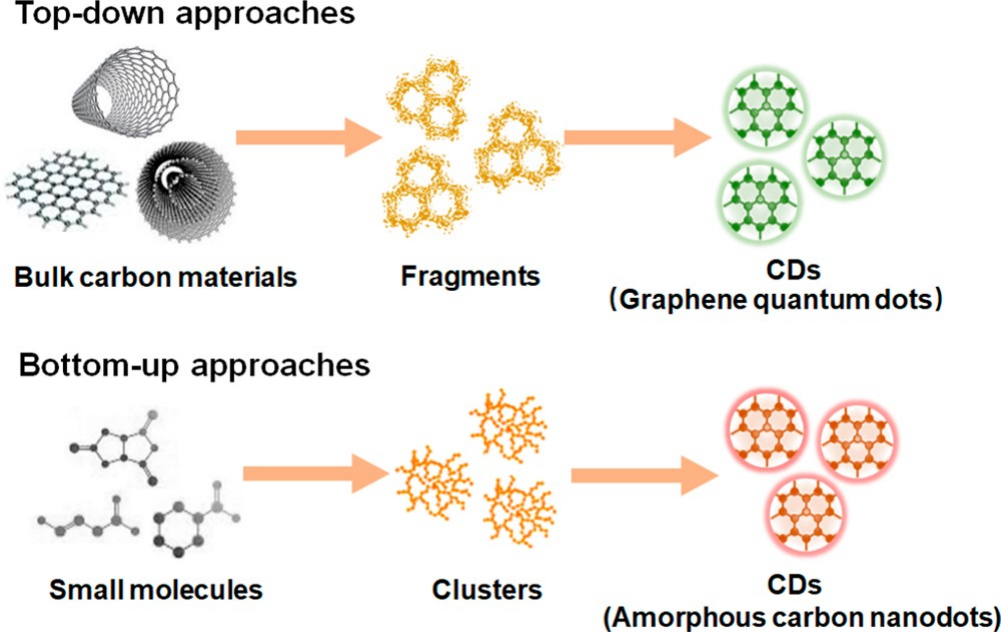
Illustration of production routes for carbon dots (CDs). In top-down methods (top row) a bulk precursor material is fragmented into nm sized carbon dots. Bottom-up synthesis (bottom row) CDs are grown from the assembly of small molecules. Both synthesis routes have advantages and disadvantages, and the produced CDs differ in their photophysical and morphological properties.

**Figure 4:**
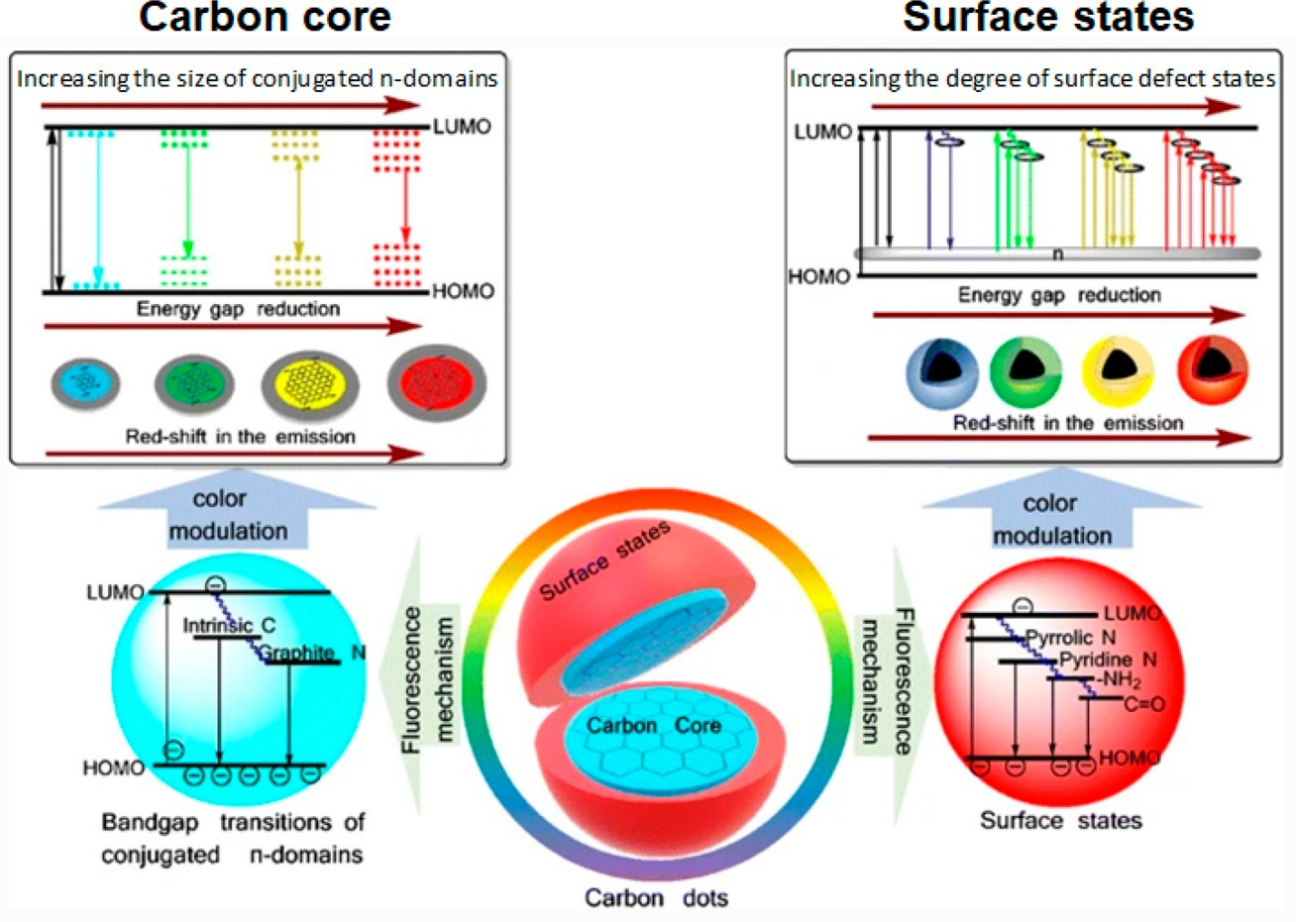
Schematic illustration of the photoluminescence mechanism in CDs. The photospectral properties of CDs are determined by the carbon core and surface states. The band gap of the sp^2^ (graphitic) domain in the carbon core is considered to be the fluorescence center. Adjusting the core size of CDs can thus be used to tune emission properties (left side of the diagram). Chemical groups on the surface of CDs produce defect states, resulting in the creation of new energy levels and emissive traps (right side of figure). Reprinted with permission from ref (96). Copyright 2019 Springer-Verlag GmbH Austria, part of Springer Nature.

Synthetically produced CDs represent a relatively new class of carbon nanomaterial and have attracted significant attention as a promising substitute for traditional organic dyes and QDs in fluorescent imaging.^91^ CDs have notable advantages, such as facile preparation, excellent water solubility, low cytotoxicity, good biocompatibility, and unique optical features, which endow them with excellent potential for bioimaging.^92,93^ The synthesis routes for preparing CDs can be classified into two groups, namely top-down and bottom up approaches (Figure 3).^94^ Both yield CDs that measure typically less than 10 nm in size and quantum confinement effects result in the small CDs attaining their fluorescence properties. As for QDs, the spectral properties depend on confinement, and therefore size. In the top-down approach, a large carbon precursor species is broken down into nanometer sized CDs. CDs prepared in this fashion typically feature large conjugated sp^2^-graphene domains in the carbon core with relatively few surface chemical groups,^95^ and the carbon core is regarded as the fluorescence center (Figure 4, left-hand side).^96^ Increasing the size of the core domain reduces the bandgap with a resulting red-shift in photoluminescence (PL) emission. The bottom-up design, on the other hand, comprises the dehydration, polymerization, and carbonization of small molecules to form CDs with highly configurable physical and chemical properties. The resulting CDs usually present with numerous surface chemical groups, such as −OH, −C=O, −NH_2_, and −COOH. These affect the oxidation state of the CDs and influence the energy levels of the material, via defects and edge states (Figure 4, right-hand side).^97^ Normally, higher degrees of surface oxidation give rise to an increased number of surface defects, which in turn results in an increase in the PL emission wavelength. Furthermore, the presence of heteroatoms such as nitrogen, sulfur, fluorine and so on also affect the energy level structure of CDs. In summary, variation of the carbon core and surface states of CDs leads to tunable photoluminescence characteristics, and provide the means to functionalize the CDs via linker chemistry, for example for targeting biological molecules of interest.^98^

CDs hold promise for super-resolution microscopy in biological systems because of their specific optical properties and their good biocompatibility. The first demonstration of CDs in this context was reported for STED imaging. CDs were dispersed on a coverslip and imaged with STED and a lateral resolution of ca. 30 nm was measured.^99^ The method was then used for imaging MCF7 breast cancer cells incubated with solutions containing CDs. The CDs were taken up efficiently via endocytosis and found to localize in lysosomal compartments within the cells. Here, a resolution of 70 nm was achieved.^99^ Crucially, the CDs exhibited low levels of cytotoxicity and, compared to conventional STED dyes, a superior photostability. However, one problem noticed was the agglomeration of CDs inside endocytic vesicles, and this was the reason for the lower resolution achieved in cells, compared to the *in vitro* sample. To address this issue, NPs can be modified with surface coatings, such as polymers, surfactants, and polyelectrolytes. These improve dispersion stability through a change in surface charge, increasing electrostatic repulsion, or decreasing interfacial energy between NPs and their solvent environment.^100−103^

**Figure 5:**
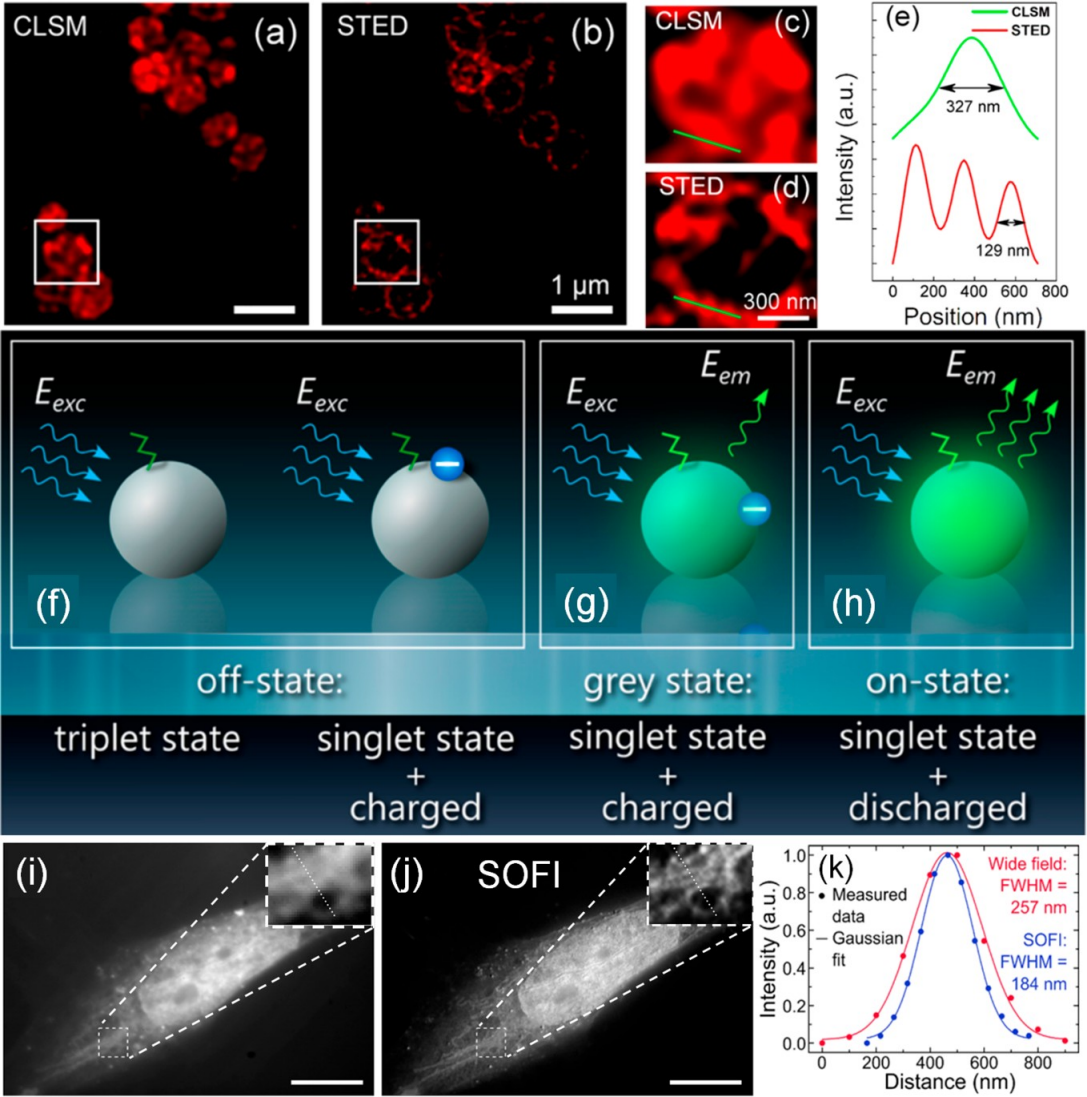
Images of *S. aureus* bacterial cells recorded with, (a) confocal microscopy and, (b) STED subsequently performed. Cells were incubated with CD-C_12_ containing medium for 1 h. Magnified versions of regions designated by the white squares are shown in panels c and d, respectively. The intensity profiles corresponding to the green lines are shown in panel e, demonstrating the resolution enhancement obtained with STED. Panels f–h illustrate different photophysical states thought to occur in CDs. (f) Off-, (g) grey, and (h) on- states that can be exploited for super-resolution imaging. Fluorescence wide-field (i) and second-order SOFI (j) images of a Saos-2 osteoblast-like cell after incubation with blue and green CDs for 1 h. Cells were imaged upon 395 nm excitation using a 405 nm long pass emission filter. Insets show enlarged regions with dotted lines indicating positions for which intensity profiles were measured. Scale bar: 10 μm. (k) Fluorescence intensity profiles for subdiffraction sized features for cross sections indicated in panels i and j. Panels a–e were adapted with permission from ref (104). Copyright 2016 American Chemical Society. Panels f–k were reproduced with permission from ref (107). Copyright 2015 American Chemical Society.

In another study, CDs were conjugated with the quaternary ammonium compound lauryl betaine (BS-12), which has antibacterial properties. The BS-12 modified CDs (CD-C_12_) can be used for detection and inhibition of Gram-positive bacteria (*Staphylococcus aureus*). CD-C_12_ NPs have enabled bacterial imaging with STED, offering an approximately 3-fold resolution enhancement compared to confocal microscopy (Figure 5a–e).^104^ The work was the first example of STED imaging applied to bacteria labeled with CDs. In a recent study cationic CDs were used to label chromatin and nucleoli during cell division and imaged repeatedly over time with STED.^105^ The CDs used measured around 3 nm in size and were seen to diffuse through nuclear membrane pores in live HeLa cells, binding to DNA and RNA, respectively, and yielding spectrally distinguishable fluorescence signals. Although the resulting resolution was not stated, these cationic CDs exhibited greater photostability than Hoechst 33342 dye. More work needs to be done, however, and although CDs used for STED hold promise in terms of biological compatibility and stability, so far no quantitative performance comparisons of CDs with commercially available STED dyes have been reported. In a more recent work, N-, F-codoped CDs with high photoluminescence quantum yield (PLQY) of 56% were utilized for imaging nuclear structure and tunneling nanotubes of 4T1 cells by STED. The CDs were excited at 592 nm wavelength, and depleted with a 660 nm STED beam. The resolution was estimated to be ca. 20 nm for the technique, and nanotubes of ca. 75 nm diameter were easily resolved.^106^

The trapping and redistribution of charges on the surface of CDs trigger transitions between bright and dark states^54,107,108^ and fluorescence time traces can exhibit strong blinking, similar to what is observed for conventional dye molecules.^109^ The blinking rates of CDs obey a power-law distribution, exhibited also in QDs.^110^ The overall behavior of CDs is affected by the chemical groups on the surface and the presence of charge traps. Figure 5f–h shows possible mechanisms for the on-, off-, and gray states in CDs.^107^ The gray state is an intermediate between the dark and brightest states. Figure 5f shows an off-state caused either by transition to a nonradiative triplet state of the surface group, or, alternatively, through a nonradiative energy transfer from the excited state to the trapped charge (Auger recombination, right-hand side of Figure 5f). For the gray states (Figure 5g), energy from the fluorescence center is still transferred to the trapped charge via Auger recombination but only partially, with the result of diminished fluorescence compared to the normal fluorescent on-state (Figure 5h), where the radiative emission is unimpeded by trapped charges. Thus, transition rates are maximized in uncharged CDs, resulting in the highest photoluminescence quantum yield. These principles permit the control of the photoblinking or photoswitching behavior of CDs via electron transfer processes. Figure 5i,j shows how the fluorescence intermittency of CDs can be used for SOFI imaging, here demonstrated for Saos-2 osteoblast cells.^107^ Both green and blue CDs were used in the study and although no specific surface functionalization was performed, it was found that the blue CDs accumulate preferentially in the nucleus of the cells while the green CDs acted as selective labels for endosomes/lysosomes, presumably because of differences in hydrophobicity, surface charge, etc. (Figure 5f–h).^107^ Clearly the SOFI images reveal much greater detail than the widefield images, and cross sections of subdiffraction sized features in the image reveal a lateral resolution of 184 nm (Figure 5k).

The CDs used were found to exhibit characteristics that are a mixture of those of dye molecules and semiconductor nanocrystals. Intriguingly, CDs emitting in the red spectral region were observed to be photoswitchable, which is hypothesized to be caused by an abundance of high energy nonemissive traps on the particle surface, as well as electron transfer processes.^112^ Under constant illumination with light at 639 nm, the fluorescence from individual CDs was seen to photobleach after a certain time period, but subsequent illumination at 401 nm returned the particles back into their photoactive fluorescent state.

**Figure 6:**
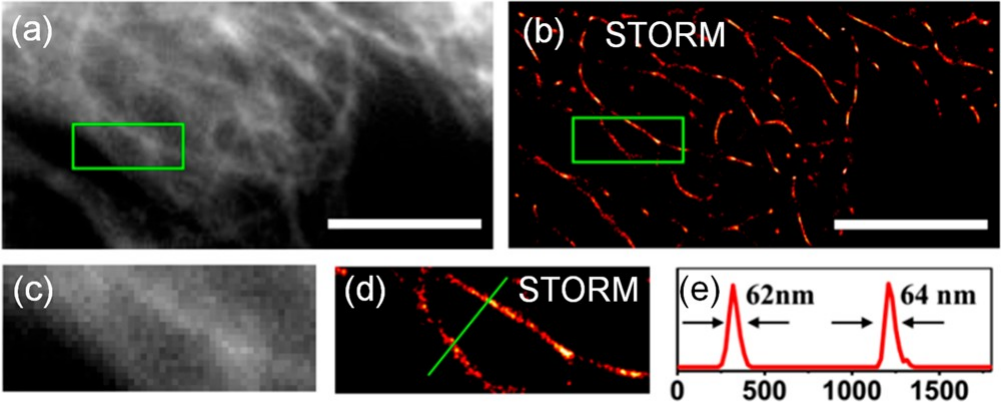
Conventional fluorescence (a) and STORM (b) images of microtubules immuno-stained with CDs, and their corresponding magnified images (c and d), respectively. Scale bar: 10 μm. (e) Intensity profile along line indicated in panel d. Panels a–e were reprinted from ref (111) with permission. Copyright 2017 American Chemical Society.

**Figure 7:**
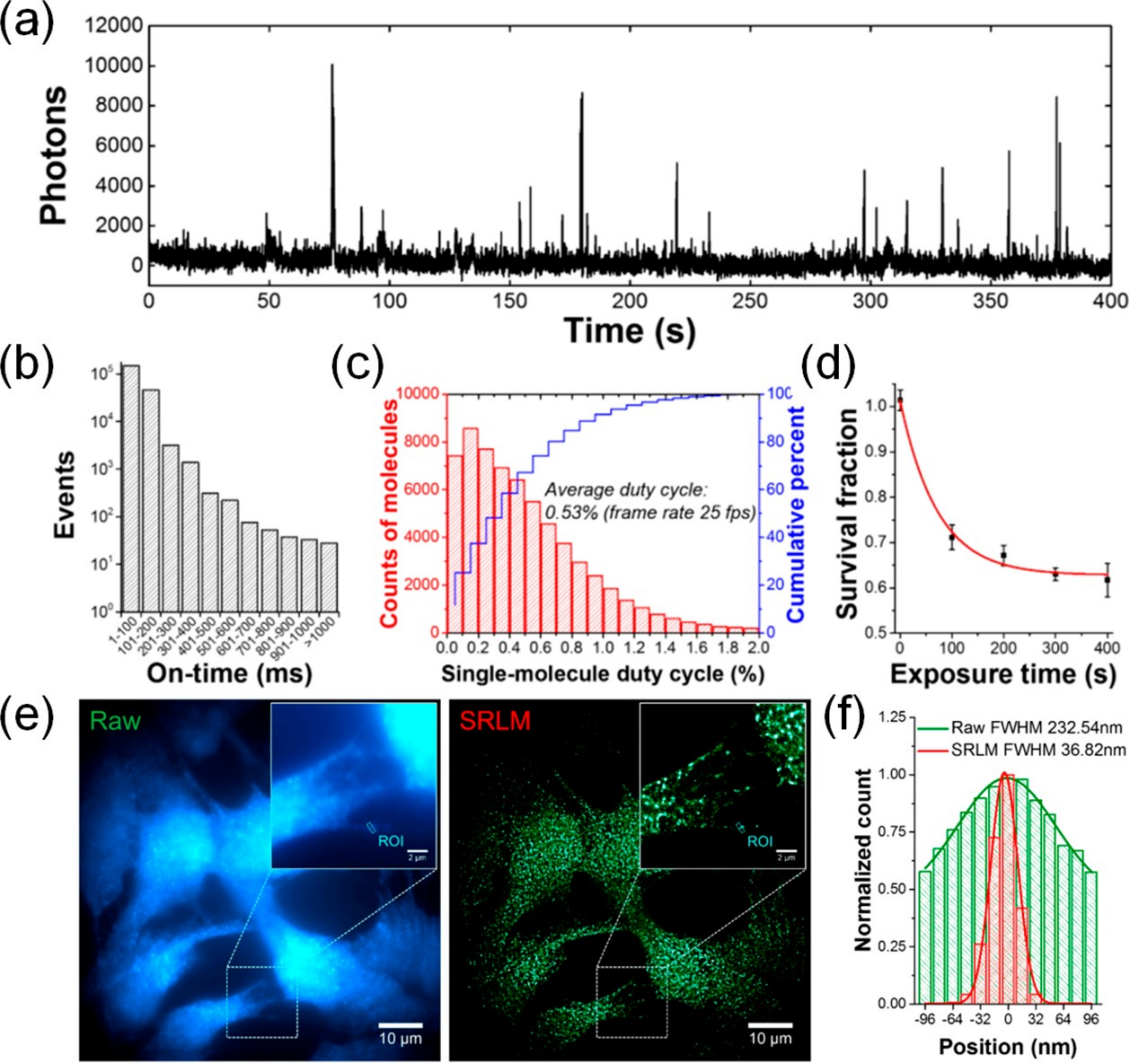
(a) Typical fluorescence time trace of an individual CD. (b) Histogram of the fluorescence on-time distribution from individual CDs. (c) Average fluorescence duty cycles of CDs (>5000 particles). (d) Survival fraction of CDs under high-power green excitation. The graph displays the survival of CDs (fraction of CDs that with exposure time at an illumination power of ca. 0.5 kW/cm^2^. (e) Conventional wide-field fluorescence image (left) and SMLM image (right) of CDs located in fixed trout epithelial gill cells. (f) Comparison of fluorescence emission profiles of intensity features within the indicated regions of interest, ROIs, indicated in panel e. fwhm denotes the full width at half-maximum. Panels a–f were reprinted with the permission from ref (115). Copyright 2018 American Chemical Society.

In another work, a relatively long-lived cationic dark state was observed in CDs when an electron acceptor was present.^113^ The photon budget for such CDs is comparable to that of Cy3 dye, a dye in a similar spectral window that is popular for use in SMLM. Moreover, photoblinking rates in such CDs were seen to be linearly dependent on the power of the bleaching/photoswitching laser, which makes them suitable for SMLM also. A resolution of ∼35 nm was reported to be achievable with such systems.^113^ The authors used nitrogen-doped CDs to label actin filaments, which revealed their self-assembly into soft matter polymer rings, at a resolution of ca. 64 nm.^114^ The authors found that the number of detected photons for CDs was around 3 times lower than for Cy3 in comparable experimental conditions; however, the number of switching cycles was ca. 2.5 times higher. The on–off duty cycles are comparable with that of other reporter dyes or proteins, which again proves promising for the use of CDs in SRMs based on the localization of singe point emitters. In another case, CDs were produced that exhibited photon bursts of high brightness with long intermittency between the bursts in which the CDs were dark.^111^ The CDs exhibited a low duty cycle (∼0.003), high photon output (∼8000 per switching event), and excellent photostability, features that permitted the localization of emitters to within 25 nm. For comparison, conventional fluorophores, such as the organic dyes Cy3, Cy5, and AF647, and commercial CdSe/ZnS QDs were also characterized. It was found that the CDs exhibited a photostability comparable to that of QDs and much higher than that of organic dyes. Most organic molecules were photobleached within 300 s, while CDs and QDs were still fluorescent after 30 min of continuous illumination. In terms of their blinking behavior, CDs displayed a similar photon output and duty cycle as AF647 or Cy5, although QDs produced a larger number of photons. However, for SMLM QDs proved only marginally useful, because of their large duty cycle (∼0.7). The blinking mechanism of CDs has been speculated to occur as follows: the surface states of CDs offer wide and deep traps for accepting the ejected electrons. Trapped electrons are slowly recycled and this then leads to the longevity of the observed “dark” states. In the study by He et al.,^111^ such CDs were used for SMLM of cellular structures and plasma membranes. Other examples are shown in Figure 6a–e, depicting microtubules inside HeLa cells immunostained with primary antibodies and secondary antibodies conjugated with CDs. The microtubules were imaged with STORM and exhibited an excellent gain in resolution compared to what is obtainable with standard imaging. Gaussian fits of intensity profiles across a microtubule measured 60 ± 6 nm (full width half-maximum, fwhm). Recently, CDs prepared from malic acid (MACDs) have shown promise for similar applications.^115^ MACDs deposited on a glass coverslip were also shown to switch stochastically between on and off fluorescence states (Figure 7a). Photoblinking occurs in short burst, and more than 95% of all photoblinking events occur within 200 ms and 75% within 100 ms (Figure 7b), respectively, with an average duty cycle of 0.53% (Figure 7c). More than 60% of the MACDs remained emissive after 400 s of high-power illumination (>0.5 kW/cm^2^) (Figure 7d). The system has good potential for high-resolution SMLM. Figure 7e,f shows SRLM images of CDs in fixed trout epithelial gill cells with more than 6 times better resolution than obtainable with wide-field imaging. In another work, nitrogen-doped CDs were used to label DNA fibers in HeLa cells for imaging by STORM.^116^ Fluorescent CDs were conjugated to actin filaments in HeLa cells, and super-resolution microscopy was performed (STORM and super-resolution radial fluctuation microscopy, SRRF). An approximately 10-fold increase in resolution was obtained over widefield imaging, but in addition the authors demonstrated that the CDs provide contrast in EM. This provides a unique potential for correlative light and electron microscopy, CLEM, using single labels, permitting SRM light microscopy and EM imaging on the same sample via a single labeling strategy.^117^

**Figure 8:**
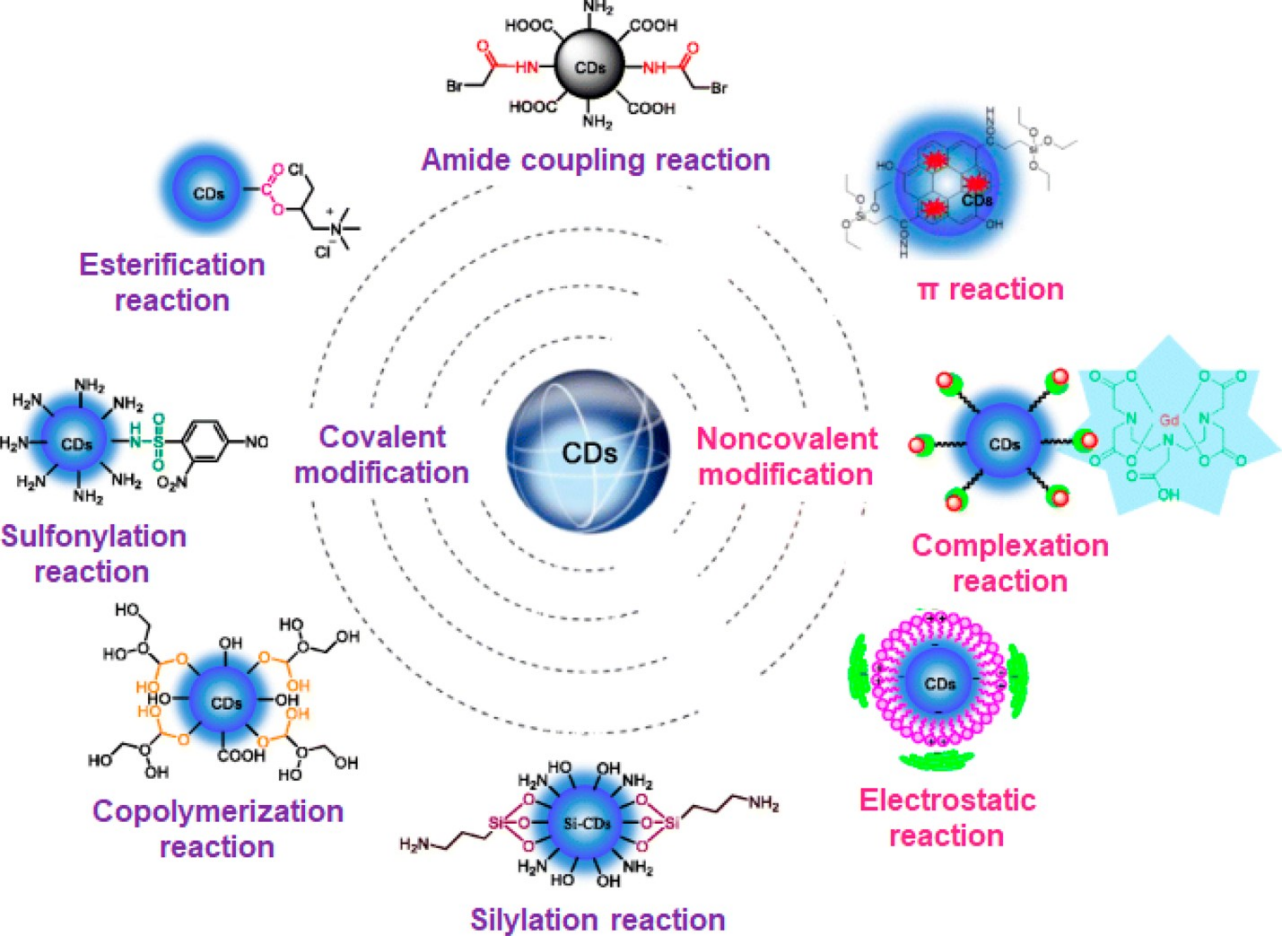
Schematic illustration of chemical binding strategies to decorate CDs with target specific ligands. Carboxy, hydroxy, and amino are the main surface groups on CDs, which can bind to other molecules through covalent and noncovalent reactions. Figure reprinted with permission from ref (101). Copyright 2018 Springer-Verlag GmbH Austria, part of Springer Nature.

Although there are clearly exciting prospects for the use of CDs for super-resolution imaging, several limitations remain and need to be resolved before their widespread adoption for biological research. First, the emission bands of the most frequently used CDs all appear in the blue to green spectral regions. The availability of efficient CDs emitting in the red and infrared spectral regions would be highly desirable for super-resolution imaging. The lower energy photons required for their excitation would reduce the generation of nonspecific autofluorescence from the sample, leading to improved signal-to-noise ratios, improved sample penetration and image contrast. Another problem is the very broad excitation bandwidth of CDs, which makes multiplexed applications difficult, i.e. where one desires to differentiate multiple fluorophores from the same sample simultaneously. Third, because the mechanisms causing photoblinking and photoswitching in CDs are not fully understood, a rational design of CDs with optimized properties for SRM imaging remains difficult. Finally, the practical exploitation of CDs for super-resolution imaging is still in its infant phase, and most applications so far have been proof of concept in nature. All examples reported of super-resolution imaging with CDs were performed using unspecific labeling with nonderivatized CDs in biological samples. Future work must focus on the design of CDs that are functionalized to reach specific subcellular targets. Chemical strategies to functionalize CDs are shown in Figure 8 and include both covalent and noncovalent surface modification. There is great opportunity here for material scientists and chemists to collaborate on the development of novel CD-based probes.

### 3.2 Quantum Dots

**Figure 9:**
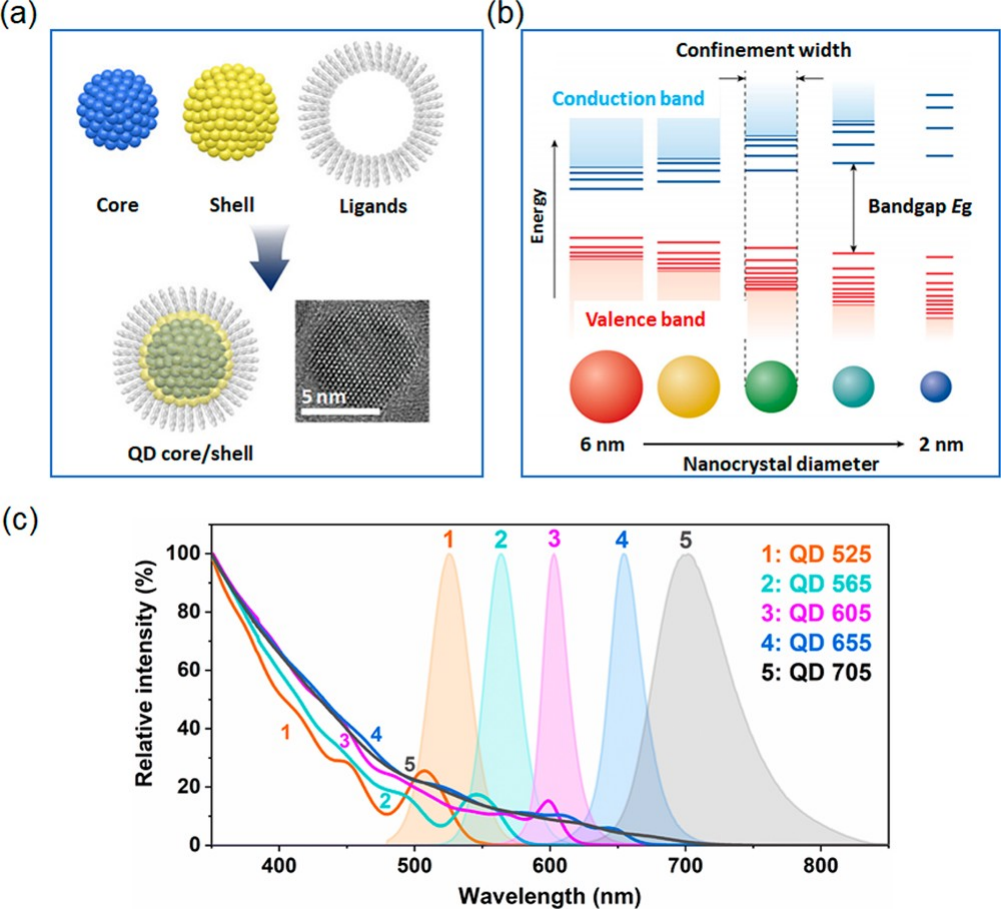
(a) Illustration of the structure of individual QDs and transmission electron microscopy (TEM) image of a CdSe/ZnS QD. Adapted with permission from ref (118) and SAMSUNG DISPLAY Web site (https://pid.samsungdisplay.com/en/learning-center/white-papers/guide-to-understanding-quantum-dot-displays). Copyright 2011 American Chemical Society. (b) Schematic illustration of the quantum confinement effect in QDs: with decreasing particle size, quantum confinement and hence the bandgap increase, leading to progressive blue shifts in the particles’ PL profiles. Adapted with permission from refs (119 and 120). Copyright 2017 Springer International Publishing AG; Copyright 2011 Royal Society of Chemistry. (c) Absorption and PL emission profiles for commercial CdSe/ZnS QDs conjugated with streptavidin from Thermo Fisher Scientific Co., Ltd. Individual QDs are designated according to their maximum emission wavelength, ranging from 525 to 705 nm, respectively. All QDs are identical in material makeup but differ in size and their emission spectra are independent of their excitation wavelength.

Quantum dots, QDs, have been extensively studied and were among the earliest inorganic probes designed for fluorescent bioimaging.^121^ Henglein at al. pioneered the synthesis of aqueous QDs in 1982.^122^ Since then, the field has seen intense development and numerous routes to the production of functionalized QDs have been researched. A single QD typically contains hundreds or thousands of atoms of group II–VI and IV–VI elements, for example, CdTe, CdSe, CdS, ZnS, ZnSe, PbSe, PbS, PbTe, and so on.^123^ QDs are semiconductor nanocrystals and are typically constructed to feature a core–shell structure measuring 2–10 nm in size with the atoms in crystal lattice arrangements (Figure 9a).^118^ The band gap in QDs is tunable via the size of QDs, and the energy difference between the highest valence band and the lowest conduction band increase as the QDs decrease through increased quantum confinement (Figure 9b).^119,120^ Thus, more energy is needed for excitation and more energy will be released as well (blue shift). Size alteration permits the emission spectra of QDs to be tuned easily all the way from the ultraviolet to the infrared spectral regions and hence the quantum confinement of electrons. The photoluminescence properties are severely affected by the surface properties of QDs and processes such as Auger recombination lead to nonemissive transfer of excited state energy, that can be avoided through a passivation of the core surface with a shell material. For practical purposes, QDs are therefore always constructed with a surrounding shell material. The shell helps in the confinement of excitons within the core and a reduction of surface-related recombination in trap states. The effect is an increase in the fluorescence quantum yield and but also protection from chemical degradation, e.g., oxidation and improving solubility.^121,124^ QDs possess attractive photophysical properties, such as outstanding photostability and a high fluorescence brightness. For example, QDs have been shown to be more than 100 times more photostable than Rhodamine 6G, with an almost 20-fold increase in brightness compared to the dye.^125^ These properties are superior even to AlexaFluor 488, one of the most efficient organic dyes available today.^126^ Furthermore, QDs feature a very broad excitation spectrum while their fluorescence emission is sharply confined to a narrow band of wavelengths (<50 nm) (Figure 9c).^127^ Photoblinking can be strong in systems where excited carriers can escape from the core to the QD surface and is thus strongly dependent on the shell thickness and type.^128^ The shell permits the conjugation of surface ligands to confer various physiochemical properties on the QDs and functionalize them for biological applications.^129−131^ Overall, there is good potential for the use of QDs for multicolor imaging and super-resolution imaging applications.

**Figure 10:**
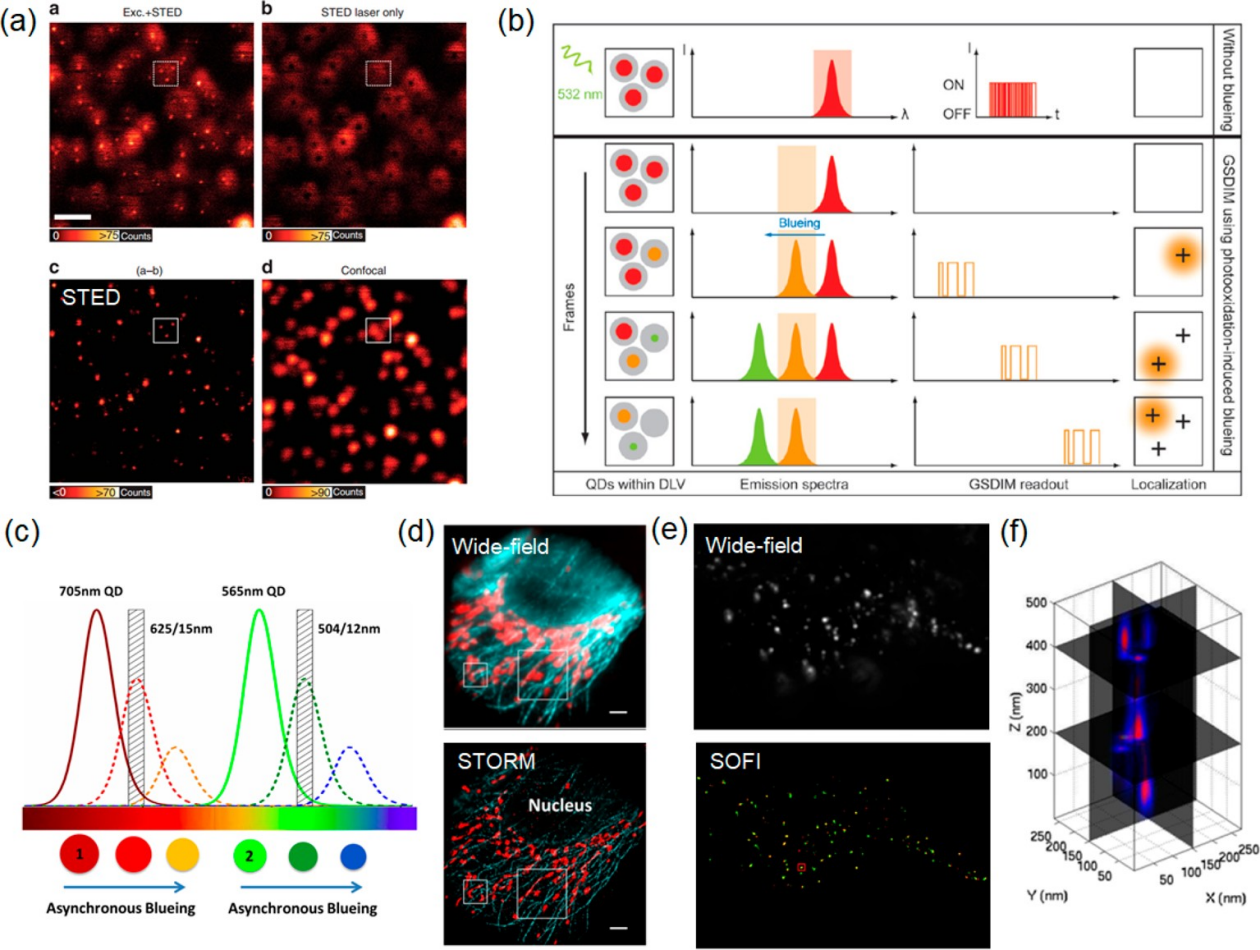
(a) STED imaging of fibers of vimentin, a structural protein, immunolabeled with QDs in fibroblast cells. Top left: excitation laser (405 nm) and STED depletion beam (592 nm) turned on simultaneously. Because of the very broad excitation spectra of QDs, there is considerable excitation of QDs by the STED beam, which appears as a halo in the periphery of the excitation laser. Top right: By switching on the depletion laser only, a background image is obtained, which can be subtracted from the STED images, resulting in improved resolution and contrast. Bottom left: STED image after background subtraction. Bottom right: Confocal image of the same region. Scale bar: 1 μm. Reproduced with permission from ref (132). Copyright 2015 Springer Nature. (b) Schematic illustration of the “blueing” phenomenon observed in QDs used for *d*STORM imaging. Three red QDs are presented within the diffraction limited volume (DLV). Without “blueing”, the blinking trajectories are not differentiable to distinguish individual emitters. Upon continuous irradiation at 19 kW cm^–2^ at 532 nm, the spatial density of blinking emitters reduces, and the trajectories of individual emitters become distinguishable, as the emission peak of individual detectors shifts toward shorter wavelengths. Reprinted with permission from ref (133). Copyright 2010 American Chemical Society. (c) Principle of multicolor imaging using “blueing” for two types of QDs. Continuous illumination and photooxidation causes a shrinkage in the QD size, and the resulting electron confinement leads to the associated shifts of QD spectra toward shorter wavelengths. In the illustration, the 705 nm QDs are seen to shift into the 625 nm passband while the 565 nm QDs transit the 504 nm passband. Both fluorescence signals can be recorded simultaneously without cross-talk. (d) Wide-field and two-color STORM images of QD 565 stained microtubules (blue) and QD 705 stained mitochondria (red) in HepG2 cells. Scale bar: 500 nm. Panels c and d were reprinted with permission from ref (134). Copyright 2015 American Chemical Society. (e) Demonstration of wide-field and three-dimensional SOFI imaging of epidermal growth factor receptors (EGFP) labeled with QDs. (f) 3D intensity profiles for QD aggregates located in the area indicated by the red rectangle in (e). Panels e and f were adapted with permission from ref (135). Copyright 2013 American Chemical Society.

For STED imaging, it seems that the high photostability of QDs makes them promising candidates. However, a bottleneck is their relatively small Stokes shift and that the broad excitation spectra generally extend into their emission spectra (Figure 9c).^136^ This increases the probability for fluorescence re-excitation by the STED beam, which must be avoided for a successful application of the technique. There is also a probability of re-excitation by two-photon absorption of light from the powerful STED laser. These factors have so far limited the potential of QDs in STED imaging applications and efforts have been directed at synthesizing QDs for which fluorescence re-excitation is minimized. The first STED application of QDs was carried out with commercial ZnS-coated CdSe and CdTe QDs (QD705).^132^ Even though the STED beam at 775 nm was well separated from the peak of the QD excitation spectrum (the intensity of which falls off rapidly beyond 700 nm), re-excitation at high depletion powers remained substantial. To deal with this problem, Hell et al. subtracted the resulting background from the STED images by recording STED images first with both the 628 nm excitation and the 775 nm STED beams switched on simultaneously,^132^ and then collecting an image of the background with only the STED beam turned on. Images recorded with the latter were subsequently subtracted to produce data featuring a lateral resolution of 54 nm on point-like emitters (Figure 10a). The technique was subsequently used to image structural fibers in fibroblast cells, and a resolution of 106 nm was achieved for the visualization of the QD-stained vimentin fibers. The high photostability enabled the same QDs to be repeatedly imaged over more than 1000 frames. Although the background subtraction method is straightforward to implement, it may not always a viable option. In another study, STED was demonstrated using the commercially available QD 705.^136^ To avoid the problem of two-photon-induced re-excitation, the authors used a continuous-wave depletion laser at 775 nm, instead of a pulsed picosecond laser source that is more conventionally used for STED. The lowered peak yielded an effective reduction in the 2-photon excitation at the STED wavelength. The authors were able to demonstrate a lateral resolution of 85 nm for the visualization of the microtubule network in HeLa cells.^136^ More recently, Qu et al. evaluated several types of commercial CdSe@ZnS QDs as potential STED probes,^137^ and found that green emissive CdSe@ZnS QDs (QD526) under 488 nm excitation could not be re-excited by a 592 nm continuous wave, cw, depletion laser (39.6 mW). On single quantum dots, they measured lateral STED profiles with a width of 21 nm.

The blinking-photophysics of QDs was first described by Nirmal et al. in 1996,^138^ who reported an intermittency in the fluorescence emission from single CdSe NPs subjected to cw excitation light. The current thinking is that photoblinking in QDs is caused by illumination-induced charging of the particle in the excited state and this in turn leads to a transition from the photoactive on-state into a charged, photoinactive off-state. Charge reneutralization then returns the QDs into their photoactive on-state.^110,139,140^ Repeated cycling of these processes causes the blinking observed in QDs. This provides potential for use of QDs for single-molecule localization microscopies. (Strictly speaking, single particle localization would be a more precise term in the context of CDs and QDs, because one is not dealing with individual molecules, but conceptually the methods are identical.) In pioneering work, a statistical analysis using independent component analysis (ICA) of the blinking characteristics was performed to separate individual and closely positioned QDs from one another.^141^ For STORM imaging, a low on-to-off duty cycle is a desirable feature. However, blinking rates are fast in QDs compared to other fluorophores in use for STORM. This increases the chance of simultaneous blinking of multiple particles within the emission PSF, thus negating an ability to discriminate between them.^47^ To overcome this issue, the so-called “blueing” phenomenon observed in QDs has been exploited.^142−144^ The effect is thought to be caused by a shrinkage of QD cores during illumination and is related to photo-oxidation. For example, in CdSe QDs selenium atoms can be photo-oxidized, which produces an evaporating SeO_2_ surface film, causing QDs to shrink over time,^145^ with a concomitant shift in their PL emission spectra toward shorter wavelength (“blueing”). In CdSe/ZnS QDs a blue shift of 29 nm was thus observed upon illumination at 570 nm with a 20 kW cm^–2^ laser beam until the particles eventually photobleached.^142^ Higher excitation intensities accelerate the “blueing” process.^146,133^Figure 10b demonstrates how the blueing phenomenon can be exploited to achieve optical super-resolution. By selecting a narrow spectral detection window, all QDs are initially indetectable. In time, individual QDs stochastically shift to shorter wavelengths and become detectable (on-state). As the detected QD blueshifts further, it passes the detection window and is thus “switched off”. As spectra of individual QDs transition through the detection window at different times, this permits a discrimination of overlapping diffraction patterns from single QDs. In practice, densely labeled biological structures have been visualized in this way at ∼25 nm resolution.^133^ The approach was later expanded to permit the simultaneous STORM imaging of QD565 and QD705 labels (Figure 10c).^134^ Here the wavelength shifts were observed on two different channels simultaneously, enabling 2 color super-resolution imaging. A drawback of “blueing” is that the QD brightness diminishes; however, sufficient photon numbers can usually be retrieved nevertheless, before bleaching occurs. Photon outputs as high as 3000 photons per localization were achieved by QDs, which is comparable to the best available photoswitchable dyes for SMLM. Figure 10d compares wide-field and STORM images of QD 565 stained microtubules (blue) and QD 705 stained mitochondria (red) in HepG2 cells. Resolutions of 24 and 37 nm were achieved in the lateral and axial dimensions, respectively, with STORM.^134^ Another method made use of hybrid blinking systems, consisting of QDs and surface-oriented crystal violet (CV) dye molecules,^147^ for the realization of single photoactivation/emission cycles: upon absorption of visible light, photoexcited electrons are transferred from the QDs to the CV dyes and this leads to emission quenching in the QDs. Further illumination fragments the CV dyes to a photoproduct that can no longer accept electrons. The result is the activation of the QD fluorescence, leading to emission of a photon burst. Further illumination causes CV darkening and QD-CVs can thus be photomodulated to emit a single high intensity photon burst during an activation-darkening cycle and used in localization microscopies. Their potential was demonstrated via introduction into HeLa cells, with photoblinking rates increasing almost 10-fold compared to nonmodified QDs under excitation with visible light. The strategy was used for the successful localization of multiple colors simultaneously.^147^ A conceptually similar hybrid system was synthesized using CdSSe/ZnS QDs as donor and A647 as acceptor molecules.^148^ Compared to the use of QDs or A647 individually, the hybrid system again exhibited improved blinking behavior. For optimal conditions a localization precision of 30 nm was reported for the hybrid reporters with PALM/STORM. The method was also demonstrated in live MRC-5 cells.^148^

Compared with STORM, SOFI allows a higher density of on-state fluorophores to reside within an area defined by of a PSF. The strong blinking exhibited by QDs is desirable for SOFI. It was shown that SOFI imaging of QDs deposited on a coverslip resulted in a 5-fold resolution gain compared to conventional wide-field microscopy.^27^ An enhanced contrast and a reduced background were also seen when QD-labeled microtubules were imaged with SOFI in fibroblast cells.^27^ An interesting way to obtain super-resolution, combines aspects of STORM and SOFI imaging and was reported by Shi et al.^149^ The authors developed tandem constructs containing one QD at each end, separated at a distance of ca. 6 nm. The two QDs where differentiable by emission color. By dispersing the fluorescence from the construct through a transmission grating, the fluctuation statistics of each QD could be spectrally differentiated and those periods recognized where one of the two QDs was in the off state. This method permitted an unambiguous assignation of the zero order signals to one of the two QDs and thus permitted very high photon numbers to be collected for their localization. The authors thus obtained more precise localization data than would have been obtained by either using STORM or SOFI alone.^149^ The measured distances were found to be consistent with the expected values of about 6 nm. This approach was also adapted for intracellular super-resolution imaging.

In another work,^150^ the joint-tagging SOFI (JT-SOFI) method was developed for imaging with ultrahigh labeling densities, enabled by the simultaneous use of three types of color differentiable QDs (QD525, QD625, QD705), again for imaging of the fine structure of microtubules, here in COS7 cells. JT-SOFI was found to perform better than SOFI preserve structural information in the image data. In addition, the labeling density for JT-SOFI can be increased 3-fold over that permissible for PALM/STORM imaging. Super-resolution imaging in 3D has also been performed with QDs, with reported resolutions of 8 to 17 nm in the lateral and 58 to 81 nm in the axial directions, using techniques that are conceptually identical to other SMLM methods (Figure 10e,f).^135^ The methods were used to resolve the 3D distribution of epidermal growth factor receptor (EGFR) molecules located on, or inside of, the plasma membrane of breast cancer cells.

**Figure 11:**
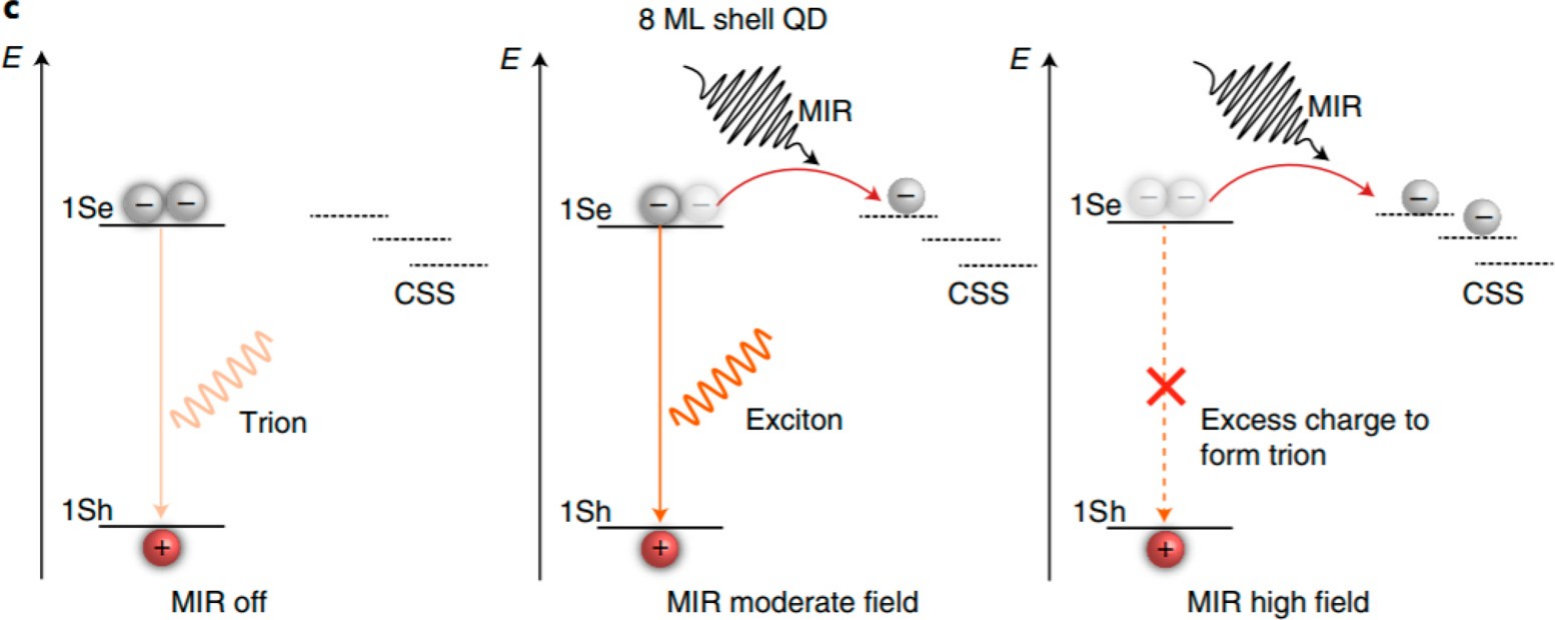
Illustration of the use of mid-infrared (MIR) light pulses to control the blinking characteristics of CdSe/CdS QDs. The core of the QDs is surrounded by a shell of 8 monolayers (MLs). When the MIR radiation is off, excess charge leads to trion formation with poor emission quantum yield and blinking behavior. The short MIR pulse at moderate field intensity can remove the excess charge on the surface and transfer it to trap states on the shell surface or the surrounding environment. A neutral exciton is restored, from which emission proceeds. At high fields, the exciton itself becomes ionized, resulting in an additional charge inside the dot. This gives rise to the formation of another trion and subsequent nonradiative Auger decay. Reprinted with the permission from ref (151). Copyright 2021 Springer Nature.

In efforts to increase the temporal resolution of SOFI, strategies were developed to produce QDs with tunable blinking characteristics. For example, QDs with thinner ZnS shells feature accelerated blinking rates, generating potential for live-cell SOFI applications.^152^ In another effort, the thickness of the ZnS shell of CdSe-ZnS core–shell QDs was varied systematically and the resulting changes in the blinking properties were analyzed.^153^ Certain ligands grafted onto the surface of QDs were found to reduce blinking rates.^154^ Similar reductions were obtained in alloyed core–shell interface systems or QDs possessing thick shells,^140,155−157^ or by contacting QDs with noble metal NPs.^158−161^ It is widely accepted that the blinking behavior of QDs is caused by two mechanisms.^110,162^ The first Auger recombination, a nonradiative process in which excited state energy is transferred to charged QDs nearby,^163^ with the result of photoluminescence quenching.^139^ The second is via activation and deactivation of trap states on the QD surface. The QD shell^164,165^ acts as a tunneling barrier and thus limits carrier escape to the surface, suppressing blinking. The thicker the shell, the stronger effect. For example, when QDs were coated with a seven-monolayer-thick ZnS shell,^138^ they were found to spend significantly more time in the “on” states, compared to bare QDs. However, despite progress in suppressing surface traps, the problem of Auger recombination remains. Some progress has been made by softening the structure of QDs and thus avoiding interface discontinuities.^166−169^ The effect is a lowering of spatial frequency components in the wave function which results in a partial suppression of the Auger process in charged NPs.^157^ In recent work, ultrafast mid-infrared (MIR) pulses (5.5 μm, 150 fs) with an appropriately selected field strength were applied to remove the excess electron from the trion-mediated Auger recombination in off-states of single core–shell CdSe/CdS QDs (Figure 11).^151^ The method led to a significant reduction in QD intensity flicker, and blinking could be almost eliminated in QDs encapsulated with thin (8 monolayers) shells. In summary, the blinking behavior of QDs is strongly affected by QD structure,^170−172^ shell thickness,^146−148^ the presence of trap states,^173^ surface ligands,^174,175^ and the external environment in which the QDs reside.^176−180^ An ability to adjust the blinking behavior of QDs, especially to decrease the on to off duty cycle of QDs, will be a breakthrough for SMLM imaging.

QD labels have also been demonstrated for SIM imaging. For example, QD605 was used to record both the distribution and the density of integrin αvβ3 receptors on single acute myeloid leukemia cells.^181^ A computer-based topological reconstruction of the QD distribution on the cell surface suggested a lateral resolution of ∼100 nm and axial resolution of ∼300 nm.^181^ Similarly, 3D SIM imaging was used to image QD-labeled CD13 protein on the surfaces of single cells revealing the distribution of individual proteins on the cell membrane.^182^ QDs have also been used for multiplexed SIM imaging in multiple colors. Using only a single excitation wavelength, QDs with different emission spectra could recorded simultaneously and differentiated by use of a color selective image splitter. Raw SIM images could thus be acquired simultaneously for each spectral channel, increasing acquisition speed.^183^

**Figure 12:**
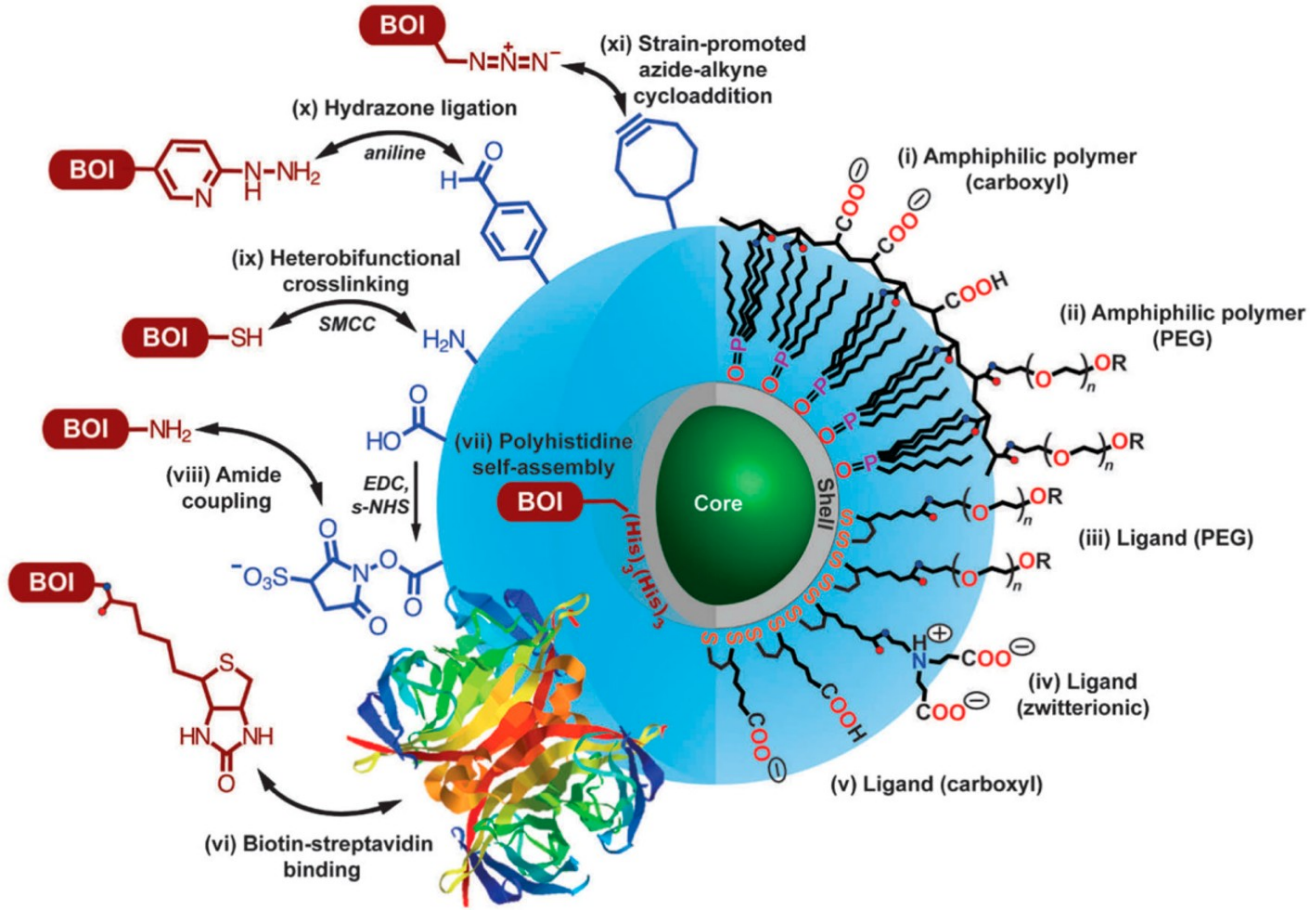
Overview of different surface functionalization strategies for QDs. The right-hand side shows typical polymers and ligands used for producing QDs with different chemical groups on their surface. The left side displays bioconjugation pathways for linking QDs with biomolecules of interest (BOI). Reprinted with permission from ref (184). Copyright 2013 Optica Publishing Group.

Despite of their favorable optical properties, such as superior photostability and brightness, some drawbacks prevail for the use of QDs in super-resolution imaging. For example, the broad absorption band and potential multiphoton absorption of QDs make them difficult to use for STED, similar to the problem discussed for CDs; blinking remains a major limitation; and the tendency of QDs to feature short off-state times compromises their use for single-molecule imaging. These considerations motivate the design of improved QD systems, e.g., via new synthesis routes, surface passivation strategies, and the construction of hybrid systems to control photophysical properties, e.g., to develop nonblinking QDs or QDs with controllable photon emission states for super-resolution imaging.^185,186^ Also, the photodynamics of QDs are different from standard fluorophores used in super-resolution imaging, and data analysis needs to be adapted to make optimal use of these systems in high-resolution imaging. A problem with QDs for biological imaging is that materials used for their synthesis are usually toxic for cell samples because they contain heavy metals like cadmiums. For example, exposure to UV light or oxidation in air can lead to leakage of free cadmium ions from CdSe QDs, which can cause cell death.^187^ For group II–VI QDs, it was furthermore demonstrated that exposure to light causes reactive oxygen species to form, which can also affect cellular function adversely.^188^ Different strategies are therefore required to surface treat QDs to make them biocompatible and functionalize them for specific applications.^189,190^Figure 12 summarizes different surface coating and bioconjugation strategies available for QDs.^184^ Although similar chemistries are available to functionalize QDs and CDs, an additional step is required to render QDs water-soluble. Usually, amphiphilic polymers or hydrophilic ligands are added for this purpose. Although bioconjugation chemistry is versatile for QDs and CDs, their size of both types of NP are very large compared to organic dyes and biomolecules, which might lead to ineffective recognition and function in cellular systems. There is still much room to explore efficient and multifunctional QD/CD-based bioconjugates. On the basis of the unique photophysical features of these NPs, it is furthermore conceivable to make environmentally sensitive probes for cellular environments, e.g., to measure cellular pH, ionic strength, or molecular interactions. Finally, although QDs have been intensively used in conventional imaging and biomedical applications, their use in super-resolution imaging is still in its infancy.

### 3.3 Polymer Dots

**Figure 13:**
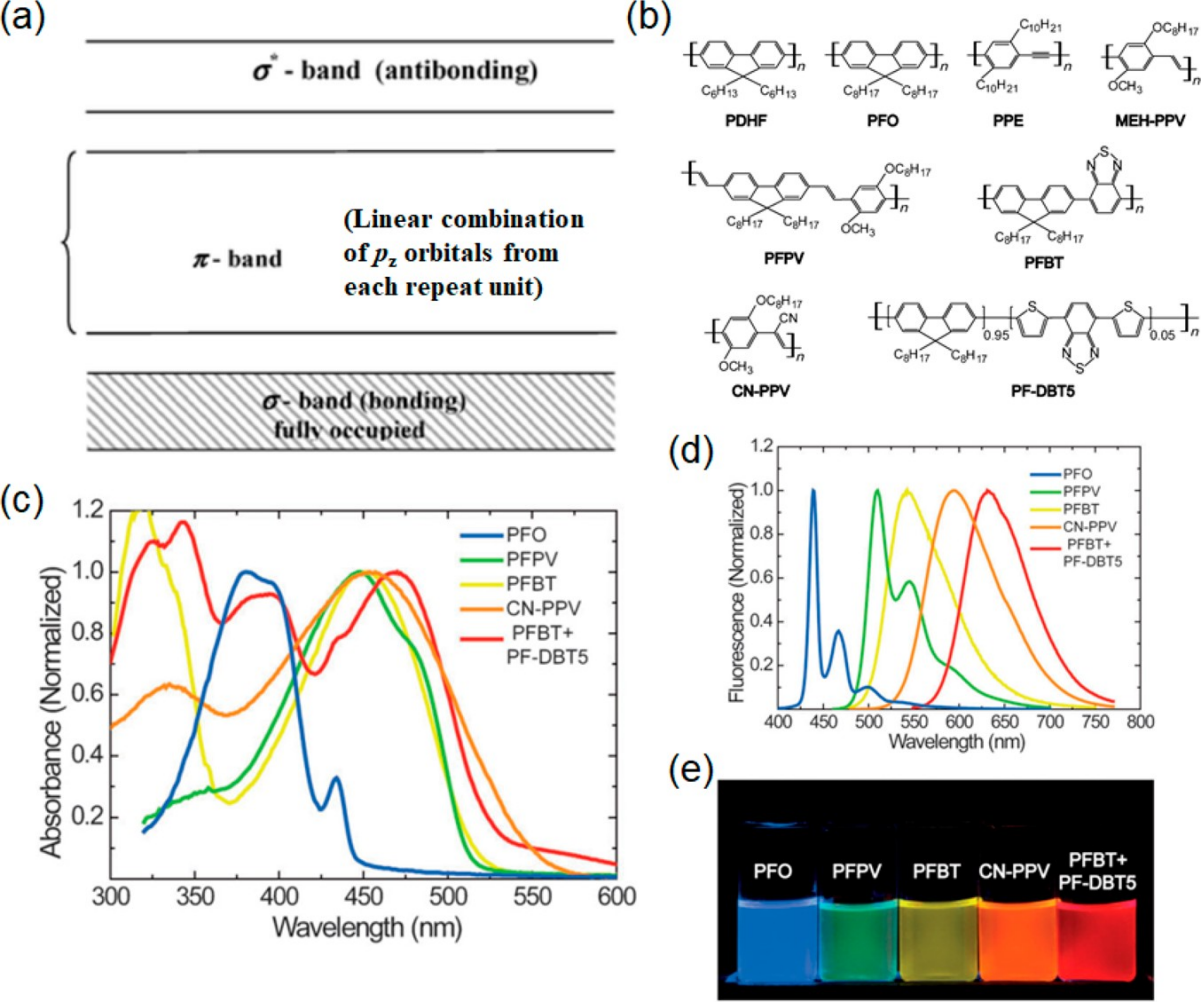
(a) Schematic illustration of band diagram of π-conjugated polymer. Reprinted from ref (191). Copyright 2010 Royal Society of Chemistry. (b) Chemical structures for different fluorescent semiconducting polymers. Adapted with permission from ref (192). Copyright 2013 WILEY-VCH Verlag GmbH & Co. KGaA. (c) Absorption spectra of PDs. (d) Fluorescence emission spectra of different kinds of PDs. (e) Digital photograph of PDs under UV light illumiination. Panels b–e were reprinted with the permission from refs (192−195). Copyright 2013 WILEY-VCH Verlag GmbH & Co. KGaA. Copyright 2008 American Chemical Society. Copyright 2012 Royal Society of Chemistry. Copyright 2011 WILEY-VCH Verlag GmbH & Co. KGaA.

In recent years, PDs have emerged as an attractive class of fluorescence probes. PDs are usually produced through the embedding of photoexcitable structures in a suitable polymer matrix that is usually hydrophobic and occupies a volume or weight fraction of around 50%. The diameters of PDs range from 20 to 30 nm, although smaller sizes have also been reported.^192^ PDs are NPs formed from π-conjugated polymers, dyed doped polymers, or fluorescent polymers, usually by emulsion polymerization of nanoprecipitation. The backbone of conjugated polymers features an array of light-harvesting units, for example, alternating σ- and π-bonds. Band structures for such systems are shown in Figure 13a,^191^ where it is seen that σ-bonds bind the structure together, while π-bonds lead to semiconducting behavior. Typical chemical structures for fluorescent semiconducting polymers are shown in Figure 13b.^192^ Generally speaking, these materials feature a direct band gap that can be tuned through modification of the molecular structure of the polymer.^191,196,197^ Their absorption bands range from 350 to 600 nm and a multitude of emission bands are available across the visible spectrum (Figure 13c–e).^192−195^ Compared to molecular dyes, polymers loaded with fluorescent dyes feature a higher brightness and photostability since they comprise a large number of fluorophores per particle which are protected by their embedding matrix.^198^ PDs are approximately 3 orders of magnitude brighter than conventional organic fluorescent dyes.^199,200^ Compositional changes affect their photoluminescence behavior and can be used to design systems that can be optimized for either continuous fluorescence or photoblinking behavior.^201,202^ It is postulated that electron hole polarons quench PD fluorescence and this to give rise to photoblinking.^203,204^ The tunable optical features, together with the versatility available in polymer design, make PDs promising candidates for super-resolution imaging.

**Figure 14:**
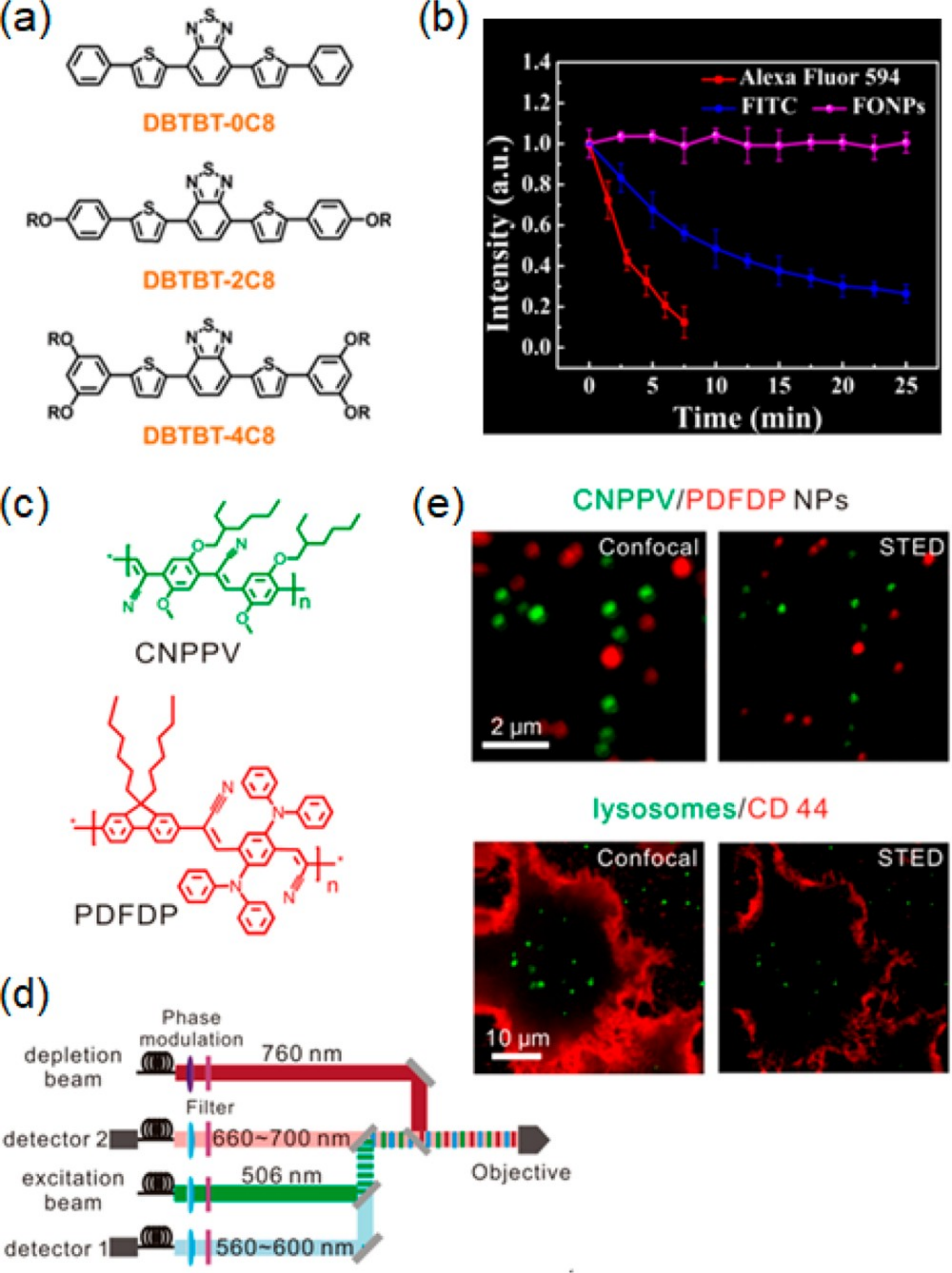
(a) Chemical structures of three types of fluorescent organic nanoparticles (FONPs). (b) Fluorescence intensity plots of DBTBT-4C8 contained FONPs, FITC, and Alexa Fluor 594 in HeLa cells under 600 mW STED laser irradiation. Panels a and b were reprinted with the permission from ref (205). Copyright 2019 American Chemical Society. (c) Chemical structures of CNPPV PDs and PDFDP PDs. (d) Simplified illustration of dual-color STED microscopy. (e) Top: Confocal and STORM images of mixture of CNPPV PDs and PDFDP PDs. Bottom: Confocal and STORM images of PDFDP PDs labeled lysosomes and CD44 antibody-PDFDP labeled lysosomes. Panels c–e were reprinted with the permission from ref (206). Copyright 2020 American Chemical Society.

Deep-red fluorescent organic nanoparticles (FONPs) were developed as shown in Figure 14a.^205^ Because of their high brightness (PLQY 25%) and good photostability, these FONPs were successfully employed in STED for HeLa cells and glass catfish imaging, with an improved resolution of ca. 100 nm. The photostability of FONPs was compared with those of commercial FITC and Alexa Fluor 594 fluorophores (Figure 14b). After 25 min of STED laser (600 mW) irradiation, FITC and Alexa Fluor 594 were almost photobleached, while FONPs remained unchanged. In addition, FONPs showed much improved resolution in STED imaging compared to FITC and Alexa Fluor 594 fluorophores. In a more recent work, two kinds of semiconducting PDs with different emission wavelengths were prepared for dual-color STED imaging and cellular tracking (Figure 14c).^206^ Some PDs exhibit very large Stokes shifts. The Stokes shift is ca. 149 nm for CNPPV and ca. 260 nm for PDFDP, respectively, and both are excitable with a 506 nm laser. They are depleted by a 760 nm laser beam and collected in separate channels (Figure 14d). The concept was exploited to study the dynamic interaction of clathrin-derived endosomes and caveolin-1-positive endosomes, which were tracked in HeLa cells, with a resolution down to 68 nm (Figure 14e).

For single-molecule localization imaging, photoswitchable PDs have been designed. One strategy here is to make use of Förster resonance energy transfer (FRET) in PDs in which donor and acceptor molecules are incorporated. An example of this is a PD system in which photochromic spiropyran molecules are conjugated to PFBT. Under UV irradiation, spiropyran is converted into its merocyanine form, which absorbs visible light and acts as a FRET quencher for PFBT fluorescence, with an efficiency exceeding 85%. On the other hand, excitation with visible light causes the recovery of the PFBT fluorescence. The system exhibits good reversibility over many cycles and a large modulation difference between the on and off states. These advantages are complemented with excellent brightness and a small particle size (∼16 nm). The system is readily functionalized for biological imaging and conjugation with streptavidin permitted the specific labeling of microtubules and membranes in live MCF-7 cells.

Because PDs are large compared to typical Förster radii, FRET can be inefficient when PDs are loaded with donors and acceptors because their molecular proximity may not be readily achieved. One interesting approach to address this problem exploits the use of a process called exciton diffusion of FRET donors.^207^ Bulky hydrophobic counterions were employed to prevent self-quenching of the donor and to facilitate the diffusion of excitons between octadecyl rhodamine B dyes within a poly(d,l-lactide-*co*-glycolide) matrix. The process greatly increased the probability of an exciton meeting an acceptor site within the PD volume. FRET deexcitation rates were increased as a result and thus photoswitching efficiency. In another case, PFBT was doped with the fullerene derivative PCBM to form PDs of ca. ∼14 nm in diameter. The PDs feature a fluctuating steady-state population of hole polarons, leading to time variable quenching and thus photoblinking. PDs thus modified display intense bursts of 3–5 × 10^4^ photons during each switching event, with brightness levels that are 1–2 orders of magnitude greater than those of conventional photoswitchable dyes. A remarkable localization precision of ∼0.6 nm has been demonstrated for these systems, an improvement of approximately 4 times over dye molecules. The topology of PD-labeled *Escherichia coli* bacteria could be mapped out precisely with SMLM and in cells a localization of ∼5 nm was achieved.^208^ In more recent work, the authors controlled the charge carrier generation and recombination dynamics in semiconducting PDs, resulting in a 3–8-fold improvement in localization precision compared to dyes and fluorescent proteins.^209^

**Figure 15:**
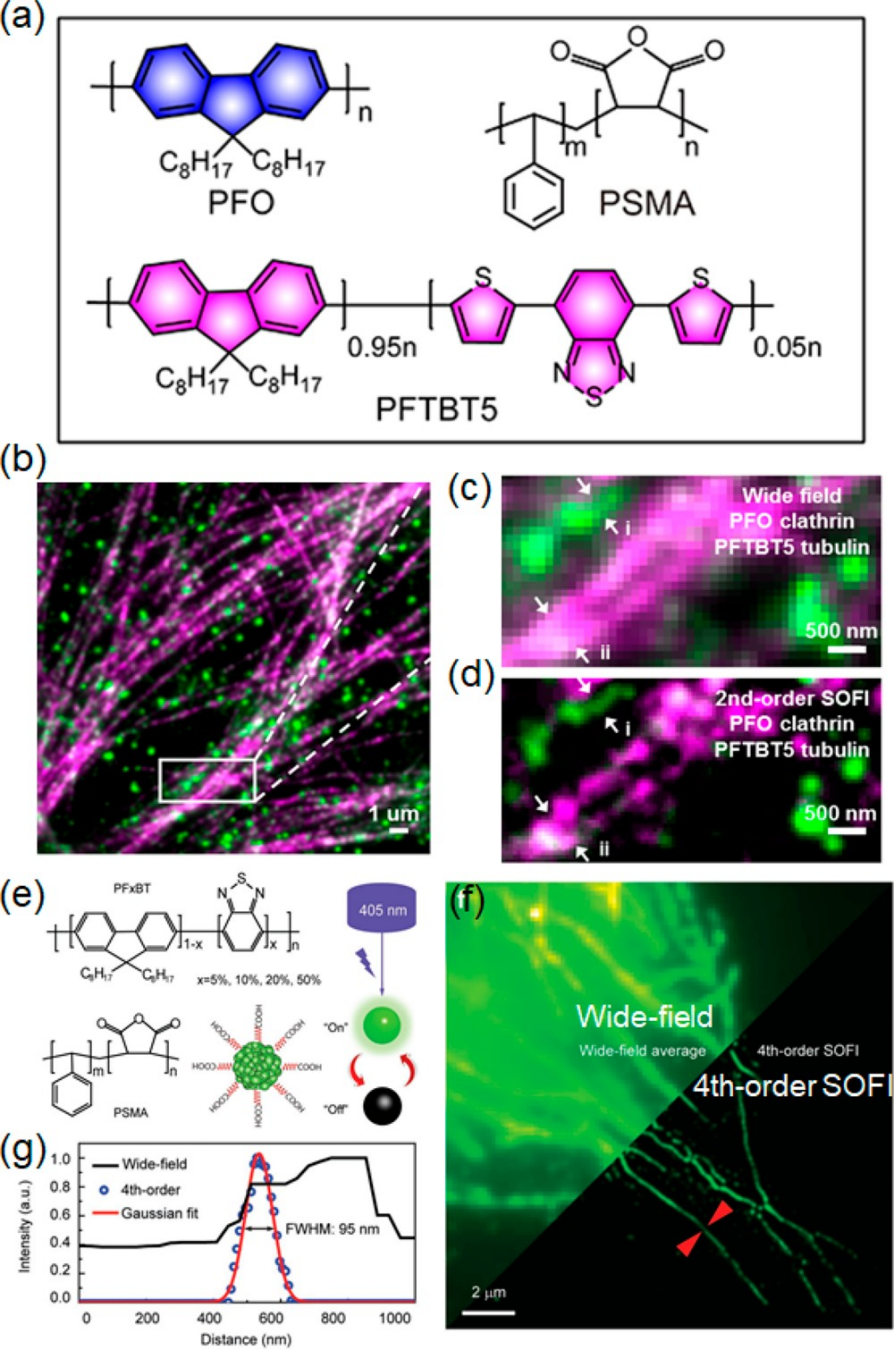
(a) Chemical structures of PFO, PSMA, and PFTBT5. (b) Wide-field imaging of clathrin coated pits labeled with PFO PDs and microtubule labeled with PFTBT5 PDs. (c) Magnified region in the white box of panel b. (d) Second-order SOFI image of the same region with panel c. Panels a–d were reproduced with permission from ref (210). Copyright 2017 American Chemical Society. (e) Chemical structure of polymer PFxBT and functional polymer PSMA. (f) Wide-field image and fourth-order balanced SOFI image of microtubules labeled with PF10BT PDs. (g) Fluorescent line profiles of the yellow arrows shown in panel f before and after fourth-order SOFI imaging. Panels e–g were reproduced with permission from ref (211). Copyright 2019 WILEY-VCH Verlag GmbH & Co. KGaA.

The fluorescence kinetics of PDs appear to depend on particle size. For example, for PDs over 15 nm in diameter, a continuous fluorescence emission was observed with no significant photoblinking. However, below 10 nm, the same material exhibits strong intermittency in fluorescence emission.^192,193,212^ Photoblinking in small PQs was first demonstrated in semiconducting polymer PFBT and CN-PPV.^213^ Similar to other NPs, such as CDs and QDs, the emission statistics of these PDs also obey a power law distribution, indicating that a small number of emitters with reversible on/off dynamics induce fluorescence fluctuations, while only a small portion of PDs are in the on-state.^192^ The PD system offers high brightness, good photostability, and excellent biocompatibility. Functionalized PDs work well as biological labels. Streptavidin-conjugated PFBT and CN-PPV have been used to label and visualize mitochondrial membranes, nuclear pores, and microtubules in BS-C-1 cells. SOFI experiments have furthermore been performed offering a resolution of features in the cell down to ∼180 nm. SOFI with multiple colors has also been achieved with PDs,^210^ through combined use of blue emitting PFO PDs (particle size: 10 nm) and red PFTBT5 PDs (particle size: 13 nm) (Figure 15a). Both feature narrow fluorescence emission bands, permitting easy discrimination. Compared with Alexa Fluor 405 and QDs 655, PFO and PFTBT5 showed a 4.3-fold and 2.4-fold improvement in brightness, respectively. Excellent performance of these systems was demonstrated in BS-C-1 cells. Using streptavidin-conjugated PDs and dual-color SOFI, clathrin-coated vesicles and microtubule filaments were resolved in these cells, yielding resolution improvements by a factor of nearly 1.7 over standard widefield imaging (Figure 15b–d). In another work, a series of semiconducting PDs was prepared from PFxBT and PSMA by a nanoprecipitation method (Figure 15e).^211^ Despite their relatively large size (∼20 nm) compared to the former system, they offer greater flexibility in adjusting photoblinking properties, which can be simply controlled via the number of chromophores per particle. PF10BT PDs were thus used to resolve microtubule structures by high-order SOFI microscopy with excellent resolution and contrast. Figure 15f compares the wide-field and fourth-order SOFI images with the latter offering a 4-fold resolution improvement (from 400 to 95 nm) (Figure 15g). PALM imaging with a dual-color PD system is also possible with blue and orange fluorescent PDs. For the system to work, it is essential that energy transfer between two types of PD is effectively suppressed, here mediated by excited-state intramolecular proton transfer. This is a prerequisite to enable an effective switching between the emissive and nonemissive states and good modulation contrast between these two states. PALM images of PD-labeled RAW264.7 cells revealed features down to 70 nm in size.^214^ PDs thus enrich to the family of photoblinking labels available for biological super-resolution imaging.

**Figure 16:**
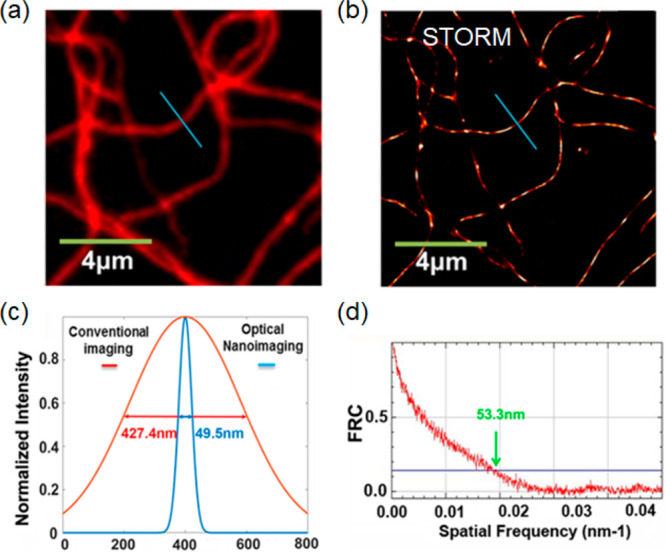
(a) Conventional fluorescence image of cylindrical micelles formed from PSt-*b*-PEO staining by DSA-2SP. (b) STORM imaging of the same region in panel a. (c) Cross-sectional intensity profiles of a cylindrical micelles. (d) Fourier ring correlation (FRC) curve produced from panel b. Panels a–d were reproduced with permission from ref (215). Copyright 2017 American Chemical Society.

**Figure 17:**
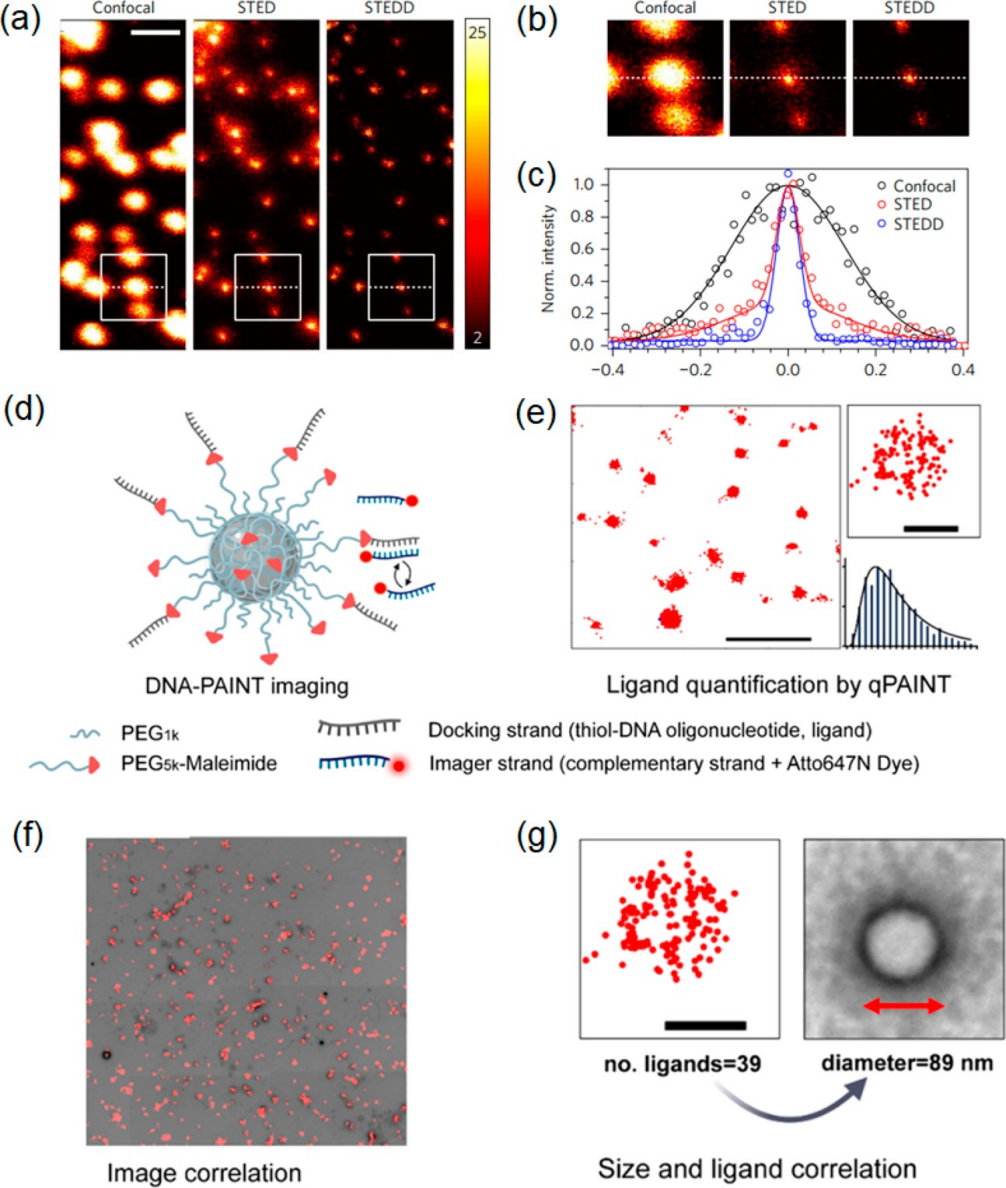
(a) Images of 40 nm PS beads recorded using confocal, STED, and STEDD microscopies. Color bar, counts per pixel; scale bar, 1 μm. (b) Enlarged regions from the square in panel a. (c) Intensity profiles along the dashed lines in panel a. Panels a–c were reproduced with permission from ref (217). Copyright 2017, Nature Publishing Group. (d) Schematic illustration of ligands on PLGA-PEG NPs. (e) Localization coordinates of ligands on PLGA-PEG NPs by quantitative PAINT (qPAINT) analysis. (f) Correlated PAINT and TEM images of PLGA-PEG NPs. (g) Correlated size and quantified ligand number of single PLGA-PEG NP. Panels d–g were adapted with permission from ref (218). Copyright 2021 American Chemical Society.

**Figure 18:**
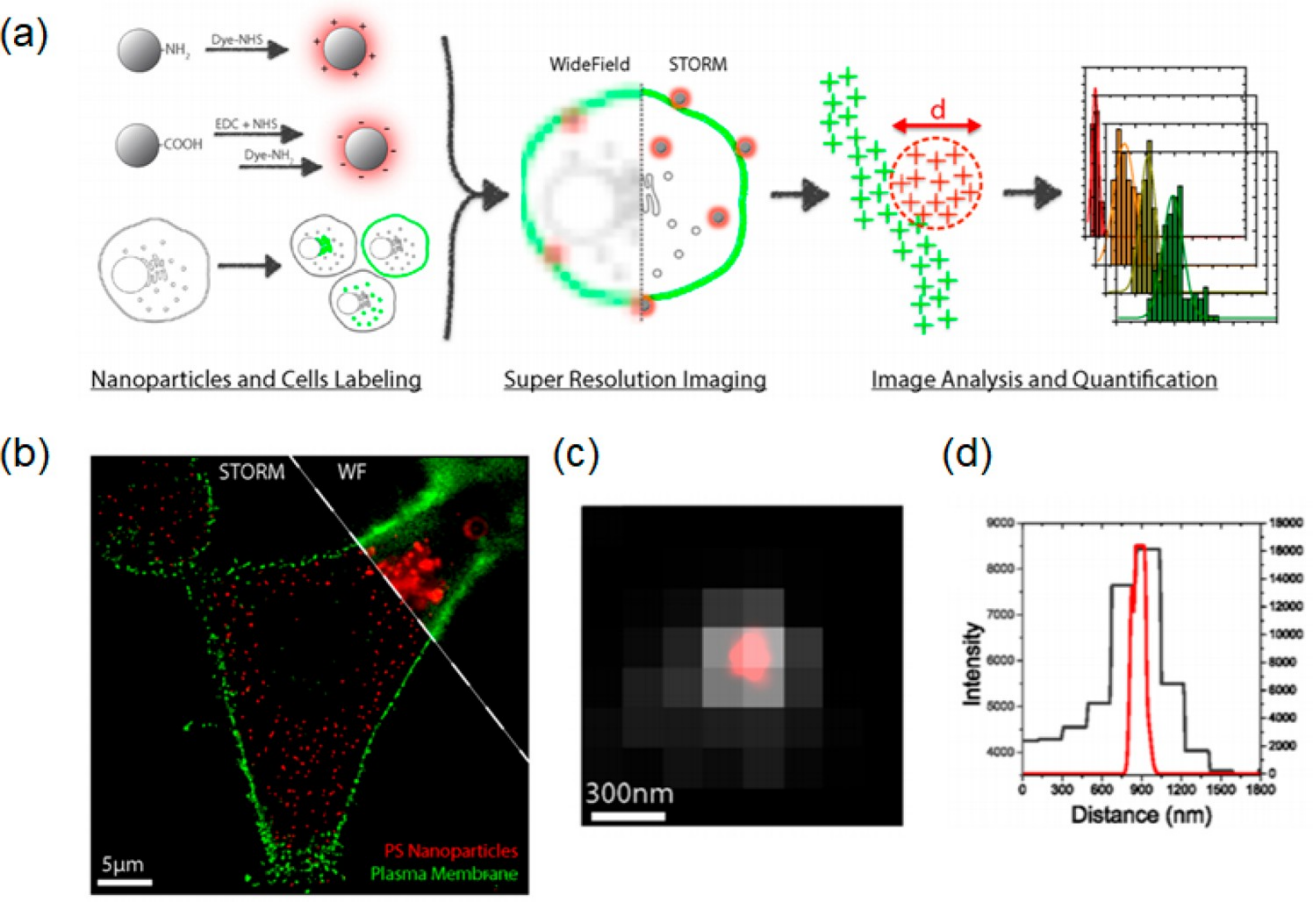
(a) Workflow for PS nanoparticle functionalization and cell labeling, STORM imaging and data analysis. (b) Comparison of STORM and wide-field image of 80 nm PS NPs inside membrane-stained HeLa cells. (c) Overlay of wide-field (gray) and STORM (red) images of a single NP. (d) Comparison of intensity profile in wide-field and STORM images from panel c. Panels a–d were reproduced with permission from ref (220). Copyright 2016 American Chemical Society.

Dye-doped PDs also possess favorable optical properties. Common synthetic fluorescent dyes emit between 10^4^ and 10^6^ photons in aqueous solution before being permanently photobleached. The embedding of dyes in rigid matrices can lead to significant suppression of this undesirable effect and improve brightness. In one example, Alexa Fluor 647-conjugated peptide PDs were used for whole mouse imaging.^216^ Mouse tumor tissue subsequently imaged *in vivo* and *ex vivo* with STORM. In another work a novel photochromic compound (a spiropyran-functionalized distyrylanthracene derivative, DSA-2SP)^215^ was synthesized and applied for super-resolution imaging. The compound was subsequently dispersed in the diblock copolymer of polystryrene-*block*-poly(ethylene oxide) (PSt-bPEO) to form cylindrical micelles. By switching between UV and visible illumination light, the fluorescence behavior of these micelles could be cycled reversibly thus permitting STORM imaging (Figure 16a–d). Other dye-doped NPs, for example, dye-doped polystyrene (PS) NPs with various surface modifications also have potential for various imaging applications. The 40 nm PS NPs have become useful tools for the performance evaluation of advanced super-resolution imaging techniques. One example is shown in Figure 17a–c where the resolution of stimulated emission double depletion microscopy (STEDD) is measured compared to standard confocal microscopy.^217^ STEDD is able to suppress background signal through use of a second STED pulse. For biological application, functional moieties on the PDs largely determine their cell targeting behavior. Using correlative PAINT and transmission electron microscopy (TEM) imaging, it was possible to quantify the number and density of Atto-647N labeled ligands on poly(lactic-*co*-glycolic acid) (PLGA)-polyethylene glycol (PEG) NPs (Figure 17d).^218^ With PAINT (Figure 17e), the number of surface ligands per NP was identified on particles localized via TEM (Figure 17f,g). This strategy offers promise to investigate NP structure–function relations. In another case, colloidally stable, carboxylate-functionalized PS NPs were used to visualize endocytosis in COS7 cells via PALM and signal levels were sufficient to acquire images with good spatial resolution.^219^ Two-color STORM was realized using either Cy5 or Alexa647 labeled PS NPs, using EDC/NHS chemistry for linkage (Figure 18a).^220^ The *d*STORM image shows that 80 nm PS NPs were internalized by HeLa cells (Figure 18b). A comparison between the STORM and wide-field images clearly demonstrates a substantial increase in resolution (Figure 18c,d).

PDs have also been deployed for STED imaging.^221^ Hydrophobic fluorescent PDFDP and amphiphilic PSMA were used to form monodisperse aqueous solutions of PDs. PDs measured around 40 nm in size, of which STED images yielded cross-sectional profiles of 71 nm. As stated earlier, PDs are easily quenched by the nonfluorescent hole polarons. However, it was found polarons are short-lived in PDs when the illumination cedes, and the system returns to a photoactive fluorescence state. For example, the fluorescence of PDs recovered completely after 2 min following a 1 min illumination period. Long-term STED imaging is thus possible, if the excited pixels are allowed to recover into a photoactive state during the raster scanning process. Thus, it was possible to image PDs inside cells continuously over 2 h. Even in live cells STED imaging was successfully demonstrated and biotin-conjugated PDs were imaged to track the movement of endocytic vesicles. The PDs used were proven to be more photostable than the organic dye Atto565, commonly used in STED.

Despite the outstanding photophysical properties exhibited by PDs, improvements are desirable: first, a capability to reduce particle sizes to below 10 nm would endow favorable fluorescence properties for super-resolution imaging, such as improved photoblinking and intermittency characteristics. Furthermore, a smaller size confers improved compatibility for biological systems and effectuates their use as functional probes. Second, designing PDs specially for STED imaging would enrich the arsenal of available labels for this important technique. Third, for stochastic super-resolution imaging techniques strategies need to be developed that permit the rational modulation of hole polarons in PDs to achieve optimal blinking behavior. Finally, there is potential to exploit PD systems in multiplexed imaging applications through design with multiple excitation and emission bands featuring minimal spectral overlap.

### 3.4 Modified Silica NPs

Dye-doped silica NPs have similar PL properties as PDs. The performance of dyes can be improved by coating them onto silica NPs and the silica substrate provides a versatile platform for linkage chemistry. Enhanced photophysical performance includes improved brightness and photoblinking.^222−226^ STED imaging has been successfully carried out using these dye-doped silica NPs. The internalization of Atto647N labeled silica NPs with the particle size around 25 or 85 nm in A549 cells was quantified through STED, with a resolution of ∼61 nm.^227^ Later, the same research group also embedded other dyes, such as Abberior STAR 635, Dy-647, Dy-648, and Dy-649, through covalent coupling onto the silica matrix by aminosilane chemistry. Compared with free uncoupled dyes, the hybrid systems show improved photostability (∼1.6-fold) and brightness (∼1.4-fold), enabling them to be used as cellular fluorescent probes in STED imaging, with reported resolutions of ∼85 nm.^228^ Taking Atto647N-transferrin NPs as another example,^229^ an approximately 4-fold resolution improvement has been achieved in STED imaging, compared to standard confocal imaging. The photostability of these Atto647N-transferrin NPs was found to be superior to that of the dye alone: under STED illumination (780 nm, 80 MHz pulses of 300 ps width, 28 mW), the fluorescence intensity of NPs declined to half of the original intensity at a rate ca. 1.6 times smaller than that of dye conjugates on their own. The transferrin-based protein NPs have application potential as drug carriers in clinical medicine through their cofunctionalization with active agents. STED was used to investigate the cellular uptake of these NPs and to locate their cellular fate. Interestingly, a large number of NPs were found to accumulate within individual endosomal vesicles. Quantitative comparisons of the enhanced potential of dye conjugation to silica NPs were also carried out for Cy3 and Cy5. The studies were performed using STORM imaging of these dyes conjugated to ultrasmall (ca. 6 nm) silica NPs.^230^ The system was used to image block copolymer nanostructures deposited in thin films on substrates. The dye encapsulated NP system exhibited an improvement in the photon budget (∼3.1-fold) and a decrease in the localization uncertainty (∼2-fold) compared to the free dyes. Similarly, Alexa Fluor 647 (AF647) labeled mesoporous silica NPs ca. 80 nm in size where used in HeLa cells, again showing much improved resolution over conventional confocal imaging.^231^ The presence of silica-coated magnetic NPs doped with rhodamine B isothiocyanate dye was quantified by STORM analysis in various cell lines,^232^ such as HEK 293, NIH3T3, and RAW 264.7 cells. The cell types exhibit different internalization behavior. For example, macrophage cells show more NPs uptake than human kidney and fibroblast cells, especially into the nuclear region. In another case, the ultrasmall (<10 nm) organic dye doped core–shell aluminosilicate NPs enabled live-cell STORM imaging to quantify the size of intracellular vesicles and the number of NPs per vesicle. These are encouraging results, especially in consideration of the fact that silica NPs are easily functionalized to carry active cargo. In future, there is thus scope for combined therapeutic and diagnostic applications using these systems.

### 3.5 Aggregation-Induced Emission Dots

**Figure 19:**
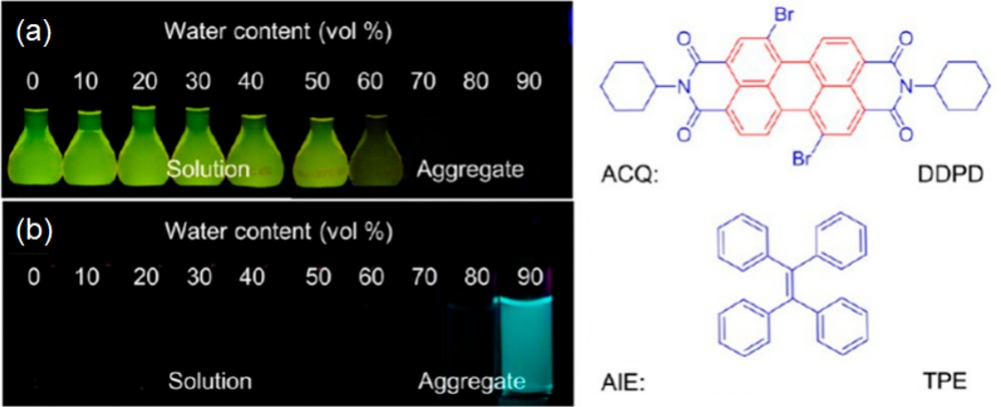
Fluorescent photographs (left-hand) and molecular structures (right-hand) of (a) DDPD with aggregation-caused quenching (ACQ) effect and (b) TPE with (aggregation induced emission) AIE effect in tetrahydrofuran/water solvents with different water volume fractions. Panels a and b were adapted with permission from ref (233). Copyright 2013 American Chemical Society.

Aggregation induced emission (AIE) has caught intense interest as a phenomenon to exploit for new fluorescence probes. AIE luminogens (AIEgens) are nonemissive, or weakly emitting, fluorescence systems when in solution, but become highly fluorescent in their solid state.^234−236^ The phenomenon was first discovered by Tang and co-workers in 2001.^237^ The AIE effect originates primarily from the restriction of intramolecular motion, more specifically, the suppression of rotational and vibrational degrees of freedom when AIEgens are in aggregate form.^238^ AIEgens are systems whose molecular structures mimic the shapes of “propellers” with each propeller arm constituting a molecular rotor. Rotor motion in the soluble state prevents the formation of large coplanar structures in which electrons can freely move and this leads to loss of fluorescence. In contrast, in the solid form, the structures are locked into place but twists in the conformation of individual units, prevent the formation π–π stacks, which can lead to nonradiative loss of excited state energy, often a reason for the fluorescence loss in traditional fluorescence systems upon aggregation.^233,239,240^ An example of aggregation-caused quenching (ACQ) in *N*,*N*-dicyclohexyl-1,7-dibromo-3,4,9,10-perylenetetracarboxylic diimide (DDPD) is shown in (Figure 19a). DDPD features strong π–π stacking interactions in its aggregated state, thus its fluorescence decreases upon addition of water, which induces aggregation. Tetraphenylethylene (TPE), on the other hand, represents a typical example of an AIEgen. It adopts a twisted propeller-shaped conformation (Figure 19b),^233^ which suppresses intermolecular π–π interactions when it forms aggregates on addition of water.^241^ These AIE NPs can be encapsulated to vary in size and surface functionality, and systems with low toxicity, good biocompatibility, and high resistance to photobleaching have been developed.

**Figure 20:**
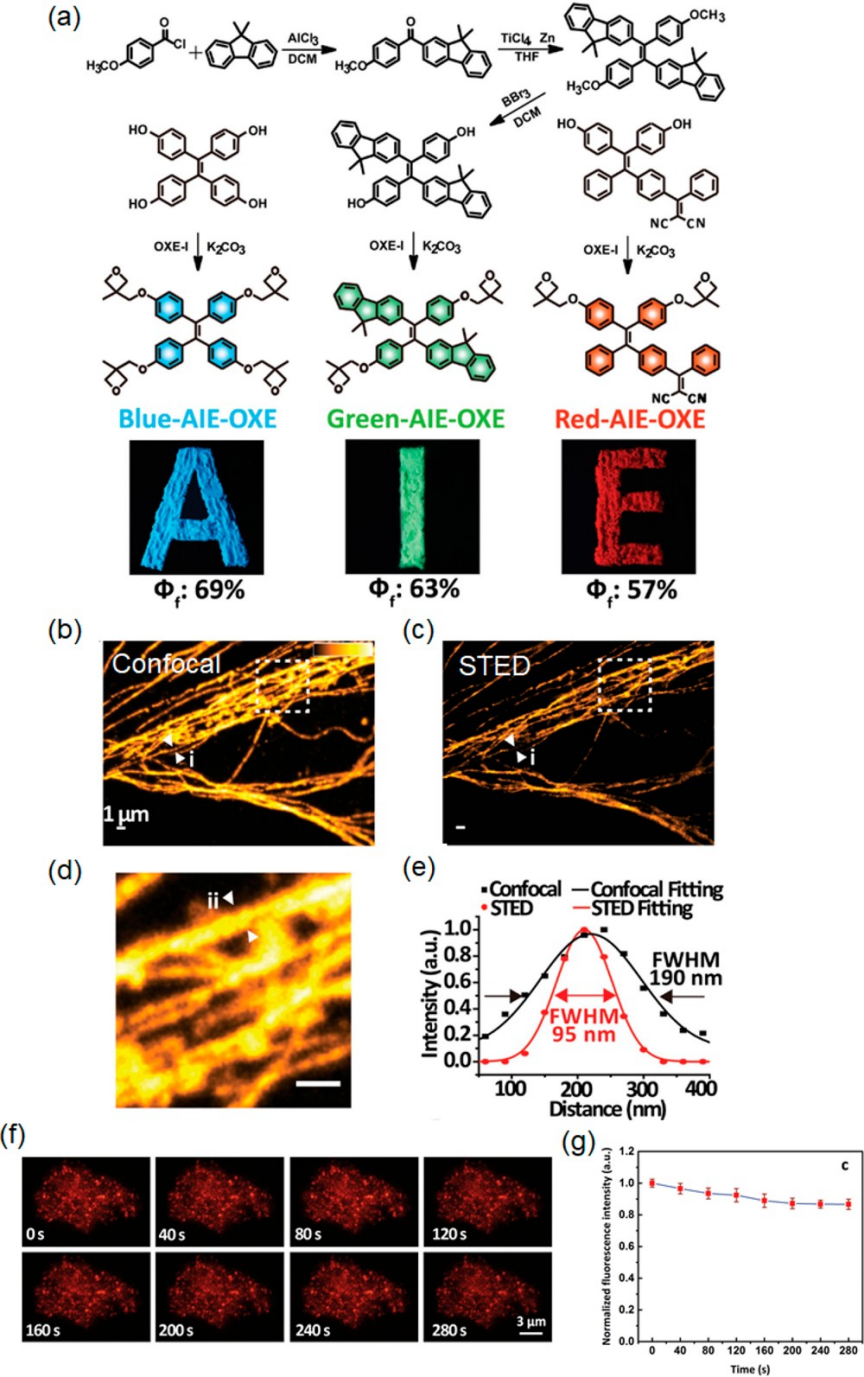
(a) Synthetic routes and structures of three types of AIE NPs. (b) Confocal and (c) STED images of microtubules labeled with red emissive AIE NPs. (d) Magnified views of region marked in panels b and c). (e) Intensity profiles across microtubule marked with “i” in panels b and c. Scale bar: 1 μm. Panels a–e were adapted with permission from ref (242). Copyright 2017 WILEY-VCH Verlag GmbH & Co. KGaA. (f) STED imaging of TTF@SiO_2_ over prolonged time points exhibits long-term stability. (g) Normalized fluorescence intensity of TTF@SiO_2_ collected at prolonged time points, during 280 s of continuous scanning under a 594 nm excitation beam and a 775 nm depletion beam. Panels f and g were adapted with permission from ref (243). Copyright 2017 WILEY-VCH Verlag GmbH & Co. KGaA.

**Figure 21:**
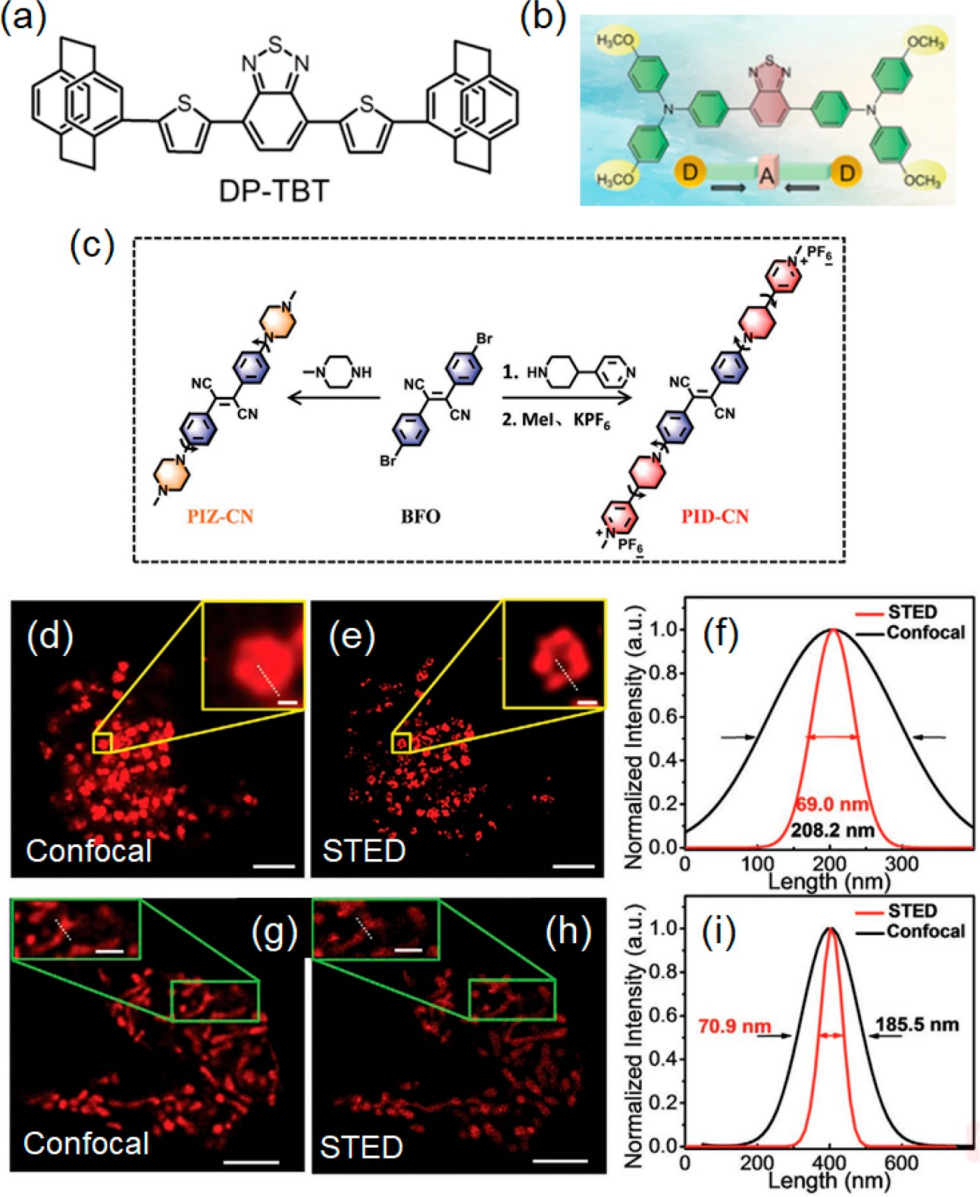
(a) Chemical structure of DP-TBT. Adapted with permission from ref (245). Copyright 2019 American Chemical Society. (b) Chemical structure of DTPA-BT-M. Adapted with permission from ref (246). Copyright 2016 Royal Society of Chemistry. (c) Chemical structure of PIZ-CN and PID-CN. Confocal (d) and STED (e) images of PIZ-CN labeled lysosomes in a live HepG2 cell. The large yellow regions are the enlarged forms of the small region in each image. The scale bars in panels d and e are 3 μm. The scale bars in the insets are 200 nm. (f) Fluorescence intensity profiles corresponding to the white dashed lines in panels d and e. Confocal (g) and STED (h) images of PIZ-CN labeled mitochondria in a live HepG2 cell. The large green regions are the enlarged forms of the small region in each image. The scale bars in panels g and h are 1 μm. The scale bars in the insets are 500 nm. (i) Fluorescence intensity profiles of the white dashed lines in panels g and h. Panels c–i were adapted with permission from ref (247). Copyright 2020 Wiley-VCH GmbH.

In pioneering work, three types of oxetane-substituted AIE (AIE-OXE) NPs with emission in the blue, green, and red spectral regions, respectively, were synthesized (Figure 20a).^242^ They feature small size (∼15 nm), high quantum yield (higher than 60%), and good colloidal stability. The large Stokes shift exhibited by red emissive AIE NPs permits their use as STED imaging probes. For example, microtubule structures in MCF7 cells could be imaged with STED using such a strategy (Figure 20b–e). The spatial resolution of 95 nm was achieved under relatively low illumination intensities (∼100 MW cm^–2^) for the STED beam, indicating reasonable performance for stimulated depletion, and therefore sample protection when using this technique.^242^ In one promising approach, 2,3-bis(4-(phenyl(4-(1,2,2-triphenylvinyl)phenyl)amino)phenyl) fumaronitrile (TTF) was encapsulated by colloidal mesoporous silica (TTF@SiO_2_), to produce red/near-infrared (NIR) fluorescent NPs.^243^ Using a 594 nm laser and a 775 nm laser as excitation and STED beams, respectively, the stimulated emission depletion efficiency of TTF@SiO_2_ was shown to be better than 60%, similar to some of the best conventional STED dyes, such as Atto647N.^244^ Moreover, TTF@SiO_2_ NPs feature large Stokes’ shifts (150 nm) and are resistant to photobleaching even under long-term (280 s) irradiation with a high-power STED beam (312.5 mW average power) (Figure 20f,g). This performance appears favorable over other frequently used STED dyes, such as Coumarin 102. STED imaging of HeLa cells labeled with TTF@SiO_2_ was demonstrated, with a lateral spatial resolution of 30 nm. Other AIE luminogens such as DP-TBT (Figure 21a) exhibited a PLQY of 25% and was successfully used for STED imaging of helical fibers.^245^ DTPA-BT-M (Figure 21b) is a related system with a measured PLQY of more than 30% and a large two-photon absorption cross section.^246^ This system can be applied in both STED and two-photon fluorescence (TPF) microscopies, with the former offering excellent resolution and the latter good penetration for deep tissue imaging. Using this combined approach, lipid droplets could be imaged with 95 nm resolution, while TPF was capable of imaging 300 μm deep in mouse derived lung tissue. Finally, two further very efficient AIE luminogens, PIZ-CN and PID-CN, have been reported to feature depletion efficiencies of up to 90%, at illumination intensities in the 1 to 5 MW cm^–2^ range (Figure 21c).^247^ These two probes were successfully used for live cell STED imaging of lysosomal fusion (Figure 21d,f) and mitochondrial fission (Figure 21g,i), providing a new strategy for revealing organelle interactions in cells at a high spatial resolution.

The photoswitching properties of AIE NPs have also been explored. Tang et al. reported a TPE derivative, named as *o*-TPE-ON+,^248^ which can undergo a photocyclodehydrogenation reaction under irradiation with visible light, turning the system from a dark into a photoactive state. This photoactivation is promoted by oxygen and the system appears to associate specifically with mitochondria. In fixed cells, mitochondria could be resolved with resolution of 104 nm by STORM imaging. Similarly, AIE-active diarylethenes (TPE-2DTE and OTPE-2DTE) were reported later,^249^ which are also photoactivatable. Their superior photoswitching behavior is beneficial for STORM imaging and was used to resolve cylindrical micelles down to 50 nm detail. In another study, AIEs were constructed through incorporation of two large steric units of benzothiophene, BBTE and the material deposited as a thin film on a microscope slide. This system featured outstanding AIE performance and could be reversibly switched between on- and off-states through alternating the irradiation between UV and visible light. The fwhm of the smallest resolved details measured 32 nm with this system. In summary, there is great potential for the construction of photoresponsive AIE NPs for super-resolution imaging.^250^

### 3.6 Nanodiamonds

**Figure 22:**
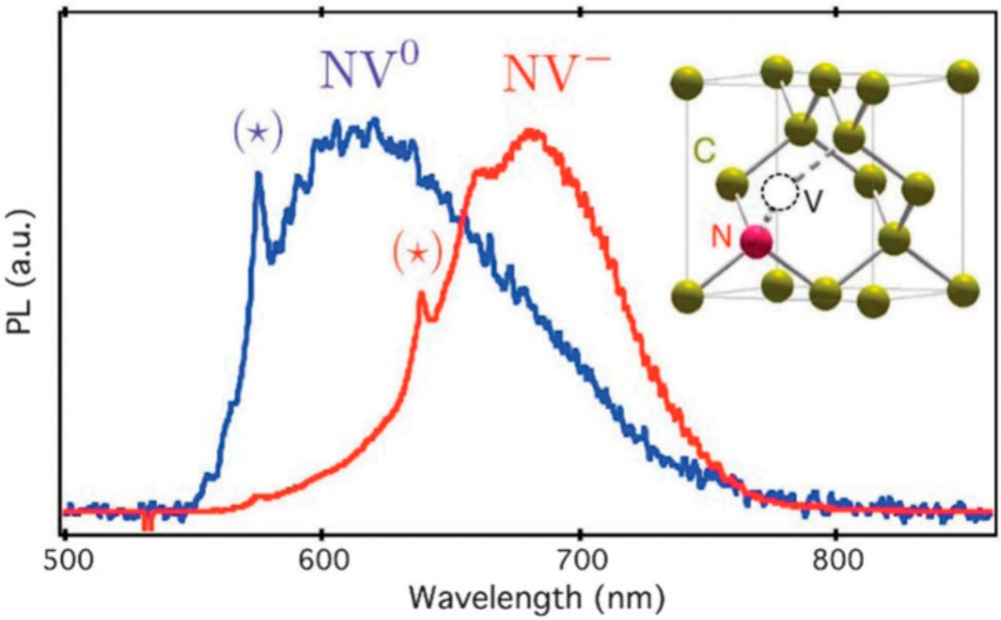
Normalized PL emission spectra of NV^–^ and NV^0^ centers in NDs. The zero-phonon lines (*) represent for NV^–^ (637 nm) and NV^0^ (575 nm). The inset shows the structure of NV centers in NDs, which involves a substitutional nitrogen atom (N) associated with a vacancy (V) in an adjacent lattice site of crystalline matrix. Reprinted with permission from ref (251). Copyright 2010 American Physical Society.

The emergence of fluorescent nanodiamonds, NDs, has opened up another potential avenue for imaging in biological research. The structure of NDs consists of carbon atoms in sp^3^-hybridized arrangements, and they are inherently biocompatible. Unlike many other NP systems, which can be synthesized through wet chemistry methods, NDs can only be produced by chemical vapor deposition, high-pressure and high-temperature method, and detonation of explosives.^252^ When defect free, NDs are inherently transparent because of the very large bandgap in diamond. However, (Figure 22) upon irradiation with He^+^ ions, protons or high energy electrons, charge vacancies are formed in NDs. Subsequent annealing above 700 °C results in the diffusion of these vacancies to the nitrogen atoms, thereby introducing the nitrogen-vacancy (NV) defect centers.^118,253−257^ The phenomenon causes NDs to become photoluminescent and visible excitation light generates a bright and stable long-wavelength emission. Specifically, two forms of NV centers exist in NPs: NV^0^ and NV^–^. Neutral NV^0^ normally exhibits fluorescence emission near 575 nm, and the negatively charged NV^–^ center exhibits emission near 637 nm (Figure 22) with a high fluorescence quantum yield under 532 nm excitation.^253,258^ Both electronic transitions can be coupled with phonons to exhibit emission side bands peaked at ∼700 nm.^259,260^ Besides the green and red fluorescence,^261−263^ NDs with blue emission peaked at 450 nm have also been synthesized.^264^ The fluorescence emission of NDs is exceptional stable, and no sign of photobleaching is observable even under high-power laser excitation, making NDs ideal for long-term imaging without signal decay. Another attractive aspect is the long fluorescence lifetime (∼20 ns) of NDs, which is much longer that of biological autofluorescence from biological tissue.^265^ Temporal gating during signal collection therefore permits an efficient discrimination of fluorescence signal from background autofluorescence and thus an effective enhancement of image contrast. Furthermore, the PLQYs of NDs is very high, ranging between 0.7 and 1, higher than almost any other fluorophore system in this wavelength range.^266^ Moreover, NDs can be surface functionalized to realize the specific labeling and targeting functions in cells.^267^ These features make NDs a fascinating alternative to organic dyes, fluorescent proteins, and QDs offering new potential for bioimaging applications.^253,261,268−275^

**Figure 23:**
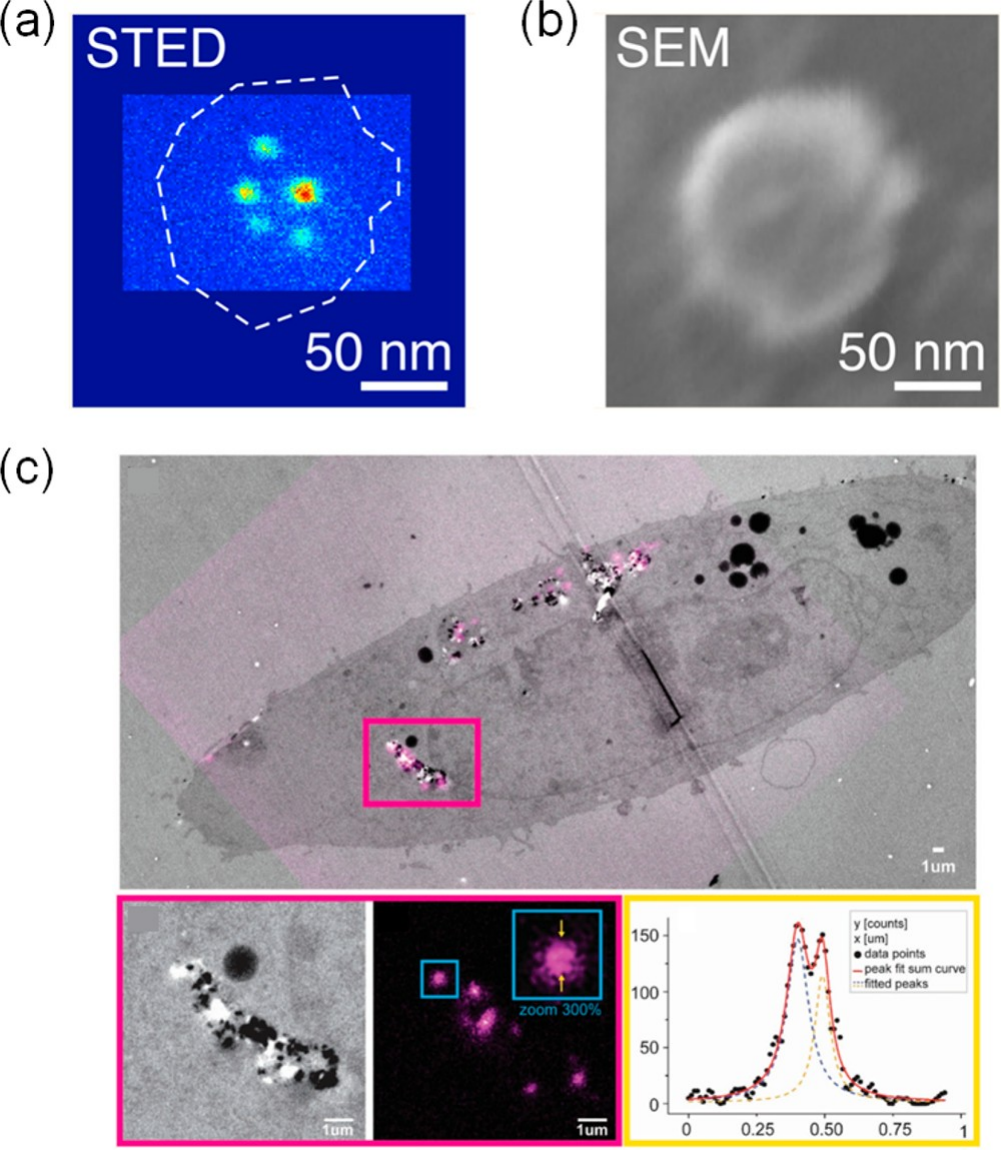
STED (a) and SEM (b) images a nanodiamond. Adapted with permission from ref (279). Copyright 2013 American Chemical Society. (c) STED-TEM correlative images of intracellular NDs in TEM sections. Upper: Overlay image of TEM (gray) and STED (fluorescence signal from NDs in magenta). Bottom (from left to right): Zoomed section of correlation result is shown for TEM and STED, respectively. The line profile values and a two-peak Lorentzian fit of the data. Reproduced with permission from ref (281). Copyright 2017 WILEY-VCH Verlag GmbH & Co. KGaA.

Their photostability and high quantum yields make NDs the ideal probe for STED imaging applications. The first example was demonstrated by Hell and co-workers, offering the best resolution ever reported with STED imaging and individual NV^–^ centers could be localized with a remarkable resolution of 5.8 nm.^276^ The authors were able to study the photophysical features, e.g., single-photon emission signature of these color centers and the aggregation and heterogeneity of the NPs using tunable laser sources for STED.^277^ A problem in the use of NDs as fluorescent probes in cells is particle agglomeration, which has been partially addressed via the conjugation of albumin to NDs, for which a homogeneous labeling of cells was achieved for STED imaging.^278^ The delivery of NDs into cells can be achieved via electroporation or by endocytosis and profiles of individual NDs within the cell revealed a resolution of ∼40 nm in HeLa cells. It was found that while cytoplasmic albumin coated NDs remained mostly homogeneously distributed in the cell, they were seen to aggregate in endosomes. Movement of these organelles could be dynamically tracked in the cells using STED. Improvements on this principle permitted individual NV centers to be imaged at 10 nm resolution, within NDs of various shapes, measuring 40–250 nm in size (Figure 23a,b).^279^ Both red and green emitting NDs have been produced for STED imaging, and this provides an opportunity for high-quality two-color STED imaging, something that remains difficult to achieve with alternative STED dyes.^280^ A very interesting opportunity arises through the use of NDs as dual-contrast probes in correlative STED and transmission electron microscopy (TEM) imaging.^281^ The two techniques subject the sample to vastly different environmental conditions and sample preparation protocols, yet no degradation of performance loss was evident even over long-term or repeated experiments, proving the system to be robust as dual-contrast probes in correlative STED-TEM microscopy of cells (Figure 23c).

The photoblinking behavior of NDs has also been studied and it was shown that the positioning of defects on the surface of NDs affects the photoblinking behavior.^282−285^ Etching of the ND host has been shown to lead to the appearance of an intermittency in the photoemission.^286^ Furthermore, in individual 5 nm NDs formed from detonation-synthesized diamond, photoblinking was also observable from nitrogen vacancies present in the material.^287^ This means single NV centers can be super-resolved with SMLM at a resolution of 20 nm or better.^288^ A statistical analysis of the photon emission suggests that there are multiple NV centers per ND particle, each acting as an intermittent photon source. In another published work, ground state depletion (GSD) was reported as a method to image NDs with optical super-resolution.^289,290^ Here, NV centers were put into a metastable dark state under continuous level illumination.

For practical applications, some limitations of NDs need to be addressed to enable efficient super-resolution imaging. A problem of NDs for use as biological reporters is their comparatively large size, and techniques for routinely producing NDs that are considerably smaller than 10 nm are highly desirable. So far, most imaging experiments have been carried out in bulk NDs contained in solution and hardly any attempts have been reported on their use in biological research, although there is potential for in vivo imaging: NDs feature deep red or NIR fluorescence and are usually compatible with biological function, ideal characteristics for deep tissue imaging with good sensitivity and resolution. For multiplexed imaging it is important to design NDs emitting in different wavelength bands. Finally, other photophysical properties of NDs, such as photoblinking and photoswitching need to be better understood to enable their rational design for improved SMLM modalities.

### 3.7 Upconversion NPs

**Figure 24:**
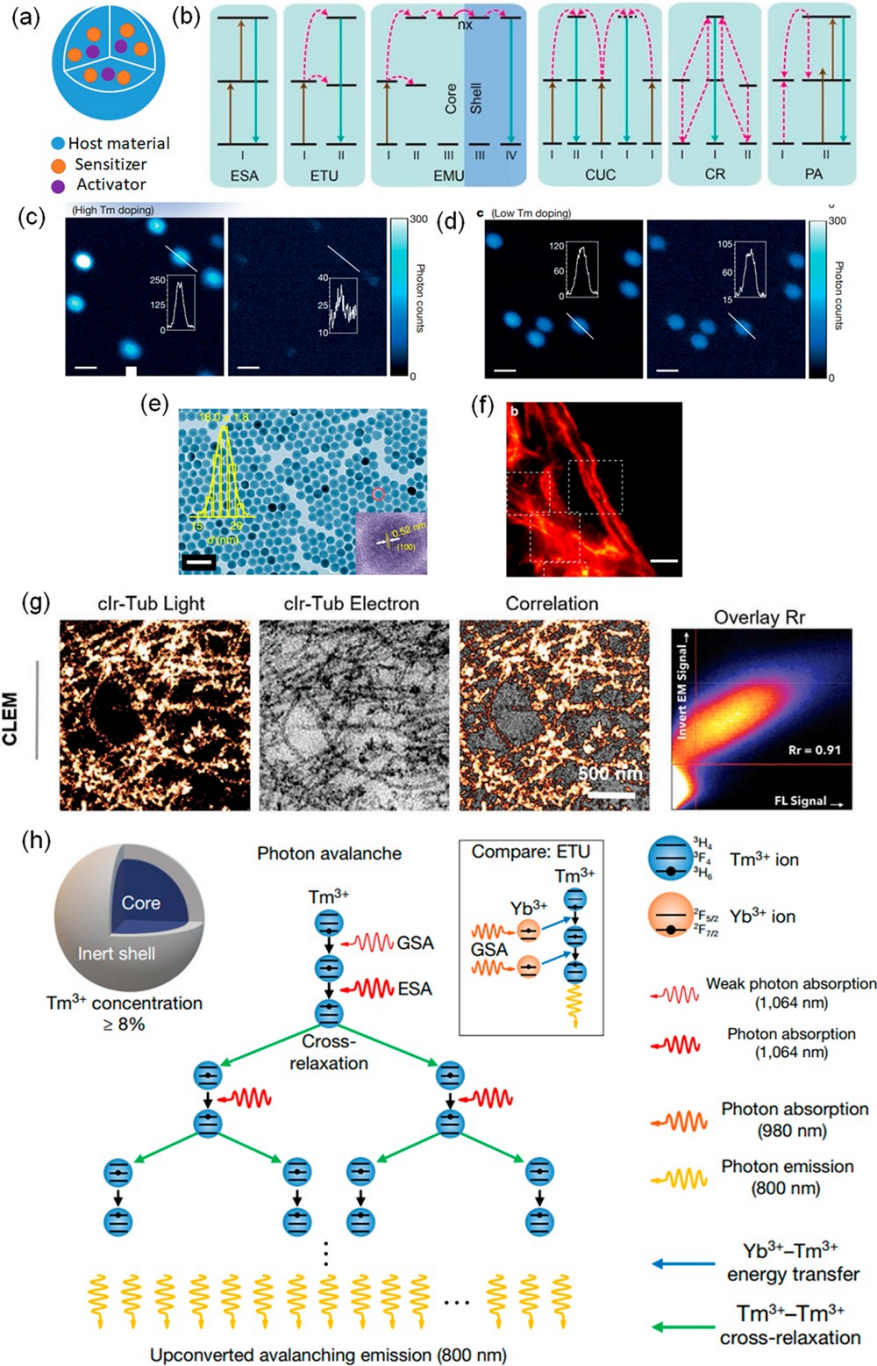
(a) Schematic illustration of UCNP structure. (b) Typical energy level diagrams of upconversion processes. From left to right: Excited-state absorption (ESA), energy transfer upconversion (ETU), energy migration-mediated upconversion (EMU), cooperative upconversion (CUC), cross-relaxation (CR), and photon avalanche (PA). Reprinted with permission from ref (319). Copyright 2019 Elsevier Ltd. (c) Confocal image of 8% Tm-doped UCNPs under continuous-wave 980 nm laser (left) and under both 980 and 808 nm laser (right) excitation, with upconversion emission at 455 nm. The power was 1 mW. (d) Imaging setup as in panel c with 1% Tm-doped UCNPs, but with laser power at 5 mW. Panels c and d were reprinted with the permission from ref (291). Copyright 2017 Macmillan Publishers Limited, part of Springer Nature. Insets: Fluorescence intensity profile from the diagonal white line in images. Scale bar: 500 nm. (e) TEM (large) and high-resolution TEM (small) images of NaYF_4_:18% Yb^3+^, 10% Tm^3+^ UCNPs. Insets: Distribution of particle sizes. (f) STED image of antibody-conjugated UCNPs labeled cellular cytoskeleton protein desmin under 975 nm excitation and 810 nm STED laser beam. Scale bar: 2 μm. Panels e and f were adapted with the permission from ref (292). Copyright 2017 Springer Nature. (g) Correlative STED and scanning-transmission electron microscopy images of cIr-Tub labeled microtubules in HepG2 cells, the colocalization scatter plot shows high correlation with Pearson’s coefficient, *R*_r_ = 0.91. Adapted with the permission from ref (293). Copyright 2020 Wiley-VCH GmbH. (h) Photon avalanche (PA) mechanism in Tm^3+^-doped UCNPs. The avalanching occurs in the core–shell UCNPs with core Tm^3+^ concentration over 8%. Inset shows the energy-transfer upconversion (ETU) process. GSA, ground state absorption; ESA, intense excited state absorption. Adapted with the permission from ref (294). Copyright 2021 Springer Nature.

Upconversion NPs, UCNPs, have been developed as a promising material for multiphoton probes.^295−303^ They feature nonlinear optical properties permitting the conversion of two or more photons into a higher energy photon. Low-energy NIR photons can thus be converted into higher energy NIR, visible, or even UV emission.^304^ The long-wavelength absorption of UCNPs features a high penetration depth and reduces autofluorescence from biological samples. Each UCNP is composed of thousands of codoped lanthanide or actinide ions embedded in a host lattice to form a network of photon sensitizers and activators (Figure 24a). The activator ions act mainly as luminescent centers, while the sensitizer ions absorb NIR light energy that is transferred to the activators to facilitate the emission. The most critical factor that affects upconversion luminescence efficiency is the cross section of the sensitizer ions for absorbing NIR radiation. Yb^3+^ or Nd^3+^ possess large absorption cross sections in the NIR and are therefore frequently used as sensitizers.^305,306^ Er^3+^ and Tm^3+^ are good activator ions, because of their long-lived intermediate states for energy transfer.^307,308^ The host matrices offer a crystalline lattice structure for both the activator and sensitizer ions to conduct energy transfer,^307,308^ and should feature a low lattice phonon cutoff energy, so that the potential of nonradiative loss to the lattice is minimized and upconversion is favored. So far, NaYF_4_, NaYbF_4_, NaGdF_4_, NaLaF_4_, NaLuF_4_, LiYF_4_, LiLuF_4_, LaF_3_, YF_3_, GdF_3_, GdOF, La_2_O_3_, Lu_2_O_3_, Y_2_O_3_, and Y_2_O_2_S, have been used as host crystals.^309−313^ The PL resulting from upconversion originates from the 4f-4f orbital electronic transitions with concomitant wave functions from the lanthanide ions, featuring ladder-like arrangements of energy levels and allowing the occurrence of electron transitions between 4f levels.^314−316^ 4f-4f orbital electronic transitions are well shielded by the filled 5s and 5p shells, which results in line-like sharp emissions, with high resistance to photobleaching and photochemical degradation.^317^ The PL mechanisms in UCNPs can be classified into six main categories: excited-state absorption (ESA), energy transfer upconversion (ETU), energy migration-mediated upconversion (EMU), cooperative upconversion (CUC), cross-relaxation (CR), and photon avalanche (PA), which are illustrated in Figure 24b.^318,319^ ESA involves the successive absorption of two photons for upconversion emission.^320−322^ ETU is a more efficient process that includes the resonant energy transfer from the sensitizer ions to the activator ions. EMU makes use of four types of lanthanide dopants located in separate layers of a core–shell structure, chosen to facilitate the energy transfer between the accumulator and activator.^323^ In CUC, both the sensitization and the luminescence processes occur in cooperative fashion to improve energy transfer between adjacent ions.^324,325^ Cross-relaxation indicates an energy transfer process between activators with well-matching optical transitions.^326,327^ In the case of PA, the intermediate reservoir level of ions is initially populated by a nonresonant ground state absorption process, and then resonant ESA or ETU follow from another excited ion to populate the luminescent level and produce upconversion emission.^328^ The unique luminescence features of UCNPs endow them with particular performance characteristics for biological imaging, overcoming the common disadvantages of conventional probes, such as photobleaching, photoblinking, background autofluorescence, limited tissue penetration and phototoxicity.^318,329−331^

Taking advantages of these merits, UCNPs have been successfully used for STED imaging. In an early work, YAG:Pr^3+^ NPs were excited with visible laser light and exhibited UV emission, permitting background-free STED imaging with a resolution of ∼50 nm.^332^ However, the NP system used exhibited low efficiency, and the emitted UV light is toxic for biological samples, which limits application potential. An improvement is obtained through use of UCNPs doped with high concentrations of Tm^3+^ (8%). This facilitates the establishment of a population inversion via their intermediate metastable levels at optical excitation wavelengths, and thus enabled low-power (∼0.19 MW cm^–2^) STED imaging, with a resolution down to ∼28 nm (λ/36). Standard UCNPs that are doped at low levels (1%) have small cross sections for absorption and stimulated emission, and therefore require high intensities for high-resolution STED imaging (Figure 24c,d).^291^ Significantly lower excitation and depletion powers for STED were needed in Yb-based core–shell UCNPs (NaYb*x*Tm_1–*x*_F_4_).^333^ Another work revealed that under the assistance of interionic cross relaxation, 18 nm NaYF_4_:18% Yb^3+^, 10% Tm^3+^ NPs (Figure 24e) also lowered the laser intensity required for depletion and achieved two-color super-resolution imaging at 66 nm resolution.^292^ Moreover, pixel dwell times of only 100 μs enabled the high-speed STED imaging of cellular cytoskeletal protein structures (Figure 24f). Notably, in another work, a cyclometalated iridium(III) tubulin complex (cIr-Tub) was designed and used to perform correlative STED and EMs,^293^ permits STED imaging of tubulin localization and motion with a resolution of ∼30 nm (Figure 24g). UCNPs hold promise for STED nanoscopy in biology. A radically different approach makes use of MeV focused helium ions instead of lasers to excite NaYF_4_:Yb,Tm UCPNs.^334^ Here, Yb^3+^ and Tm^3+^ ions convert the energy of the helium ions to produce PL over hours long time periods. Compared with 980 nm laser excitation, the resolution was greatly enhanced from 253 to 28 nm. The method is extremely complex, however, and high-energy ion beams are harmful to biological samples. In another strategy, the use of downshifting NaGdF_4_:Nd (1%) NPs enabled STED imaging in all-NIR spectral bands under excitation at 808 nm, depletion at 1064 nm and detection over the 850–900 nm spectral band. Saturation intensities were low (19 kW cm^–2^).^335^ As a result, imaging in deep tissue was possible and at depths of 50 μm a spatial resolution of ca. 70 nm was achieved.

As a derivative method of STED, fluorescence emission difference (FED) imaging has been also been realized using NaYF_4_:Nd^3+^/Yb^3+^/Er^3+^@NaYF_4_:Nd^3+^ NPs. Using 808 nm cw laser excitation (10 MW/cm^2^) yielded 80 nm spatial resolution,^336^ without observation of photobleaching, a problem that plagues traditional STED.

In another study,^337^ blue and green emission were generated orthogonally in NaYF_4_:Er^3+^@NaYF_4_@NaYF_4_:Yb^3+^/Tm^3+^ nanoparticles, enabling single-scan FED microscopy using a 940 nm Gaussian beam and an 808 nm donut beam. Images were subtracted on the fly to increase imaging speed. Similarly, in efforts to lower laser powers for use in deep tissue super-resolution imaging, a 980 nm laser (5.5 MW/cm^2^) was used to generate the doughnut beam with detection at 800 nm. Remarkably, the authors were able to image individual NaYF_4_:Yb^3+^/Tm^3+^ particles through 93 μm thick liver tissue with a resolution of better than 50 nm.^338^

In ,photon-avalanche NPs (NaY_0.92_Tm_0.08_F_4_@NaY_0.8_Gd_0.2_F_4_) (Figure 24h) were directly excited on a conventional confocal microscope using 1064 nm excitation light. This NP system benefits from weak absorption in the ground state but extremely enhanced absorption in the excited state, with enhancements of order 10000. The extreme nonlinearity of the photon avalanching process, leads to a narrowing of the emission PSF that scales with the inverse square root of the nonlinearity. The authors demonstrated sub-70 nm spatial resolution using a conventional, single beam confocal microscope. Illumination was with a Gaussian profile at 1046 nm, a readily available wavelength with existing laser technology, and no further computational analysis was required to generate the images, making this a promising SRM imaging method in near-infrared spectral windows.

The giant nonlinear optical response from photoavalanching UCNPs (NaYF_4_:40%Yb^3+^/2%Tm^3+^) has also been exploited to obtain super-resolution via excitation of a single donut shaped excitation beam at 980 nm wavelength. The heterochromic response yields two emission states, one resulting from the 4-photon excited state, resulting in 740 nm emission, and another from the a 2-photon excited state, emitting at 800 nm. The emission states are thus chromatically distinguishable. However, because the 2-photon transition saturates much more quickly than the 4-photon transition, the two emission states yield very different PSF patterns. Subtraction of the patterns and deconvolution permitted a spatial resolution to be obtained of 40 nm at a relatively low laser powers (2.75 MW/cm^2^).^339^

UCNPs have also been demonstrated for SIM imaging.^340,341^ Jin et al. used UCNPs formed of NaYF4:Yb,Tm as probes,^340^ illuminated at 976 nm in the NIR spectral region. The material upconverts this radiation to emit at 800 nm. An interesting possibility in this context is offered by the highly nonlinear photoresponse of UCNPs. In SIM this can produce harmonics that convey high spatial frequency information on the sample through the microscope. This nonlinear variant of SIM can be realized under low-power excitation conditions with UCNPs (10^3^W/cm^2^) improving on the resolution of standard SIM. This was used to image UCNPs with a physical diameter of 40 nm. Two adjacent UCNPs could thus be resolved, with a resolution below 131 nm at an imaging rate of 1 Hz. Moreover, the result was achieved by imaging the UCNPs embedded deep within liver tissue, a promising result, that holds promise for new modes of tracking dynamic detail deep within tissue, with subcellular resolution.

However, UCNPs also have shortcomings. They suffer from poor water-solubility and low fluorescence quantum yields. Capping with hydrophilic ligands or postsynthetic modification is an option to improve their dispersibility in water. Through a careful core–shell design, their brightness can furthermore be enhanced, although improvements reported so far are modest.^342,343^ The nature of the electronic transitions involved in upconversion systems means that the excitation and emission bands are not tunable and cross sections for stimulated emission are small because the transition is parity-forbidden.^344,345^ These factors are not beneficial for STED imaging, and rational design strategies are needed to optimize host matrices, doping types, and concentration of lanthanide ions.^305,346−348^ For biological applications, the UCNP surface needs to be functionalized and again achieving small particle sizes is key for success in a physiologically relevant context. Further desirable characteristics include UCNPs that feature emissions in the NIR II spectral window (1000–1700 nm). This wavelength band offers optimal sample transmission for deep-tissue imaging. Given the fact that UCNPs are highly photostable without photobleaching, which hinders their use for STORM imaging. Finally, excitation powers must be kept low, because sample heating is a problem in the wavelength range relevant for UCNPs.^349^

### 3.8 Other NPs/Nanomaterials

In the following section, we cover carbon nanotubes and metal NPs as new classes of materials for which reported research in the context of super-resolution imaging is less established so far compared to the NP classes summarized so far. These materials do offer some promise, however, to progress the field.

Carbon nanotubes, CNTs, are a class of 1D carbon nanomaterials discovered in 1991.^350−353^ CNTs consist of single or multiple coaxial tubes of “rolled up” graphitic layers consisting of sp^2^ carbon atoms. The electronic and optical properties of CNTs are largely dependent on the direction along which the graphitic sheet is rolling with respect to lattice coordinates, the diameter of the nanotube, and the covalent intralayer bonding between the carbon layers. Specifically, the band gaps in semiconducting single-walled carbon nanotubes (SWCNTs) give rise to a range of PL properties.^354−357^ Absorption spectra lie in the visible (400–750 nm) and NIR-I (750–1000 nm) windows, generating PL emission in the NIR-II window (1000–1700) nm,^358^ which makes SWCNTs interesting candidates as probes for deep tissue fluorescence imaging.^359−364^

**Figure 25:**
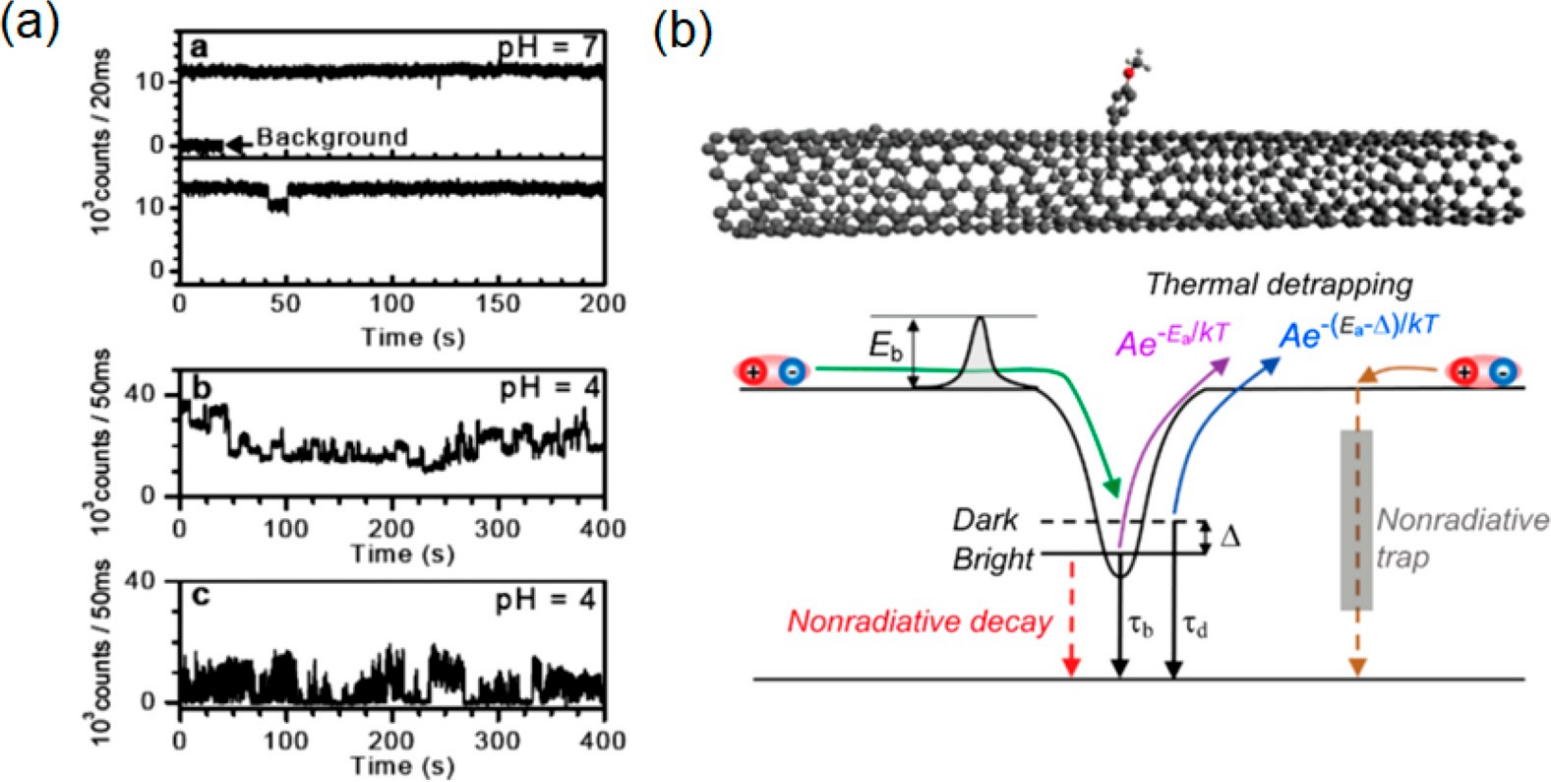
(a) Acid-induced PL intermittency of individual SWNTs at pH 4 and pH 7, respectively. Reproduced with permission from ref (365). Copyright 2008 American Chemical Society. (b) Schematic illustration of photophysical processes associated with the intensity fluctuation and decay dynamics of a defect-bound exciton. Excitons produced from the band edge diffuse and are trapped by the defects (green arrow). A potential barrier (of high Eb) or nonradiative traps could impede the diffusion or trapping of the excitons, as well as the random opening and closing of a nonradiative decay channel (red dotted arrow), leading to intensity fluctuation. Reprinted with permission from ref (366). Copyright 2019 American Chemical Society.

CNTs have been explored for use in SMLM imaging. The photoblinking behavior of SWNTs was first discovered under controlled acidic environments,^365^ A long-term intermittency in the fluorescence trace is visible (Figure 25a), which can be ascribed to individually localized protonation and deprotonation reactions. Similarly, by inducing certain reactions on the SWCNT surface, oscillatory fluorescence behavior from a single SWCNT has been observed.^367^ Specifically, the PL of SWCNTs can be quenched by riboflavin-generated reactive oxygen species (ROS), but it increases with Trolox, a ROS scavenger. Through regulation of the charge transfer process on the surface, this environmentally sensitive fluorescence response can be used as a dynamic probe for reaction conditions at the nanoscale. In another work, SWCNTs exhibited emission fluctuations at pH 6 in a phosphate-buffered saline solution.^368^ The surface intermittency spots of SWCNTs can be localized to reconstruct an image at 80 nm resolution. With the aid of the RNA-decorated SWCNT, the stochastic chemical kinetics of DNA walkers was revealed. For biological application, ultrasmall nanotubes exhibit an enhanced diffusion ability. Ultrashort SWCNTs (∼40 nm length) were found showing a photoblinking behavior,^369^ which is probably because of the transient defect charging induced by electrostatic interactions, a resolution below 25 nm was achieved on individual nanotubes. In a recent study, Htoon et al. ascribed the PL intensity fluctuations of SWCNTs to their fluctuation from defect exciton occupancy,^366^ which originates from random opening of nonradiative quenching sites intercepting the exciton population and/or the fluctuating potential barrier around the defect (Figure 25b). In order to figure out the blinking dynamics of the photoswitchable CNTs, Cognet et al. carried out simulation modeling and found that photoinduced blinking CNTs possessing arbitrary dynamics by adjusting the density of functionalization or illumination. So, the blinking rates of CNTs can be optimized for super-resolving densely labeled structures.^370^

**Figure 26:**
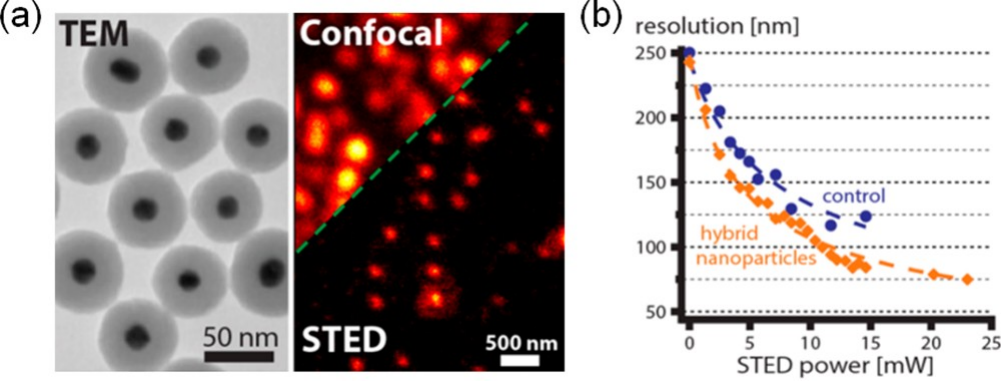
(a) Left: TEM image of the synthesized composite, that is, 20 nm gold nanospheres coated by 20 nm silica shell doped with Atto 488. Right: Confocal image and STED image of the composite. A 488 nm pulsed diode laser was used for excitation. A 595 nm Ti:sapphire laser (80 MHz) was used as STED beam. (b) Resolution comparisons of the synthesized composite between Atto 488 dye under different STED power. Panels a and b were reprinted with the permission from ref (371). Copyright 2017 American Chemical Society.

**Figure 27:**
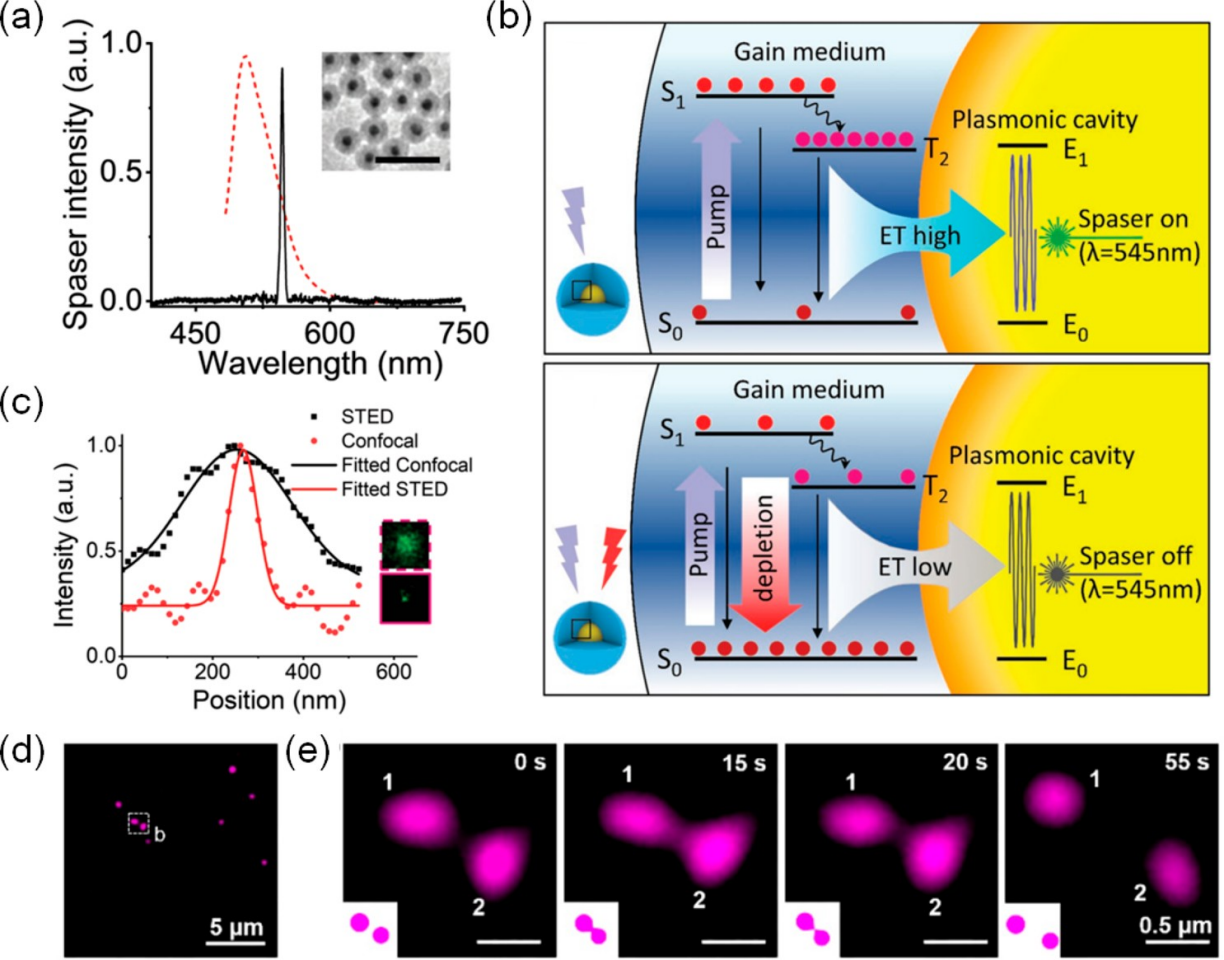
(a) Emission spectra of fluorescent dyes (dashed red line) and spaser NPs (solid black line). Inset: TEM image of spaser NPs. Scale bar: 100 nm. Excitation: 488 nm. (b) STED principle using spasers. The top panel shows the energy level and population distribution of the spaser NPs in the presence of excitation laser light alone, while in the bottom panel, a depletion laser is also applied. ET: energy transfer. (c) Typical intensity profiles extracted from spaser NPs in STED imaging and confocal imaging (inset). Panels a–c were reproduced with permission from ref (394). Copyright 2020 WILEY-VCH Verlag GmbH & Co. KGaA. SIM images of Cy5@Au NPs labeled lysosomes in HeLa cells before (d) and during (e) the kiss-and-run process. Panels d and e were reproduced with permission from ref (395). Copyright 2020 Ivyspring International Publisher.

Metal NPs (Au/Ag) or dye-doped metallic NPs have long been used to exploit strongly localized plasmon resonances (LPR), which offers opportunities to create reporter systems that are useful for bioimaging.^372−378^ The phenomenon results from the surface plasmon resonance (SPR) effect.^379,380^ When metal NPs are irradiated at specific wavelengths, electrons in the metal start to oscillate in resonance with the incoming field, which is caused by the interaction of conduction electrons near the metal surface with incident photons.^381,382^ The SPR endows metal NPs with unique optical properties including very large absorption and scattering cross sections. The use of small metal NP leads to strongly localized resonances that lead to hugely enhanced near-field amplitudes at the resonance wavelength.^383^ Different sizes, shapes, and local dielectric surroundings have a strong effect on the optical behavior of metallic NPs.^372,384−387^ Sivan and co-workers were the first to investigate how LPRs might be exploited in dye-coated gold NPs to improve STED imaging quality,^388^ in a technique termed nanoparticle-assisted STED nanoscopy (NP-STED). Specifically, there has been an interest to see whether the conjugation of reporter dyes to metal NPs can enhance their properties for STED imaging, such that the intensities required for efficient depletion can be lowered, and thus to improve sample compatibility for practical STED applications.^389−391^ Potentially, this would not only permit higher resolution imaging performance for STED, demonstrated for example with dye coated, nanorod shaped, gold NPs, but also the use of lower power, lower cost laser sources for STED imaging.^391,392^ It is thought that thicker metallic shells lead to better NP-STED performance, but this increases size, and therefore limits application potential for biological research. In more recent studies, 20 nm gold nanospheres were coated with a 20 nm shell formed of silica doped with Atto 488 dye for STED imaging (Figure 26a),^371^ yielding a 3.3-fold resolution improvement over diffraction limited confocal microscopy (Figure 26a). The method permitted a reduction in the depletion laser intensity by a factor of 2 compared to standard, dye-based, STED at a similar resolution performance (Figure 26b) with concomitant reductions photobleaching rates by a factor of 3. In addition, the LPR field enhancement does not only modify STED efficiencies but also makes changes to the spontaneous fluorescence behavior.^393^ So-called spasers (i.e., surface plasmon laser) nanoprobes have been explored as STED probes.^394^ They consist of a 20 nm gold core with a 13.5 nm shell of dye-doped silica (Figure 27a). As illustrated in Figure 27b, under pump laser illumination, electrons undergo a triplet-singlet transition between the T_2_ and S_0_ levels, which couples resonantly to the plasmonic cavity and facilitates spaser emission. When a depletion beam is subsequently switched on, electrons in the excited S_1_ state deplete rapidly to the S_0_ state, which suppresses a population inversion between T_2_ and S_0_ states and no spaser emission occurs. The work pioneers the use of spasers for STED imaging without spectral crosstalk by exploiting their very narrow emission line widths (3.8 nm) (Figure 27a). A resolution of 74 nm over a signal collection window of 10 nm bandwidth (Figure 27c).

Very recently, a super-resolution method was proposed called scattering saturation STED microscopy (ssSTED). The technique exploits the nonlinear response of backscattered light emitted from 50 nm plasmonic NPs made of silver when illuminated with intense laser light. Although not STED in the strictest sense, there are conceptual parallels. The technique makes use of the fact that the backscattered signal in the fully saturated regime has a donut shaped profile, whereas at low illumination intensity the backscattered light is Gaussian in profile. The authors split the laser into two beams, one at low intensity and temporally modulated, and the other at very high intensity, that is unmodulated. Superimposing the two beams to illuminate the sample and detecting the modulated part in the signal permits the picking out of a central region that is narrowed by the saturated donut, in conceptually analogy to STED, here enabled through the nonlinearity in the plasmonic response of the particles. The authors achieved a resolution of 65 nm (λ/7) with this approach to resolve the particles.^396^

Metallic NPs have also been applied for super-resolution techniques other than STED. For example, red emitting Au-NPs conjugated with bovine serum albumin have been used in SMLM super-resolution microscopy.^397^ On–off duty cycles were calculated to be ∼0.008 with an average “on” time of ∼200 ms. The reporters could be used to localize lysosome HeLa cells with a resolution of ∼59 nm. Pappas et al. proposed that the blinking rates of Rhodamine 110-doped Ag silica NPs^224^ can be regulated by changing the medium in which the particles are suspended, with a specific sensitivity to prevailing oxygen concentrations. In nitrogen rich/oxygen starved environments, the duty cycles of the NPs were found to be larger. In another study, cyanine 5 (Cy5) fluorophores were conjugated to the surface of Au NP cores,^395^ again enabling the labeling of lysosomes. The method could be used for SIM imaging in live HeLa cells and visualized kiss-and-run interactions between organelles (Figure 27d,e), as well as fusion, fission, and mitophagy processes. Despite these achievements, metal NPs or dye-doped metallic NPs are larger (above 40 nm) than carbon dots, quantum dots, and polymer dots (<10 nm) and their development for multiplexed labeling remains immature.

## 4 Functionalized Nanoparticles for Cellular Super-Resolution Imaging

**Figure 28:**
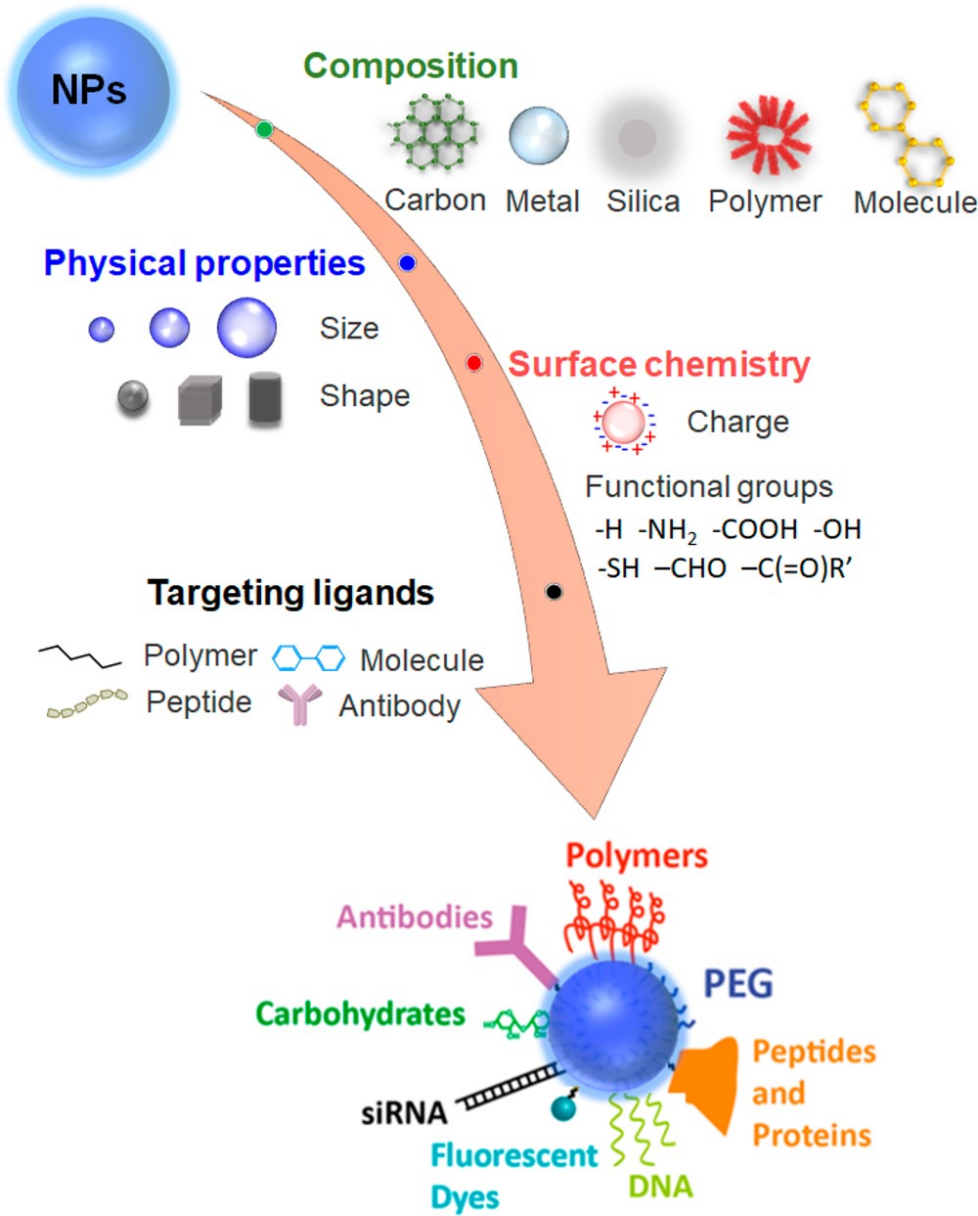
Strategy for NPs realizing intracellular delivery and targeting through regulating the composition, physical properties, surface chemistry, and targeting ligands. Adapted and reprinted with the permission from the ref (399). Copyright 2019 American Chemical Society.

The surface functionalization of NPs is of importance to develop these systems into effective probes for biological applications. Decorating NPs with ligands that target biological molecules with specificity and sensitivity is key to reach this goal. The large surface-to-volume ratio featured by NPs is a potential advantage in this respect, providing space for multiple attachment sites, but there are further requirements: Depending on the NP type, specific surface chemistries are required to improve water-solubility, stability, and to protect biological systems from toxic effects. Even though the photophysical properties of NPs have been carefully studied, their biological application is still at an early stage. Successful probes require efficient bonding to biomolecules of interest, including proteins, peptides, nucleic acids (i.e., DNA/RNA, oligomers, aptamers), lipids, antibodies, enzymes, etc. directly in the cell. Ideal systems have a high efficiency to traverse the cell membrane and are capable of reaching specific subcellular targets efficiently. General strategies for probe design and labeling for different NP classes are shown in Figure 28. We review these here with particular focus on systems suitable for super-resolution imaging.^43,398^

Properties that need to be considered to render NPs useful for bioimaging applications include their water-solubility, biocompatibility, and monodispersity. For nonmetallic materials such as CDs, PDs, NDs, AIE dots, and CNTs, the compatibility with biological systems is usually good and concentrations up to tens of μg/mL are often tolerated with negligible toxic phenotypes.^383^ However, apart from CDs, their water solubility is poor. NDs, for example, aggregate readily in cellular environments.^278^ Metal-based NPs are usually hydrophobic and also tend to aggregate in aqueous solution. On their own without protective coating, they are often highly toxic to cells.

To address such issues, three approaches are commonly used for surface modification of NPs.^400^ The first strategy improves water solubility through synthesis from water-soluble precursors and use of stabilizing ligands.^401^ This has been successful for the synthesis of water-soluble QDs, PDs, and modified silica UCNPs. The second method uses postsynthesis ligand exchange, so that water-soluble groups such as −OH, −COOH, thiols,^402−409^ polymers,^410−416^ or silica^417−419^ replace the hydrophobic ligands on the originally hydrophobic surface. The third approach involves the encapsulation of hydrophobic NPs in polymers or in other hydrophilic matrices. The NPs themselves are attached to the matrix by hydrophobic or electrostatic interactions. Amphiphilic polymers are excellent coating materials in this context, because their polymer chains feature hydrophobic subunits for binding with hydrophobic NP cores,^420−423^ while exposing their hydrophilic part to the solvent, thus conferring hydrophilic properties overall to the encapsulated NPs. Silica coatings,^424−426^ liposomes,^427−429^ and other surfactants^430^ can also be used for encapsulation.

For conjugation with biological molecules, both covalent and noncovalent bonds are used. In the former case, the binding occurs directly between the molecule of interest and reactive ligands on the NP surface. Covalent bonds can be realized via catalysts and functional cross-linkers. Homo-, hetero-, and trifunctional cross-linkers are available, varying in size and type of cross-bridging available. For water-soluble NPs, carboxylic acid groups are often used, which can be enriched by chemical oxidation and acid treatment using thiogycolic acid (TGA) and dihydrolipoic acid (DHLA).^105,278,434,435^ Carboxylic acid groups can be easily reacted with amino groups of proteins, peptides, DNA, and immunoglobulins via *N*-hydroxysuccinimide (NHS) and 1-(3-(dimethylamino)propyl)-3-ethylcarbodiimide (EDC)-catalyzed amidation.^436^ For example, through EDC catalysis, UCNPs are able to bind to DNA. On conjugation with graphene oxide, this composite can subsequently be used for the detection of zeptomoles of target oligonucleotides in solution.^437,438^ Moreover, amine terminated NPs can be biotinylated by the reaction with biotin NHS or biotin-sulfo NHS ester. Carboxyl and hydroxyl groups are normally found on hydrophilic NPs.

**Figure 29:**
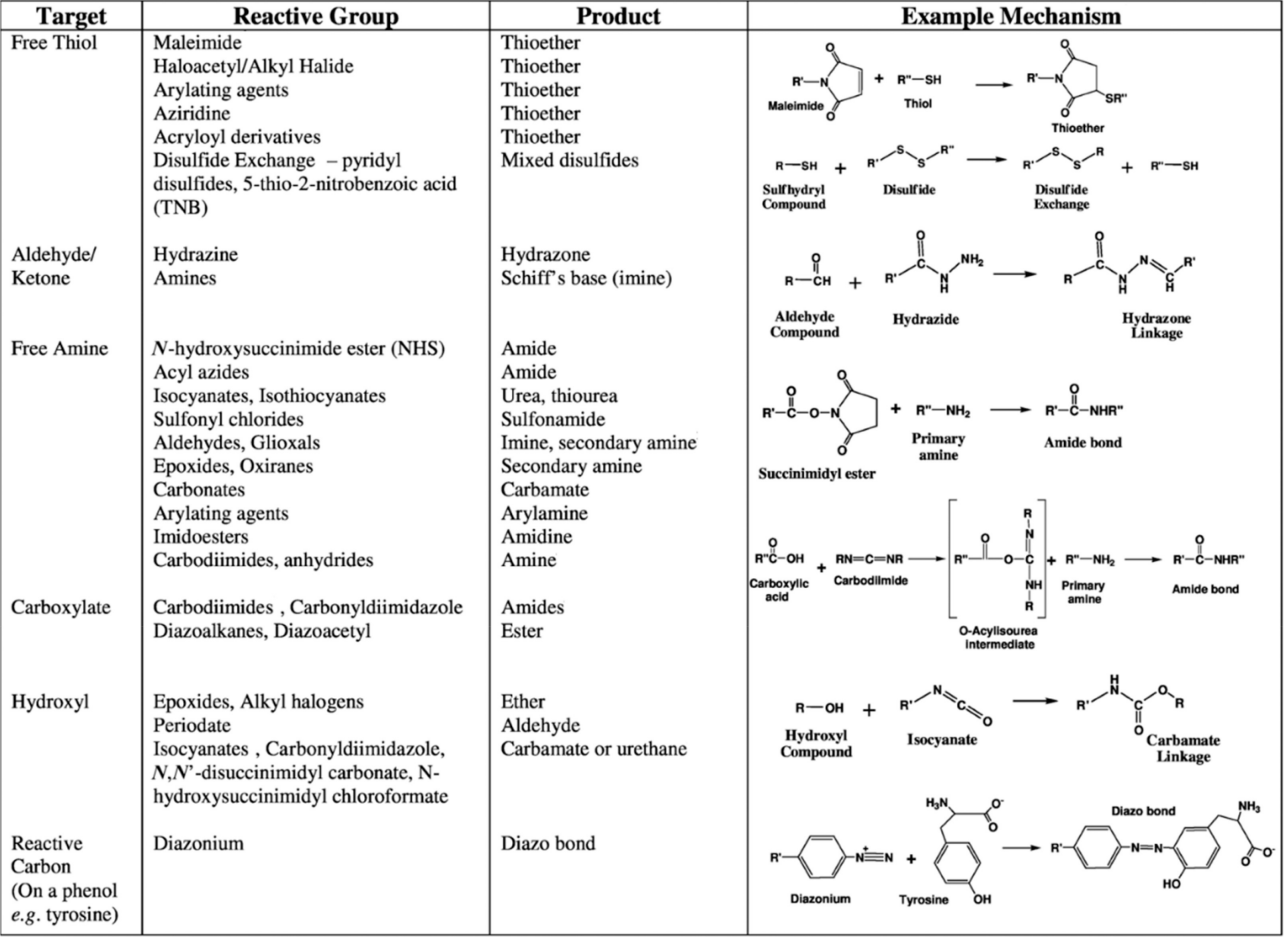
Common strategies and reaction mechanisms for the surface functionalization of NPs. Reprinted with permission from refs (431−433). Copyright 2008 Academic Press. Copyright 2009 Wiley-Blackwell. Copyright 2013 American Chemical Society.

Hydrophobic metal NPs have a high affinity to thiols, reaction with which confers excellent colloidal stability on this NP class.^439^ Other coupling chemistries are also available, such as maleimide conjugation to free thiols, aniline-catalyzed hydrazine binding with amino groups, and diazonium modification of the phenolic side chain of tyrosine.^431,432^ Specifically, the conjugation of carboxyl, amino, and sulfhydryl functionalized NPs to desired biomolecules can be realized based on commonly used bifunctional cross-linking reagents such as EDC, diisopropyl carbodiimide (DIC), *N*-succinimidyl 3-[2-pyridyldithio]-propionate (SPDP), ulfosuccinimidyl-(4-N-maleimidomethyl)cyclohexane-1-carboxylate (sulfo-SMCC) and maleimido succinimide. Figure 29 displays common conjugation strategies for different chemical groups and corresponding reaction mechanisms. The specific conjugation approach to be adopted for labeling a molecule of interest depends on NP surface groups and linker availability. For modified silica NPs, for example, surficial Si–OH does not react efficiently with common linkage groups (carboxyl, amine, etc.). Here, silane linkers, e.g., APTES, can be used for subsequent conjugation with functional molecules via amidation.^440−442^

Noncovalent binding is mediated by hydrophobic, electrostatic, or high affinity interactions between the NP surface and the biomolecule of interest. The self-assembled NP bioconjugates can often be produced simply by stoichiometric mixing of the two components, which is rapid and facile but depends sensitively the concentrations of NPs and biomolecules to be labeled, and the environment in which they reside.^443^ Electrostatic interactions between molecules of opposite charge are widely used.^444^ For example, negatively charged QDs with −COOH surface ligands can bind to positively charged proteins. Nucleic acids, on the other hand, can be conjugated to positively charged NPs via their negatively charged phosphate backbone and similar approaches have been taken for other labeling strategies. Despite the simplicity of electrostatic binding, which requires no complex reagents or cross-linkers, there are limitations. Binding via electrostatic interactions is comparatively weak and sensitively dependent on ionic strength, pH, and the type and magnitude of charges involved in the interaction.^445−448^

A common noncovalent bioconjugation strategy employs the avidin/streptavidin–biotin interaction.^431,449−453^ Here, NPs and biomolecules are ligated with biotin and avidin/streptavidin, respectively, or vice versa, and a noncovalent, but very strong and specific, avidin/streptavidin–biotin complex forms to yield the conjugate. The method is popular for tethering DNA, proteins, or antibodies onto the surface of NPs and it is much less dependent on environmental factors (pH, etc.) than electrostatic conjugation. The method provides a tool, furthermore, for secondary ligation to the NP surface. For example, if recognition of a biomolecule is not efficient with an NP conjugated with a primary antibody, it is possible instead to achieve recognition in multisteps, e.g., using a biotinylated primary antibody that binds to the biomolecule, and this conjugate is finally bound to the NP via avidin/streptavidin–biotin linkage.^454^ Secondary interactions such as receptor–ligand and antibody–ligand interactions have also been frequently investigated using similar approaches.^455,456^

The coating of the surfaces of NPs or their encapsulation are important to their functionality. NPs are usually coated with organic (monomeric and polymeric), inorganic (metallic and oxidized), or biomolecular layers. For example, for inorganic NPs, such as QDs,^457,458^ modified silica NPs,^459^ and metallic NPs,^460^ poly ethylene glycol (PEG) is commonly used. PEG coatings confer good biocompatibility and hydrophilicity and stabilize NP suspensions, preventing aggregation and reducing nonspecific binding.^461,462^ PEG coated NPs can be further conjugated to molecules of interest, such as peptides, antibodies or fluorophores. PEGs can act as cross-linkers/spacers and enable facile conjugation strategies,^463^ while at same time protecting the hybrid particles from their environment.^464−467^

Silica coating of NPs prevents oxidation or decomposition of NPs and can reduce their toxicity dramatically.^468−470^ Through the sol–gel method involving the hydrolysis and condensation of alkoxysilane, silanol-terminated surfaces are formed on the NP surface. Functional groups can be introduced during either the condensation process or by postsynthesis surface modification.^471^ Commonly, 3-aminopropyl tri(m)ethoxysilane and 3-mercaptopropyl tri(m)ethoxysilane are used in the condensation reactions, providing primary amines and thiol groups on the NP surface.^472^

An important encapsulation strategy involves the protein bovine serum albumin (BSA). BSA is a protein present in blood, and BSA modified NPs have improved circulation half-lives and are able to target biomolecules of interest.^473^ As an example, BSA coated onto alkyl-thiol terminated NPs via hydrophobic interactions improved water dispersibility and prolonged fluorescence properties.^474^

The strategies presented here offer great flexibility to manufacture conjugates with various biological functionalities. Linkage of specific peptides and proteins can be used to optimize cell uptake and tissue penetration, to reach cellular target sites, and to improve the specificity and sensitivity with which cellular pathways can be probed and manipulated.^475^ For example, NPs conjugated with cell-penetrating peptides have been shown to exhibit improved uptake and delivery properties,^476^ while so-called homing peptides permit the specific targeting of cells and tissues; particularly interesting for the targeting of tumor cells. The conjugation of peptides depends on NP surface properties. For example, for Au NPs, peptides featuring cysteine can bind directly via the free thiol (−SH) group in the side chain of cysteine.^477−480^

**Figure 30:**
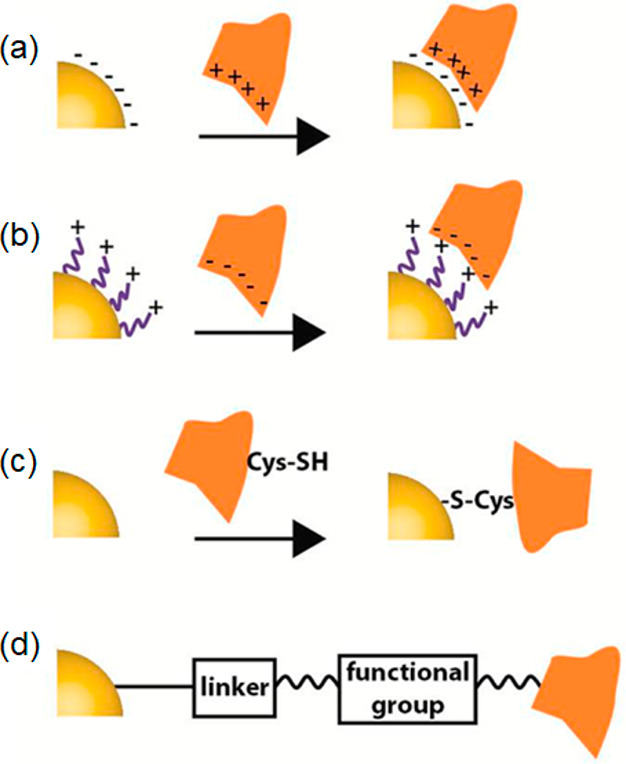
Methods for the conjugation of NPs with biomolecules. (a) Electrostatic interactions can be used to couple positively charged molecules, e.g., proteins, directly to negatively charged NPs. This method is facile to implement but very dependent on environmental parameters such as ion concentration, pH, etc. (b) Conjugation of biomolecules through introduction of charged ligands on the NP surface permits the labeling with weakly charged NPs. (c) Covalent linking via functional groups (e.g., Cys-SH or Lys-NH_2_), here shown for the reaction of thiols in cysteines with a metal NP surface. This is a strategy commonly used for gold NPs. (d) Covalent binding via a bifunctional linker. Panels a–d were reproduced with permission from ref (399). Copyright 2019 American Chemical Society.

In addition to direct coupling, peptides can also conjugate with ligands present on the NP surface. For example, EDC/sulfo-NHS coupling has been employed to modify OEG-capped NPs with peptides. Such systems were demonstrated in studies of endothelial cells in the context of angiogenesis both *in vitro* and *in vivo*.^479,481−487^ As for protein binding, the most common way is to use the avidin/streptavidin–biotin system described.^488−490^ General principles of electrostatic and covalent binding approaches are shown in Figure 30.^399^

**Figure 31:**
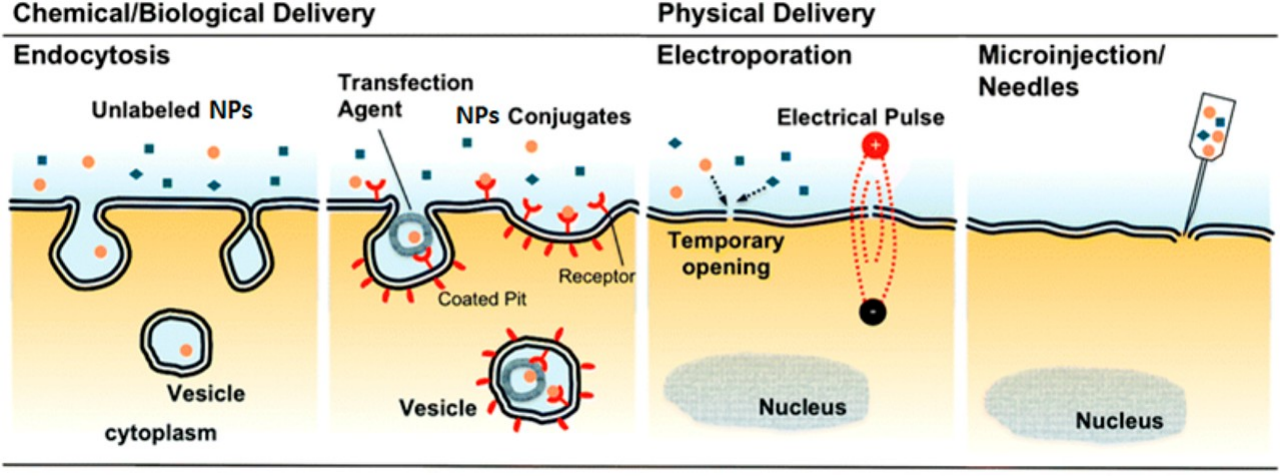
Approaches for NPs cellular internalization, including the chemical/biological delivery and physical delivery. Adapted with permission from ref (491). Copyright 2010 Royal Society of Chemistry.

**Figure 32:**
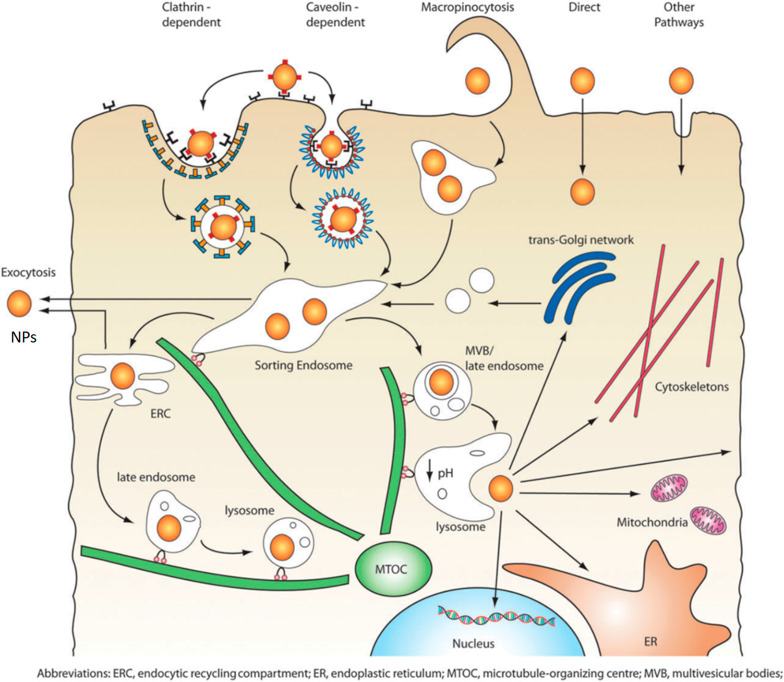
Intracellular transport pathways of NPs. In the endocytic pathways, NPs are transported along the endolysosomal network within vesicles with the help of motor proteins and cytoskeletal structures. NPs inside the vesicles can either undergo recycling and alternatively cellular expulsion or trafficking to the organelles including the lysosomes, Golgi, and mitochondria. Reprinted with permission from ref (510). Copyright 2011 Royal Society of Chemistry.

Bioconjugated NPs can be used as labels and tags for the analysis of cellular events, for example to visualize membrane bound receptor proteins, transport of intracellular cargo, delivery and uptake of molecules, the monitoring of organelle and cellular dynamics, and the labeling of tumor cells.^492−496^ Understanding the route of NP uptake and fate is vital for these tasks. NPs must be able to traverse the cellular membrane which can be achieved by chemical, biological, and physical delivery mechanisms as illustrated in Figure 31. For chemical/biological delivery, endocytosis is the main transport pathway.^497−500^ Internalization into cells can be direct or mediated by membrane-embedded receptors. On contact with the cell membrane, hydrophobic and electrostatic interactions cause the plasma membrane to be invaginated, causing the NP to be engulfed in a process called pinocytosis. The NPs can then be internalized (endocytosed) inside vesicles. Receptor-mediated endocytosis occurs NPs conjugated with ligands that bind to membrane bound receptors. This confers specificity and leads to the recruitment of receptors to clathrin through adaptor proteins, and efficient transport into the cell via clathrin-mediated endocytosis. Internalized NPs can undergo endocytic recycling (Figure 32) to be returned to the plasma membrane for cellular expulsion or trafficked to organelles including the lysosomes, Golgi, and mitochondria. Motor proteins shuttle vesicles along microtubules within the cell, so that they can be processed, sorted, fused or dissociated, to form endosomes and lysosomes.^501−504^ Entrapment of NPs inside vesicles can be undesirable and mask the function for which they were intended. To overcome this problem, pH-sensitive synthetic peptides or membranous envelopes have been used to coat NPs,^505^ the former to disrupt vesicle membranes and release the NP and the latter fuse with endosomal membranes. NPs have also been coated with polymers that can act as proton scavengers, allowing NPs to escape the endosomes through the so-called “proton-sponge effect”. The result is osmotic swelling and rupture of the endosome due to the proton-absorbing polymer, and thus escape of the NP.^506−508^ In another example, QDs were conjugated with cell penetrating peptides to be entrapped by vesicles and subsequent transport to the microtubule-organizing center, situated in the perinuclear region of the cell.^509^

The impact of NPs on cellular homeostasis was investigated in several studies.^511−514^ Without postfunctionalization, the physical properties of the NPs themselves affect their fate. All of their composition, size, surface groups, charge, etc. influence uptake, transportation, and accumulation inside the cell.^513,515−517^ CDs, for example, are inherently soluble in water and are highly compatible with biological systems. They can endocytose into a cell without further modification, and this property was exploited in their use as probes for SOFI imaging in cells.^107^ Blue emitting CDs thus delivered to the cells were seen to penetrate the nuclear membrane and localized in the nucleus, while green CDs accumulated preferentially in endosomes and lysosomes. This differential behavior was attributed to differences in surface charge or chemical groups. Other super-resolution imaging studies of various CDs found them to accumulate in the cytoplasm,^113,518^ and to associate to mitochondria.^115^ In the case of QDs, positively charged QDs were found to accumulate in the nucleus, while negatively charged QDs remained in the cytoplasm.^519^ When QDs were capped with ligands such as thiols (DHLA, TGA, d-penicillamine), nonspecific binding to the membranes of HeLa, neuroblastoma, monocytic, and NIH 3T3 fibroblast cells were reported.^520−522^ Although coatings can be designed to offer differential affinity for adhesion to particular membrane types, they are often nonspecific.^189,523^ For example, QDs conjugated with polymers,^507,524^ liposomes,^428,525^ and lipids^526^ were seen to undergo nonspecific endocytosis in different cell types. To facilitate the efficient internalization of NPs into cells, QDs modified with polymer ligands (e.g., polyethylene glycol (PEG)) and then conjugated with specific fusion protein can be transported into the nuclei of HeLa cells. For CDs, zwitterionic surface functionalization imparts good colloidal stability and was seen to cause CDs to translocate from cytoplasm to the nucleus.^527^ Decoration of CDs with cell-penetrating peptides has also been used as coatings for the intracellular delivery of CDs.^528^ Peptides such as TAT^492^ and calcitonin^529^ deliver CDs to the cytosol, and incorporation of nuclear localization signals permit the subsequent guidance to the nucleus.^530,531^

Unlike small molecular dyes with selectivity for cellular substructures, for instance, DAPI, Mitotracker, and Lysotracker, which can penetrate cell membranes by passive diffusion, the much larger size of NPs requires active transport pathways, such as receptor-mediated and ligand–receptor-mediated endocytosis for cellular uptake. QDs have been functionalized with the epidermal growth factor (EGF), which targets the EGF receptor (EGFR) on the cell membrane.^135^ Wang et al. formed NPs from a mixture of transferrin protein and Atto647N dye.^229^ On mixing these components and cross-linking with glutaraldehyde, NPs were obtained that are readily transported into cells. Because transferrin itself targets transferrin-receptor proteins on the cell surface, the dye NPs were readily taken up by endocytosis and could be imaged by STED super-resolution imaging.

Physical methods have also been employed to provide transport pathways for NPs. Electroporation can be used to produce temporary hydrophilic pores in a cell membrane, for example used to transfer BSA-conjugated NDs into cells.^278^ With STED, individual cytosolic NDs were identifiable in cells distinguishable from particle aggregates trapped in endosomes. Photoporation is conceptually similar, but both techniques are invasive and lead to significant cell death, and they are usually less efficient than endocytosis-mediated uptake. However, an advantage is that using localized fields, specific locations in tissue or cell cultures can be targeted with physical techniques, whereas endocytosis-mediated processes affect the whole cell population.

Specific targeting of structures and subcellular compartment of interest is feasible through the use of functionalized NPs.^531,532^ In another investigation, through the electrostatic attraction, negative CDs were used to label the KI4 peptide self-assemblies, which contain numerous positive amino groups on their surface, enabling the high-density loading of CDs onto the assemblies. STORM permits the distribution of CD-stained peptide self-assemblies to be visualized and ultrastructural features were resolved.^111^ For visualizing subcellular compartments, the biotin–streptavidin recognition gains large popularity among super-resolution techniques.^533^ Thus, biotinylated antibodies and streptavidin-conjugated NPs are frequently used for efficient targeting. The most common example of this approach is the immunostaining of microtubules in cells with primary antibodies and streptavidin-conjugated NPs.^27,136,150,210^ In one example, the performance of STED, SIM, and SOFI imaging of a microtubule network was compared by staining the cells with streptavidin conjugates of QDs. In addition, the authors were able to image the distributions of membrane proteins in the cell. For instance, G protein-coupled receptors (GPCRs) clusters are protein aggregates with sizes in the nanometer range, which cannot be resolved by conventional microscopy. The authors labeled the chemokine receptor CCR3 with primary antibodies and CD-stained secondary antibodies. The latter permitted super-resolution imaging of receptor assemblies.^111^

## 5 General Strategies to Enhance the Performance of Nanoparticles for Biological Super-Resolution Imaging

The specific discussion around the different NP classes discussed so far permits us to draw some general conclusions on the applicability and promise of NPs for super-resolution imaging.

CDs and QDs offer excellent photostability and brightness, which favors their use for STED and SIM imaging applications. However, the high duty cycles of their photoemission do not make them as promising for use in SMLM applications. Progress here requires surface engineering or the construction of hybrid systems to lower the duty cycles. For example, nitrogen doping of CDs can lower duty cycles to below 0.3%. Similarly, the blinking of QDs can be controlled in hybrid core–shell systems, where excitation is followed energy transfer to an electron acceptor. Much more work is needed to render these systems into practical probes for SMLM imaging applications.

PDs and modified silica NPs, however, are more flexible in this regard and they improve both the photostability and photoblinking characteristics of their guest fluorophores (e.g., organic dyes, polymers). These advantages are offset, however, by the vastly increased size of these NP systems compared to the fluorophores on their own, and this poses limitations for biological use.

On the one hand, AIE dots have shown excellent potential for STED imaging, because of their large Stokes shifts and saturation behavior. Some AIEs are photoactivatable; there is thus some promise for future developments in SMLM imaging. NDs, on the other hand, are extremely photostable with excellent PLQYs. They offer the best resolution for STED imaging and SMLM is in principle possible, through the introduction of surface defects. As for AIEs, the use of NDs is in SMLM is in its infancy. A disadvantage in biology is again the problem of functionalizing ND probes for targeted delivery. UCNPs are other highly photostable systems, but photoblinking has not been reported yet for these systems.

How can the performance of NPs be improved for super-resolution imaging? In what follows, we consider some general potential strategies for the various NP classes.

For CDs and QDs, surface states play a dominant role in their photoblinking and photoswitching behavior. For CDs, the current consensus is that the emission intermittency comes from surface groups that produce energy wells for accepting the ejected electrons. The ensuing electron transfer process leads to fluorescent on- and off-states. The blinking characteristic of CDs can thus be adjusted through rational surface design, e.g., addition of electron accepting and donating agents. In the case of QDs, nonradiative recombination via surface traps or charging-induced Auger recombination severely affects the blinking behavior. The decrease of on-to-off duty cycles could be achieved via core passivation using thicker shell materials, the grafting of ligands onto the QD surface, contacting QDs with other NPs, electrostatic gating, the construction of hybrid blinking systems, and irradiation with ultrafast mid-infrared (MIR) pulses. For STED imaging, CDs and QDs could be improved, if multiphoton emission is effectively suppressed under illumination with high-power depletion lasers. On the one hand, suppressing background signal from the STED laser in the first place is, of course, preferential to reported methods requiring background subtraction in a postprocessing step. On the other hand, future CDs or QDs should be designed to feature narrow excitation spectra, emission at long wavelengths, and improved PLQYs. For CDs, promising avenues here are doping with elements (N, S, F, and so on) and incorporation of aromatic structures; for QDs, approaches include change of surface ligands and core size.

Developments for PDs for STED imaging include improvement of their photostability and the opportunity for multicolor imaging. The latter requires engineering of the particle surface and doping the PDs with different semiconducting polymers or fluorescent dyes. Spectral characteristics are governed by particle size, which is challenging to control in PD synthesis. The nanoprecipitation method enables small sizes to be obtained, and PDs thus produced showed improved photoblinking and emission intermittency, potentially taking their use beyond STED applications. Hole palarons may be exploited to further manipulate their blinking behavior. Doping of silica NPs may be similarly used to improve brightness and photoblinking.

AIE dots are usually hydrophobic. Their modification with functional groups or coating with hydrophilic matrices will enable water-soluble AIE dots to be obtained for bioimaging applications. Their optical performances are largely dependent on the AIE luminogens (AIEgens). Linking different AIE moieties can lead to red-shifted emission.^534^ In addition, the donor–acceptor structure constructed by electron donating (e.g., methoxy) or withdrawing (e.g., benzothiadiazole, benzobisthiadiazole) units in AIE dots can result photoluminescence systems that are tunable from the visible to the NIR spectral regions.^535^

Nanodiamonds feature very high photostability and brightness, even in the deep red or NIR regions. Their synthesis requires high-temperature and high-pressure methods. Heating/annealing cycles during periods of irradiation can result in a higher density of NV centers, which improves the brightness. The photoluminescence spectrum in NDs largely depends on the annealing temperature of the irradiated particles. Despite their superlative optical properties, a huge drawback is their size, and nanodiamonds with sizes less than 30 nm have not yet been achieved. The manufacturing process is complex and obtaining materials with uniformly distributed NV centers is difficult to achieve in practice.^536^ For bioimaging, surface functionalization is essential. Both noncovalent and covalent conjugation is possible with NDs, but precise cellular targeting remains a challenge with these large systems.

In the synthesis of UCNPs, hydro(solvo)thermal and thermal decomposition methods enable the preparation particles with controllable crystalline phase, size, and morphology. The coprecipitation approach is superior in obtaining high-purity and precise stoichiometric UCNPs and surfactants can be introduced to improve solubility functionalize the surface. Dopants can again be used to vary photoluminescence characteristics. Other approaches, such as host lattice manipulation, surface passivation, surface plasmon coupling, photonic crystal engineering, combination with other moieties for the facilitation of energy transfer, and construction of inorganic–organic hybrid systems, are all interesting strategies to enhance on current systems.^55^ For biological imaging, using Nd^3+^-sensitized or dye-sensitized UCNPs irradiated at 800 nm can address the issue of overheating under 980 nm illumination.^537,538^ Especially for STED applications, this is important. The overheating effect caused by the high-density depletion laser might be alleviated by an energy transfer or photoavalanche mechanism requiring lower saturation intensities. Exciting strategies also include the integration of UCNPs into photonic bandgap structures, e.g., via self-assembly of UCNP containing building blocks into photonic crystal structures. It was demonstrated with such an approach that photoluminescence lifetimes can be dramatically shortened. This would improve the imaging speed in STED applications.^539,540^

## 6 Conclusions and Persperctives

The emergence of optical super-resolution methods has revolutionized the field of biological imaging. Key biological discoveries were enabled both by a better understanding of the physical principles underlying these methods, and, crucially also, through the availability of better reporter systems whose properties are matched to specific imaging techniques.

High-resolution fluorescence imaging is always a compromise among image resolution, speed of acquisition, and compatibility with the biological system under investigation. Observing fast cellular processes such as organelle dynamics and transport at super-resolution remains to be a huge challenge in the field. The ideal probe needs to emit a maximal number of photons for minimal excitation fluxes during the observation window. From a biological perspective, the reporter system must be both specific to detect the entity one desires to study, and nonintrusive to not perturb the biological system under study.

Fluorescent reporters in use for super-resolution imaging are traditionally based either on organic dyes, or fluorescent proteins. Both systems have their shortcomings. While the former is superior in terms of brightness, the biological flexibility is limited, and a gamut of efficient probes is not available for all super-resolution variants. Fluorescent proteins on the other hand are outstanding from a biological perspective but their photophysical properties are far from optimal.

The ideal probe should possess a small size, exceptional brightness, and feature a low toxicity and high photostability. The probes should offer a good range of excitation and emission bands that enable multiple species to be differentiated simultaneously. Furthermore, photoswitching and blinking properties should be controllable to match the super-resolution method for which the reporter is intended.

In this review, we have surveyed the current state of the art of fluorescent nanoparticle systems and their optimization for biological super-resolution imaging. Confinement effects and the high surface-to-volume ratio of NPs permit design options that are not available with traditional reporter systems. The field is at the interface of materials science, chemistry, physics, and the biological sciences, and huge progress has been made in optimizing photophysical and functional properties of NP systems to realize specific imaging tasks at super-resolution.

We have focused this review primarily on the application in the biological sciences, but there is ample opportunity also for application in nonbiological systems. For example, the location and number of nitrogen vacancies can be measured with nanometer resolution in NP systems.^279^ The distribution of functional ligands on the NP surface can, on the other hand, be mapped and quantified by PAINT or DNA-PAINT.^541,542^ There are also opportunities to study materials and processes that are too delicate to be subjected to nonoptical microscopy techniques,^543^ or where no contrast can be achieved between different functional domains. Examples include block copolymer assembly processes^544^ or phase transition behavior and hydrogel formation.^545,546^

In biological systems, the superior brightness of NP systems and their photostability permits the tracking of subcellular entities, or labeled molecules over extended periods of time with high resolution. However, the field is still in its infancy, and much of the effort so far has been placed on the development of new NP materials and their photophysical characterization. A majority of work is still proof of principle in nature, at the technological appraisal stage. Progress in the field now requires biological application and not just proof of concept study. For this to happen, material chemists need to work side by side with biologists. Bright and highly specific probes would greatly enhance the arsenal of bioimaging tools available and enable the biologist to study subcellular phenomena in the context of health and disease. Indeed, the further advances in the field of super-resolution imaging are more likely to stem from advances in probe technologies than from optical physics.

There is excellent potential. The large surface area of NPs permits efficient coupling chemistries to be carried out, and the brightness and flexibility of material choice permit functional reporters to be designed that are optimal for a given application. Anchoring NPs to the membranes of cellular organelles such as the ER, for example, would permit the imaging of organelle peristalsis over hours and perhaps longer,^547^ at better resolution than has so far been possible, perhaps shedding light on such intriguing, recently discovered phenomena. If NP probes were designed with a capability to cross into the intraluminal space of the ER, a better understanding of molecular transport might ensue. This in turn might shed light on how the ER manages to distribute the products it synthesizes throughout the cell volume so efficiently, despite the fact that there is no known active transport machinery within its luminal space.^9^ Designing probes specifically for requisite biological experiments offers outstanding opportunities for high impact research.

Other NP enabled SRM modalities are on the horizon. For example, a variant of PAINT can be realized through electrostatic coupling or hydrophobic interactions between fluorophore and the sample. This is in contrast to DNA-PAINT, which utilizes transient oligonucleotide hybridization to enable the method.^548^ Using NPs as labels is rarely reported in these two techniques. Existing methods make use of fluorescent proteins, antibodies, and organic dyes and there are limitations. Multicolor imaging is difficult with these systems.^549^ Another problem for DNA-PAINT is the low acquisition speed, which is limited by the binding of the imager strand to its target. Strategies, such as the use of FRET, have been used to increase imaging speed without increasing background noise;^550,551^ however, this comes at the cost photobleaching for dyes such as Atto447N.^551^ NPs are superior in terms of photostability and chemical flexibility and multicolour imaging applications are thinkable, because of the narrow excitation and emission bands some systems offer. Technical challenges again relate to size and functionalization of NPs to be compatible with biological end use.

There is potential also to use NPs as catalysts for biochemical reactions. For example, the surface of NPs could be used to control rates of protein folding, aggregation, etc. either in efforts to gain a better understanding of these phenomena, or to find modes for therapeutic intervention. Theragnostic approaches are conceivable with functionalized NPs targeting specific subcellular domains to deliver functional molecules conjugated to their surface. Luminescence from the NP thus permits cargo tracking, at super-resolution, and its delivery elicits a functional response in the cell. The approach could be used to target cancerous tissue, for example. Photophysical interventions are also conceivable, e.g., via particle heating and interaction with local tissue.

Finally, fluorescent NPs may enable completely new modalities for correlative imaging. They could be designed as dual contrast probes for correlative EM and light microscopy or used as robust optical probes for samples subjected to AFM, electrophysiological, or mass spectrometric measurements. Computation will play a major role in such efforts, to deal with extremely rich and large data sets generated by such methods. There are huge challenges ahead, and therefore opportunities, for interdisciplinary science in the field.

## ABBREVIATIONS

NP: nanoparticle
SRM: super-resolution microscopy
EM: electron microscopy
SMLM: single-molecule localization microscopy
STED: stimulated emission depletion microscopy
GSD: ground state depletion microscopy
FPALM/PALM: fluorescence photoactivated localization microscopy/photoactivated localization microscopy
*d*STORM/STORM: direct stochastic optical reconstruction microscopy/stochastic optical reconstruction microscopy
SIM: structured illumination microscopy
SSIM: saturated structured-illumination microscopy
SOFI: super-resolution optical fluctuation microscopy
MINFLUX: minimal emission fluxes
CLSM: confocal laser scanning microscopy
SIM: structured illumination microscopy
FED: fluorescence emission difference
PAINT: points accumulation for imaging in nanoscale topography
PSF: point spread function
PLQY: photoluminescence quantum yield
CD: carbon dot
QD: quantum dot
PD: polymer dot
AIE: aggregation-induced emission
ND: nanodiamond
UCNP: upconversion nanoparticle
ER: endoplasmic reticulum
PL: photoluminescence
SRRF: super-resolution radial fluctuation microscopy
fwhm: full width at half-maximum
DLV: diffraction limited volume
EGFP: epidermal growth factor receptors
ICA: independent component analysis
CV: crystal violet
JT-SOFI: joint-tagging SOFI
MIR: mid-infrared
FONP: fluorescent organic nanoparticle
FRET: Förster resonance energy transfer
PS: polystyrene
STEDD: stimulated emission double depletion microscopy
AIEgens: AIE luminogens
ACQ: aggregation-caused quenching
TPE: tetraphenylethylene
TTF: 2,3-bis(4-(phenyl(4-(1,2,2-triphenylvinyl)phenyl)amino)phenyl) fumaronitrile
TPF: two-photon fluorescence
NV: nitrogen-vacancy
ESA: excited state absorption
ETU: energy transfer upconversion
PA: photon avalanche
SWCNT: single-walled carbon nanotube
ROS: reactive oxygen species
LPR: localized plasmon resonance
SPR: surface plasmon resonance
ssSTED: scattering saturation STED microscopy
Cy5: cyanine 5
TGA: thiogycolic acid
DHLA: dihydrolipoic acid
NHS: *N*-hydroxysuccinimide
EDC: 1-(3-(dimethylamino)propyl)-3-ethylcarbodiimide
DIC: diisopropyl carbodiimide
SPDP: *N*-succinimidyl 3-[2-pyridyldithio]-propionate
sulfo-SMCC: sulfosuccinimidyl-(4-*N*-maleimidomethyl)cyclohexane-1-carboxylate
PEG: polyethylene glycol
PLGA: poly(lactic-*co*-glycolic acid)
EGF: epidermal growth factor
EGFR: EGF receptor
GPCR: G protein-coupled receptor
